# α-Amino bicycloalkylation through organophotoredox catalysis†

**DOI:** 10.1039/d4sc01368a

**Published:** 2024-06-04

**Authors:** Jeremy Nugent, Adrián López-Francés, Alistair J. Sterling, Min Yi Tay, Nils Frank, James J. Mousseau, Fernanda Duarte, Edward A. Anderson

**Affiliations:** a Department of Chemistry, Chemistry Research Laboratory, University of Oxford 12 Mansfield Road Oxford OX1 3TA UK fernandaduartegonzalez@chem.ox.ac.uk edward.anderson@chem.ox.ac.uk; b Department of Organic Chemistry I, Faculty of Pharmacy and Lascaray Research Center, University of the Basque Country, UPV/EHU Paseo de la Universidad 7 01006 Vitoria-Gasteiz Spain; c Pfizer Worldwide Research and Development Eastern Point Road, Groton Connecticut 06340 USA

## Abstract

Bridged bicycloalkanes such as bicyclo[1.1.1]pentanes (BCPs) and bicyclo[3.1.1]heptanes (BCHeps) are important motifs in contemporary drug design due to their potential to act as bioisosteres of disubstituted benzene rings, often resulting in compounds with improved physicochemical and pharmacokinetic properties. Access to such motifs with proximal nitrogen atoms (*i.e.* α-amino/amido bicycloalkanes) is highly desirable for drug discovery applications, but their synthesis is challenging. Here we report an approach to α-amino BCPs and BCHeps through the visible-light enabled addition of α-amino radicals to the interbridgehead C–C bonds of [1.1.1] and [3.1.1]propellane respectively. The reaction proceeds under exceptionally mild conditions and displays broad substrate scope, providing access to an array of medicinally-relevant BCP and BCHep products. Experimental and computational mechanistic studies provide evidence for a radical chain pathway which depends critically on the stability of the α-amino radical, as well as effective catalyst turnover.

## Abstract 2

The organophotocatalysed-generation of α-amino radicals enables the synthesis of α-amino bicyclo[n.1.1]alkanes (*n* = 1, 3) under mild conditions by addition to the interbridgehead C–C bonds of [n.1.1]propellanes.

## Graphical abstract

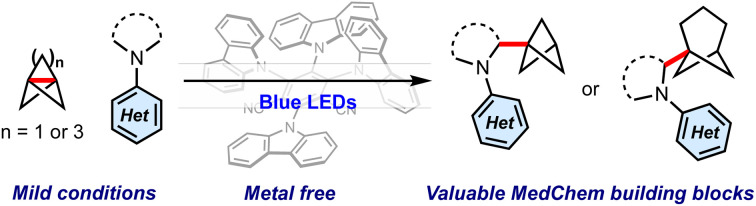

## Introduction

**Fig. 1:**
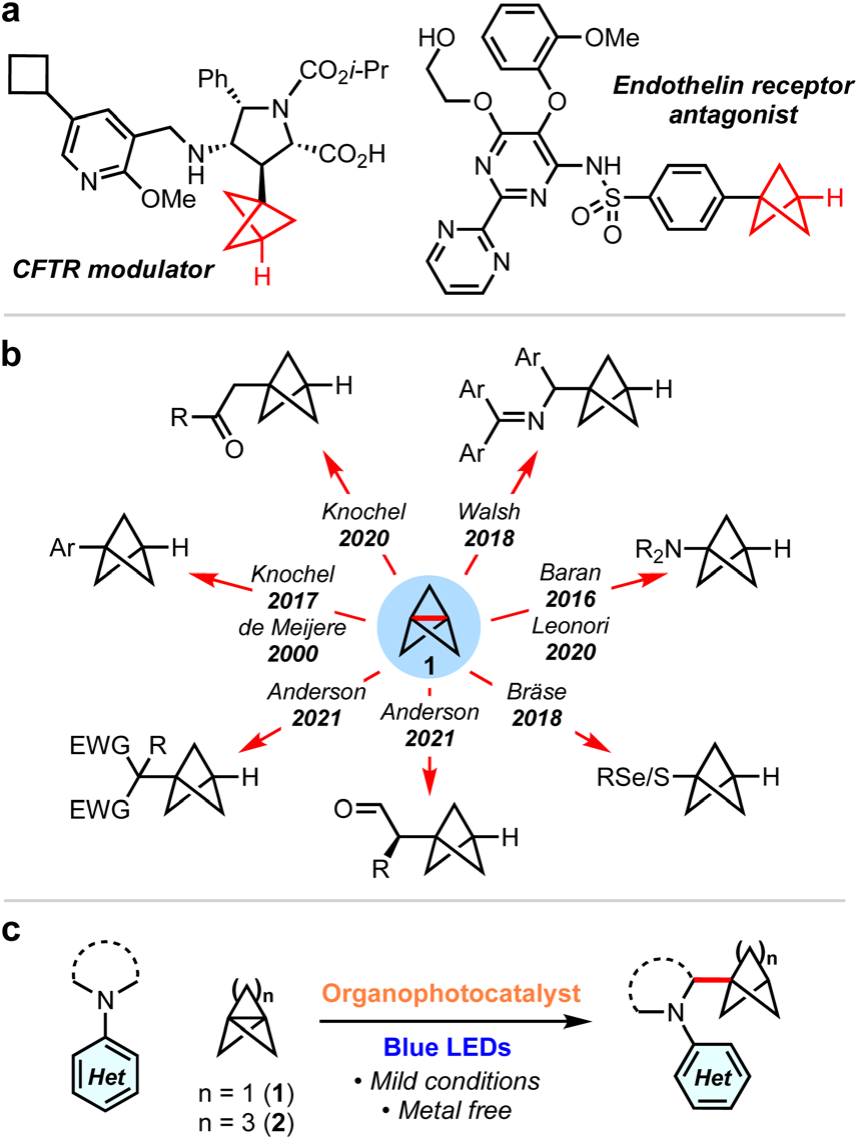
(a) Examples of monosubstituted BCPs in drug discovery. (b) Synthesis of mono-substituted BCPs from [1.1.1]propellane. (c) This work: synthesis of α-amino BCPs and BCHeps by addition of α-amino radicals to propellanes.

sp^3^-Rich ‘cage’ hydrocarbons are becoming increasingly commonplace in contemporary drug design due to their beneficial physiochemical properties compared to ‘classic’ drug functionalities such as benzene rings.^1–3^ Compounds featuring these rigid scaffolds often exhibit improved pharmacological profiles compared to their parent structure, such as resistance to metabolism, while increasing three-dimensionality.^4–7^ For example, 1,3-disubstituted BCPs are often deployed as bioisosteres for *para*-substituted arenes and alkynes, as they retain the specific positioning of substituents (180°),^8–12^ while their use as general property-enhancing motifs is also emerging.^13–15^ Similarly, monosubstituted BCPs are desirable as surrogates for phenyl and *t*-butyl groups (Fig. 1a).^16,17^ Recently, we reported the generation of the homologous bicyclo[3.1.1]heptanes (BCHeps), and described their use as potential bioisosteres of *meta*-substituted arenes, in which the bridgehead substituent vectors faithfully replicate those of the aromatic ring (∼120°).^18^

These important rigid scaffolds are typically derived from [n.1.1]propellanes, which are convenient building blocks due to the diversity of functionality that can be introduced during ring-opening of the central C–C bond, especially using radicals^19–32^ and, for [1.1.1]propellane, anions.^33–38^ In the case of mono-substituted BCPs, synthetic approaches are most commonly anionic in nature (Fig. 1b); examples include the addition to 1 of aryl Grignard reagents,^12^ turbo amides,^34,35^ enolates,^39^ dithiane^36^ and azaallyl^37,38^ anions. While these methods provide ready access to valuable BCP building blocks, they are moisture-and/or air-sensitive and thus display limited functional group tolerance. Single electron strategies also enable the synthesis of monosubstituted BCPs, but have generally been limited to electron-deficient or thiyl (and related) radicals.^24–28^

α-Amino BCPs are highly desirable in medicinal chemistry as analogues of benzylamines – motifs found in many pharmaceuticals.^40^ The synthesis of these potentially valuable compounds has been mostly overlooked, with the few reported examples requiring lengthy reaction sequences, pyrophoric/strongly basic reagents, or being limited to 1° amines.^37,41–44^ As such, the synthesis of α-amino BCPs or BCHeps directly from [1.1.1]propellane 1 and [3.1.1]propellane 2 respectively represents an attractive yet unexplored route – especially in the latter case, as anionic additions to 2 are unfeasible.^37^ While these propellanes are well-established to react efficiently with electrophilic radicals,^13,25,45,46^ the addition of nucleophilic radicals is less studied.^47^ We questioned whether the direct addition of nucleophilic α-amino radicals (generated *via* photoredox-catalysed oxidation of simple *N*,*N*-dialkylanilines)^48–58^ to propellanes 1 or 2 could generate these useful α-amino bicycloalkanes in a single step. Here we report the successful development of this methodology, which represents the first examples of the ring-opening of [1.1.1] and [3.1.1]propellanes using α-amino radicals. We complement the development of this chemistry with a detailed mechanistic study that investigates the role of each reaction component, including the source of the BCP/BCHep bridgehead hydrogen atom.

## Results and discussion

We began our investigations with the reaction of [1.1.1]propellane (1) with *N*-phenylpyrrolidine (3a, 5 equiv.) in the presence of the moderately oxidising photocatalyst Ir[(dF(CF_3_)ppy)_2_dtbbpy]PF_6_ ([Ir]1, *E*°(Ir(iii)*/Ir(ii)) = +1.21 V *vs.* SCE)^59^ in MeCN (0.5 M) under 455 nm blue LED irradiation. We were pleased to find that the desired BCP product 4a was delivered in 24% yield, along with 6% of the ‘staffane’ product 5a, which results from addition of the initially formed BCP radical to another molecule of 1 (Table 1, entry 1). A solvent screen revealed that DMF, DCE and DMA gave improved yields of 4a (36–43%, entries 2–4). Various other oxidising iridium and organophotocatalysts were investigated; while the use of [Ir]2*E*°(Ir(iii)*/Ir(ii) = +1.32 V *vs.* SCE)^59^ or [Ir]3 led to decreased yields (entries 5 and 6), the organophotocatalyst 4CzIPN (*E*° = 1.35 V *vs.* SCE)^60^ afforded 4a in 45% yield with 11% of 5a (entry 7). Additives including a range of H-atom sources and bases were not beneficial (see the ESI, Table S1†), but pleasingly an enhanced yield of product (60%, entry 8) and ratio of 4a : 5a (6 : 1) could be obtained by increasing the stoichiometry of aniline 3a to 10 equiv. Changing to a stronger light source (30 W, 440 nm) further increased the yield of 4a and permitted a shorter reaction time (65%, entry 9). The addition of 10 equiv. of water marginally increased the yield of the desired product to 70% (entry 10). Conducting the reaction under an atmosphere of air resulted in slightly reduced yields (entry 11), while control experiments demonstrated that both photocatalyst and light were required to afford high yields of 4a (entries 12 and 13). We found that, if desired, unreacted amine 3a could be recovered in near quantitative yield *via* chromatographic purification.

**Table 1:** Optimisation of the addition of α-amino radicals to [1.1.1]propellane

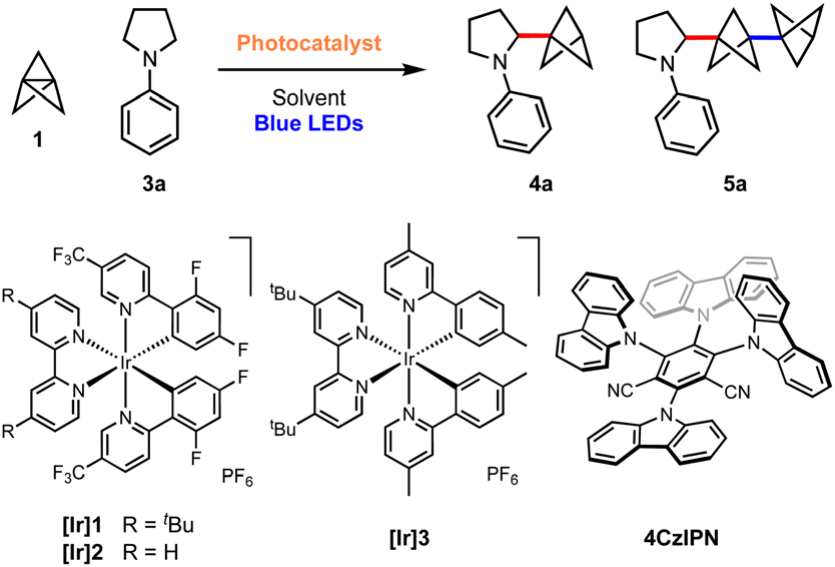						
Entry	PC	Solvent	Amine eq.	Light source	Time (h)	Yield^a^ (%) (4a : 5a)
1	[Ir]1 b	MeCN	5	18 W 455 nm	48	25 (4 : 1)
2	[Ir]1 b	DMF	5	18 W 455 nm	48	42 (4 : 1)
3	[Ir]1 b	DCE	5	18 W 455 nm	48	43 (4 : 1)
4	[Ir]1 b	DMA	5	18 W 455 nm	48	36 (4 : 1)
5	[Ir]2 b	DMA	5	18 W 455 nm	48	15 (4 : 1)
6	[Ir]3 b	DMA	5	18 W 455 nm	48	35 (4 : 1)
7	4CzIPN c	DMA	5	18 W 455 nm	48	45 (4 : 1)
8	4CzIPN c	DMA	10	18 W 455 nm	48	60 (6 : 1)
9	4CzIPN c	DMA	10	30 W 440 nm	24	65 (6 : 1)
**10**	**4CzIPN c** ^**,**^ **d**	**DMA**	**10**	**30 W 440 nm**	**24**	**70 (6 : 1)**
11	4CzIPN c^,^^d^^,^^e^	DMA	10	30 W 440 nm	24	62 (6 : 1)
12	4CzIPN c^,^^d^	DMA	10	None	24	<5
13	None^d^	DMA	10	30 W 440 nm	24	20 (6 : 1)

a Yield determined by ^1^H NMR spectroscopy using trimethoxybenzene as internal standard.

b 1 mol% of catalyst.

c 2.5 mol% of catalyst.

d 10 equiv. of water added.

e Under air. PC = photocatalyst.

**Scheme 1:**
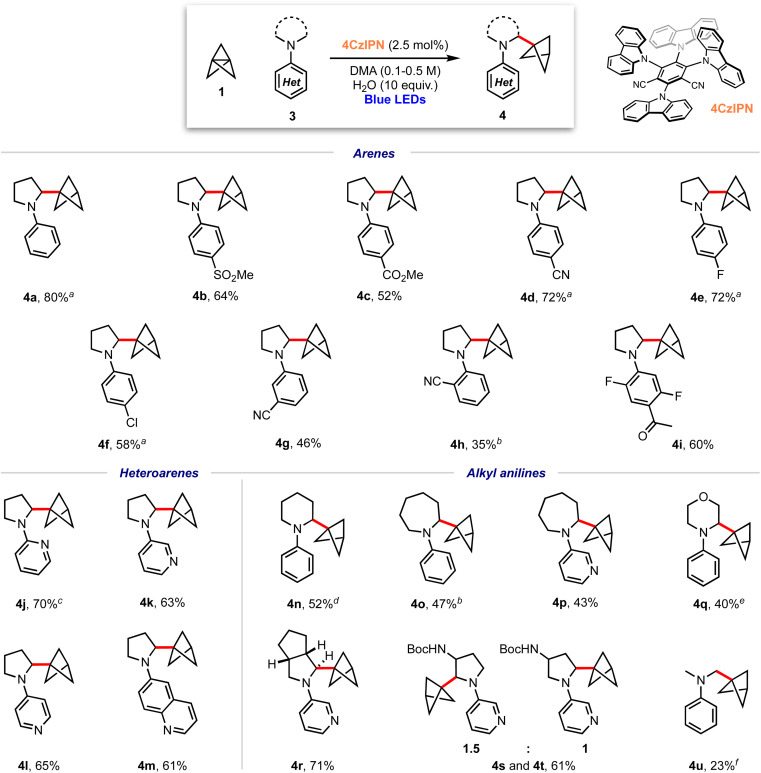
Substrate scope for α-amino bicyclo[1.1.1]pentylation reaction; isolated yields shown. ^a^ Isolated as a 6 : 1 mixture with 5, ^b^ isolated as a 5 : 1 mixture with 5, ^c^ isolated as a 12 : 1 mixture with 5, ^d^ isolated as a 4 : 1 mixture with 5; ^e^ isolated as a 10 : 1 mixture with 5; ^f^ isolated as a 3 : 1 mixture with 5.

With optimised conditions in hand, the scope of the aniline coupling partner was investigated, focussing first on variation of the *N*-arene substituent. We found this method of BCP installation to be successful with diversely functionalised (hetero)arylpyrrolidine substrates, with the α-amino BCP products generally obtained in good yields (Scheme 1). Electron-neutral and electron-poor *para*-substituted aniline substrates are well-suited to this reaction and gave good-to-excellent yields of the desired α-amino BCP products (4a–f, 52–80%). Substitution at the *meta*- and *ortho*-positions was also tolerated (4g–h, 35–46%), as were trisubstituted anilines (4i, 60%). The synthesis of BCPs substituted with heteroaryl dialkylanilines would be of high interest in a pharmaceutical context; pleasingly, we found that 2-, 3-, and 4-pyrrolidinopyridines were excellent substrates for this reaction, affording BCP products in high yields (4j–l, 63–70%); similarly a pyrrolidine–quinoline derivatives gave the BCP product 4m in good yield (61%).

We next investigated substrates in which the dialkylamine was varied. Pleasingly, piperidine (4n, 52%), azepane (4o–p, 43–47%) and morpholine (4q, 40%) substituted (hetero)arene BCPs were isolated in good yields, albeit with a slight increase in the amount of staffane side-product. Substrates bearing substituted pyrrolidines proved more challenging: while hexahydrocyclopenta[*c*]pyrrole 4q was obtained in excellent yield (71%), the use of non-symmetric substrates resulted in mixtures of product regioisomers, although high yields were still obtained (*e.g.*4r and 4s, 61%). For reasons that are unclear, acyclic dialkylamine systems generally resulted in low yields, with significant amounts of staffane formation (4u, 23%).

**Scheme 2:**
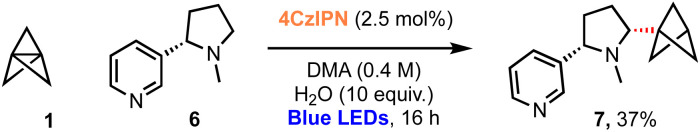
Bicyclopentylation of nicotine (6, 10 equiv.); 7 was isolated as a 4 : 1 mixture with the corresponding BCP-staffane.

Recent studies suggest that nicotine may exert neuroprotective effects inducing defence mechanisms against pathologies associated with Alzheimer's or Parkinson's disease.^19–28^ Pleasingly, use of nicotine (6, Scheme 2) as a substrate for this α-aminobicyclopentylation reaction led to the corresponding BCP–nicotine derivative 7 (37%), highlighting the applicability of the chemistry to drug molecules. C–H abstraction occurs preferentially at the 2° position adjacent to the nitrogen atom due to the stability of the resulting α-amino radical.^61^ While a 3° radical at the opposing α-position should be significantly more stable than any of the 1° or 2° radicals that could be formed, we suggest that steric repulsion encountered during the C–H abstraction process prevents the formation of this radical, rationalising the observed regioselectivity of the reaction.

Considering the excellent results obtained in the bicyclopentylation reactions of 1 to form α-amino BCPs, and the high relevance of recently discovered BCHeps as potential bioisosteres of *meta*-substituted arenes,^13^ we questioned whether [3.1.1]propellane 2 would also perform well in this nucleophilic radical addition chemistry. We first studied the stability of 2 under blue LED irradiation (440 nm), which confirmed that 2 is stable for several hours.^61^ Under the optimised conditions developed for the bicyclopentylation, we explored the reaction of 2 with aniline 3a. The use of 1 equiv. of amine 3a under the optimized reaction conditions afforded the desired product 8a in low yield (18%, Table 2, entry 1), as a 4 : 1 mixture with the corresponding BCHep staffane 9a (which notably constitutes the first example of this type of ‘dimer’ formation for [3.1.1]propellane).^13^ An increase in yield but a similar product-staffane ratio was observed using 5 equiv. of amine 3a (47% (4 : 1), entry 2), while the use of 10 equiv. of amine resulted in enhancement of both the yield and product : staffane ratio (82% (5 : 1), entry 3).

**Table 2:** Optimisation of the addition of α-amino radicals to [3.1.1]propellane^a^

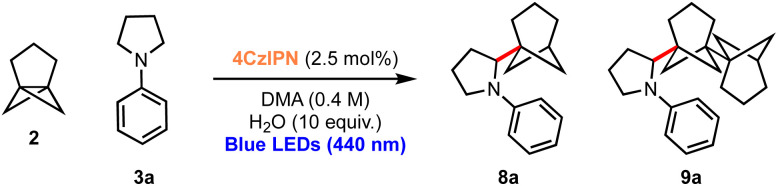			
Entry	Amine equiv.	Time (h)	Isolated Yield (8a : 9a)^a^
1	1	16	18% (4 : 1)
2	5	16	47% (4 : 1)
**3**	**10**	**16**	**82% (5 : 1)**

a Reactions conducted using 2 (1 equiv.), 3a (10 equiv.) at room temperature.

**Scheme 3:**
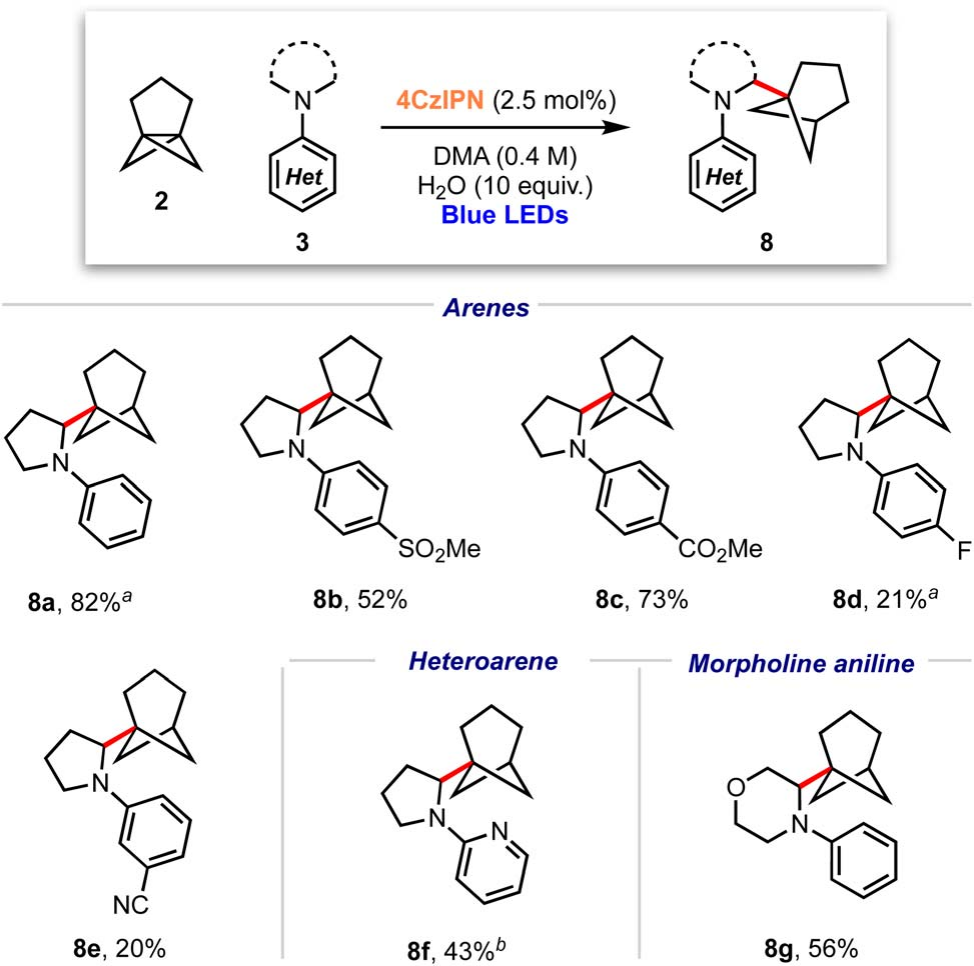
Substrate scope for α-amino bicyclo[3.1.1]heptane reaction; isolated yields shown. ^a^ Isolated as a 5 : 1 mixture with 9a (from 8a) or 9d (from 8d). ^b^ Isolated as a 10 : 1 mixture with 9f.

These conditions were applied to a range of amine substrates (Scheme 3). We observed that anilines featuring neutral and electron-withdrawing *N*-aryl groups were well-tolerated, affording the corresponding α-amino BCHeps in good to excellent yields (8a–c, 52–82%), while more electron-deficient *N*-aryls (*para*-fluorine or *meta*-cyano substitution) led to low yields of BCHep product (8d–e, 20–21%). Notably, the replacement of the *N*-aryl and pyrrolidine rings with pyridine or morpholine motifs respectively was successful, generating BCHeps that feature multiple ‘drug-like’ functionalities (8f–g, 43–56%). Interestingly, only phenyl, *para*-fluorophenyl and pyridine corresponding BCHep staffanes (9a, 9d, 9f); staffane formation was not observed for other substrates.

### Mechanistic studies

**Fig. 2:**
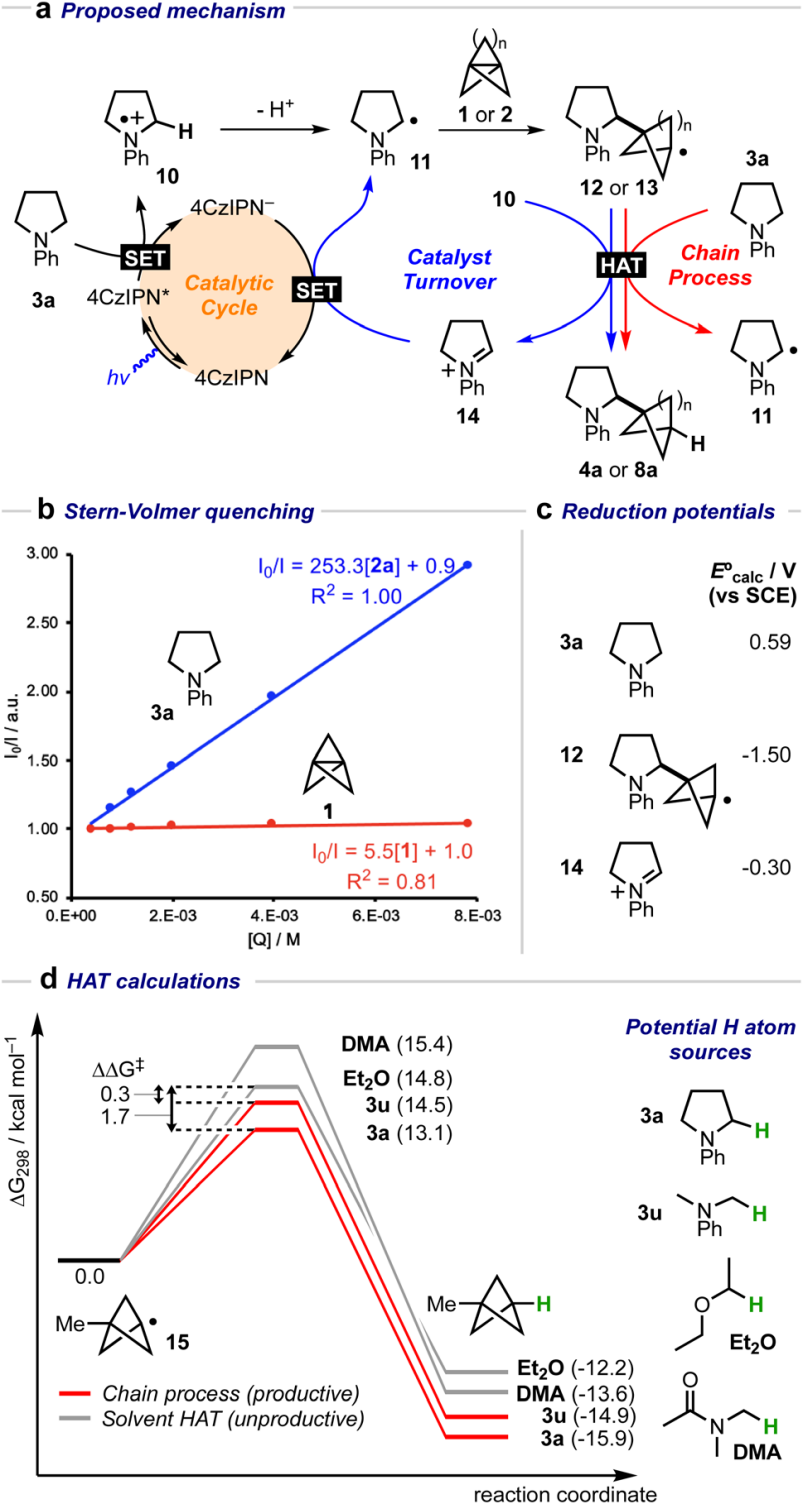
(a) Proposed mechanistic cycle for the formation of α-amino BCPs from amines and [1.1.1]propellane 1 (*n* = 1) or [3.1.1]propellane 2 (*n* = 3). (b) Stern–Volmer quenching plot for amine 3a and [1.1.1]propellane 1 with 4CzIPN. (c) Calculated *E*° values (V *vs.* SCE) for 3a, 12 and 14. (d) Calculated H-atom transfer barriers using amines 3a, 3u, DMA, and Et_2_O.^64^ Free energies were calculated at 298.15 K, and the standard concentration of each species was adjusted for the experimental molar ratios (3a/3u: 10.0 equiv., DMA: 21.6 equiv. Et_2_O: 12.0 equiv.).

A mechanistic cycle is proposed in Fig. 2a. Initial excitation of the photocatalyst gives a highly oxidising species (*E*° = 1.35 V *vs.* SCE)^62^ which is capable of oxidising the amine 
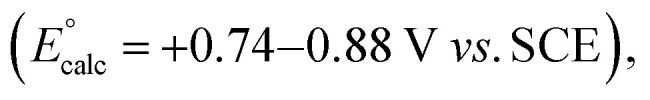
^63^ followed by deprotonation of the resulting radical cation 10 (by excess 3a) to form an α-amino radical 11. This proposal is supported by a Stern–Volmer quenching study in which the amine 3a quenches the luminescence of the photocatalyst with >40 times the efficiency of 1 (Fig. 2b). The resulting α-amino radical can add to the inter-bridgehead bond of [1.1.1]propellane to form a bridgehead BCP radical 12, which can then either abstract an H atom from the α-position of a second molecule of the amine (to propagate a chain process), or from radical cation 10, or from the solvent; these HAT processes are in competition with staffane formation. Catalyst turnover can then be achieved by reduction of iminium ion 14. An alternative fate for the BCP radical could be reduction by the reduced photocatalyst (*E*° = −1.21 V *vs.* SCE)^62^ to complete the catalytic cycle, followed by quenching of the BCP anion by water present in the reaction; however, calculations suggest that reduction of BCP radical by the 4CzIPN radical anion would be an approximately thermoneutral process (Δ*E*_calc_ = 0.17 V, Fig. 2c) and may therefore be outcompeted by alternative low-barrier processes such as HAT. Further calculations (Fig. 2d) identified transition state barriers for hydrogen-atom transfer to a methyl-substituted BCP radical 15 with *N*-phenylpyrollidine (3a) (Δ*G*^‡^ = 13.1 kcal mol^−1^), Et_2_O (Δ*G*^‡^ = 14.8 kcal mol^−1^) and DMA (Δ*G*^‡^ = 15.4 kcal mol^−1^) at potential hydrogen atom sources.^64^ The lower barrier of the former of these (3a) can be attributed to the greater stability of the developing α-amino radical over the corresponding Et_2_O/DMA radicals. The importance of this radical stability is shown by the use of dimethylaniline 3u as the HAT source: its 1° α-amino radical is 1.0 kcal mol^−1^ less stable than the 2° radical derived from 3a, and the barrier to HAT increases to 14.5 kcal mol^−1^. The radical chain process is then in closer competition with HAT from the solvent (ΔΔ*G*^‡^ = 0.3 kcal mol^−1^), which we hypothesise could decrease the radical chain length, and may be the cause of the poorer yield (of 4u, 23%), and product : staffane ratio (3 : 1), observed for this substrate.

The preference for HAT transfer from 3a, rather than the solvent or H_2_O, was further explored using deuterium-labelling studies (Table 3). We first confirmed that under the standard conditions, no deuterium incorporation was observed in the presence of D_2_O, ruling out the reduction of the BCP radical as a catalyst turnover step (entry 2). Use of d_7_-DMF (as a surrogate for DMA) also led to no product deuteration (entry 3). However, 80% D-incorporation was observed using d_4_-*N*-phenylpyrrolidine (entry 4, d_4_-3a), albeit this reaction proceeded in very low yield. A significantly greater amount of staffane was observed, which is consistent with the slower rate of deuterium atom transfer compared to HAT with h_4_-3a (4a : 5a = 1.3 : 1 *vs.* 6.4 : 1). Only 34% D-incorporation was observed when using d_4_-*N*-phenylpyrollidine in combination with a DMA/Et_2_O solvent mixture (entry 5), suggesting that these solvents may also act as H-atom sources in the presence of deuterated substrate.

**Table 3:** Deuterium-labelling studies

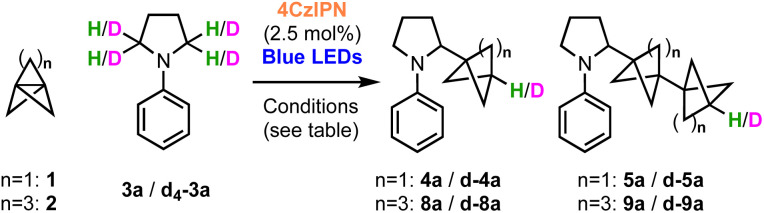					
Entry	Substrates	Solvent	Additive	% D	Yield (%) (4a : 5a)/(8a : 9a)
1	1 + h_4_-3a	DMA/Et_2_O	H_2_O	0	70 (6.4 : 1)
2	1 + h_4_-3a	DMA/Et_2_O	D_2_O	0	70 (6.4 : 1)
3	1 + h_4_-3a	d_7_-DMF/pentane	D_2_O	0	70 (6.4 : 1)
4^a^	1 + d_4_-3a	d_7_-DMF/pentane	D_2_O	80	7 (1.3 : 1)
5^a^	1 + d_4_-3a	DMA/Et_2_O	D_2_O	34	10 (1.6 : 1)
6^a^	1 + d_4_-3a	d_7_-DMF/Et_2_O	D_2_O	63	10 (1.5 : 1)
7	2 + h_4_-3a	DMA/*n*-Bu_2_O	H_2_O	0	82 (5 : 1)
8^a^	2 + d_4_-3a	DMA/*n*-Bu_2_O	H_2_O	38	19 (3.5 : 1)
9	2 + h_4_-3a	DMA/*n*-Bu_2_O	D_2_O	21	79 (5 : 1)
10^a^	2 + d_4_-3a	DMA/*n*-Bu_2_O	D_2_O	49	10 (3.5 : 1)
11^a^	2 + d_4_-3a	d_7_-DMF/*n*-Bu_2_O	D_2_O	74	8 (2 : 1)

a d_4_-3a = 98% D.

Equivalent deuteration studies were next performed using [31.1]propellane 2 as acceptor, which confirmed that the substrate 3a is again a capable H atom donor, with 38% D-incorporation using d_4_-*N*-phenylpyrrolidine, DMA and H_2_O (entries 7 and 8). However, additional experiments revealed that in the case of 2, use of D_2_O resulted in a surprising 21% D-incorporation and a much superior yield (entry 9). Furthermore, 49% deuteration was observed using a combination of d_4_-3a and D_2_O (entry 10), and the introduction of d_7_-DMF further increased the extent of deuteration to 74%, confirming the participation of multiple H-atom sources, including the solvent (entry 11). The BCHep/staffane ratios gradually decreased from 5 : 1 (entries 7 and 8), progressing to 3.5 : 1 (entries 9 and 10), and finally reaching 2 : 1 (entry 11), showing that the bicycloheptylation reaction also features a fine balance between HAT and staffane formation.

Additional evidence for our mechanistic proposal was obtained using the kinetic isotope effect (KIE) observed for the HAT step in the reactions of 1. Since staffane formation is independent of the deuteration state of the amine, the product:staffane ratio for h_4_- and d_4_-*N*-phenylpyrollidine (Table 3, entries 1 *vs.* 4) should approximate the HAT KIE, *i.e. k*_H_/*k*_D_ ≃ (4a : 5a)/(d-4a : d-5a) (see ESI† for further discussion). Using this approach, a KIE of 4.9 ± 0.5 was obtained, which is in reasonable agreement with the value obtained from computation (*k*_H_/*k*_D_ = 6.1).

While a chain process is evidently possible, the low quantum yield of 0.84 obtained for this reaction suggests catalyst turnover is important.^61^ Since reduction of the BCP radical is not viable to achieve turnover (at least for [1.1.1]propellane, as evidenced by the lack of deuteration of 4a in the presence D_2_O for this propellane), it may be that a fast HAT process between BCP radical 12 and low-concentration radical cation 10 could occur that would lead to the formation of iminium ion 14. This iminium ion could then be reduced by 4CzIPN^−^ to reform the neutral organophotoredox catalyst, and an α-amino radical 11 (Δ*E*_calc_ = +0.88 V).

**Scheme 4:**
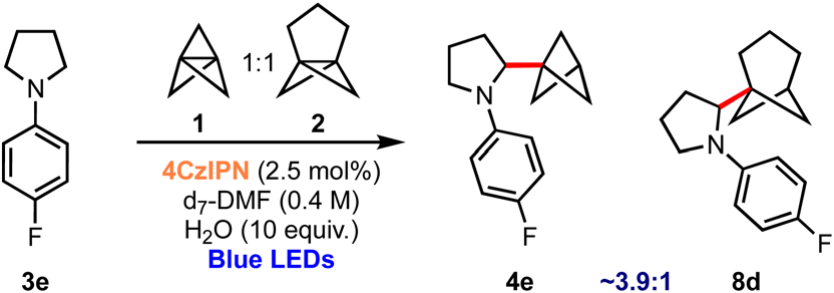
Competition experiment: [1.1.1]propellane 1*vs.* [3.1.1]propellane 2.

Finally, the successful use of different propellanes in this aminobicycloalkylation offers the opportunity to compare the relative propensity of the two to undergo ring-opening. A competition experiment was therefore undertaken in which the reaction was conducted using an equimolar mixture of 1 and 2; this experiment revealed that the formation of BCP derivative 4e is ∼3.9 times faster than BCHep product 8d as judged by NMR monitoring of the proportions of products formed during the reaction.^61^ This reveals an enhanced reactivity of [1.1.1]propellane 1 compared to [3.1.1]propellane 2, at least in this particular setting of nucleophilic radical addition chemistry (Scheme 4).

## Conclusions

In conclusion, we have developed an organocatalysed photoredox approach for the synthesis of α-amino BCPs and BCHeps through the addition of α-amino radicals to the strained interbridgehead of [1.1.1] and [3.1.1]propellanes respectively. The reaction displays scope that is of high relevance in medicinal chemistry research, where such motifs are of high importance. A combination of experimental and computational mechanistic studies provide evidence for a radical chain pathway, and also offer insight into the kinetics of hydrogen atom transfer steps of bridgehead bicycloalkyl radicals.

## Data availability

The datasets supporting this article have been uploaded as part of the ESI.†

## Author contributions

J. N. and E. A. conceived the project. J. N., A. L.-F., A. J. S., M. Y.-T. carried out the experimental work. A. J. S. and N. F. carried out the computational work. J. N., J. J. M., F. D. and E. A. directed the project. J. N., A. L.-F., A. J. S. and E. A. wrote the manuscript. All authors contributed to editing and revision of the manuscript.

## Conflicts of interest

There are no conflicts to declare.

## Supplementary Material

SC-015-D4SC01368A-s001

SC-015-D4SC01368A-s002
