# Supplementary material for: α-Amino bicycloalkylation through organophotoredox catalysis
Source: Chem Sci. 2024 Jun 4;15(28):10918–25. doi: 10.1039/d4sc01368a (PMC11253163; doi:10.1039/d4sc01368a)
Supplement: SC-015-D4SC01368A-s002 [file SC-015-D4SC01368A-s002.pdf]

Electronic Supplementary Material (ESI) for Chemical Science.
This journal is © The Royal Society of Chemistry 2024

α-Amino Bicycloalkylation through Organophotoredox Catalysis

Jeremy Nugent,[a] Adrián López-Francés,[b] Alistair J. Sterling,[a] Tay Min Yi,[a] Nils Frank,[a] James J. Mousseau,[c] Fernanda Duarte*[a] and Edward A. Anderson*[a]

[a]*Chemistry Research Laboratory, Department of Chemistry, University of Oxford, 12 Mansfield Road, Oxford, OX1 3TA, U.K.*

[b]*Department of Organic Chemistry I, Faculty of Pharmacy and Lascaray Research Center, University of the Basque Country, UPV/EHU. Paseo de la Universidad 7, 01006 Vitoria-Gasteiz, Spain*

[c]*Pfizer Worldwide Research and Development, Eastern Point Road, Groton, Connecticut 06340, United States*

Supporting Information

Table of Contents

1. General Experimental Considerations S2
2. General Procedures S3
3. Reaction Optimization S4
4. Low Yielding and Unsuccessful Substrates S5
5. Photochemical Set-up S6
6. [3.1.1]Propellane Stability Experiment S7
7. Deuterium-labelling studies S8
8. Kinetic isotope effect studies S9
9. Competition experiment: [1.1.1]Propellane vs [3.1.1]Propellane S11
10. Photophysical experiments S13
11. Experimental Procedures S17
    a) Synthesis of Bicyclo[1.1.1.]pentanes S18
    b) Synthesis of Bicyclo[3.1.1.]heptanes S37
    c) Substrate Synthesis S45
12. Computational details S49
13. NMR Spectra S56
14. References S96

1. General Experimental Considerations

NMR Spectroscopy: ^{1}H, ^{13}C and ^{19}F NMR spectra were recorded on Bruker AV400, Bruker AVII500 or Bruker NEO 600 spectrometers using TOPSPIN software, with the deuterated solvent acting as the internal deuterium lock. ^{1}H NMR spectra were recorded at 400, 500 or 600 MHz, ^{13}C NMR spectra were recorded at 101, 126 or 151 MHz with ^{1}H decoupling, and ^{19}F NMR spectra were recorded at 376 or 470 MHz. Assignments were determined either on the basis of unambiguous chemical shift / coupling patterns, or from 2D COSY, HMBC, HSQC and / or NOESY experiments. Peak multiplicities are defined as: s = singlet, d = doublet, t = triplet, q = quartet, quin. = quintet, m = multiplet, br. = broad, app. = apparent; coupling constants (*J*) are reported to the nearest 0.1 Hz.

Infrared Spectroscopy: Infrared spectra were recorded on a Bruker Tensor 27 FT-IR spectrometer with the sample being prepared as a thin film on a diamond ATR module. Absorption maxima (ν_{max}) are quoted in wavenumbers (cm^{-1}).

High Resolution Mass Spectrometry: Mass Spectrometry: Low resolution mass spectra were recorded on a Micromass LCT Premier Open Access using electrospray ionization (ESI). Accurate mass (HRMS) data was determined under conditions of ESI, EI and CI on a Bruker MicroTOF. High resolution values are calculated to 4 decimal places from the molecular formula, and all values are within a tolerance of 5 ppm.

Melting Points: Melting points were obtained using a Griffin melting point apparatus and are uncorrected.

Reagents, solvents and techniques: All reagents were used directly as supplied. Solvents were either used as commercially supplied, or purified by standard techniques. Anhydrous Et_2O, CH_2Cl_2 and toluene were obtained from solvent dispenser units having been passed through an activated alumina column under argon. Unless otherwise stated, non-aqueous reactions were performed using heatgun-dried glassware under a N_2 atmosphere. Anilines were either purchased from commercial suppliers or generously provided by Pfizer.

Reactions were monitored by thin layer chromatography on pre-coated aluminium-backed plates (Merck Kieselgel 60 with fluorescent indicator UV254). Spots were visualized by quenching of UV fluorescence or by staining with potassium permanganate or vanillin, and retention factors are reported with the solvent system in parentheses. Flash column chromatography was performed on silica gel obtained from Merck (Silica gel Si 60, 0.04-0.063 mm) under a positive pressure of nitrogen, using the stated solvent system.

2. General Procedures

General Procedure 1A: α-amino bicyclopentylation

To a heatgun-dried vial containing the specified (hetero)aniline (10.0 equiv.) and 4CzIPN (3.9 mg, 2.5 mol%) was added anhydrous DMA (0.4-2 mL), and deionised H_2O (36 µl, 10 equiv.). Argon was bubbled through the solution for 15 min, then [1.1.1]propellane (0.25 mL of a 0.8 M solution in Et_2O, 0.2 mmol, 1.0 equiv.) was added. The vial was sealed and irradiated with a 440 nm Kessil PR160L LED lamp from a distance of approximately 6 cm. After the indicated time the resulting solution was diluted with EtOAc (5 mL) and washed with 5% LiCl solution (10 mL). The layers were separated, and the aqueous layer was extracted with EtOAc (2 × 5 mL). The combined organic layers were washed with brine, dried over $MgSO_4$ and concentrated *in vacuo*. The crude product was then purified by column chromatography.

General Procedure 1B: α-amino bicycloheptylation

To a heatgun-dried vial the specified pyrrolidine (10.0 equiv.) and 4CzIPN (3.9 mg, 2.5 mol%) were added. The vial was evacuated and refilled with N_2 (g) three times. Under nitrogen atmosphere, anhydrous DMA (0.5 mL), deionised H_2O (36 µl, 10 equiv.) and [3.1.1]propellane (0.58 mL of a 0.35 M solution in *n*-Bu_2O, 0.2 mmol, 1.0 equiv.) were added. The vial was irradiated with a 440 nm Kessil PR160L LED lamp in an Evoluchem™ PhotoRedOx box. After the indicated reaction time, the resulting solution was concentrated under vacuum, and the crude product was then purified by column chromatography.

General Procedure 2: Ullman cross-coupling

To a heatgun-dried flask containing K_2CO_3 (5.53 g, 40.0 mmol, 2.0 equiv.), proline (461 mg, 4.0 mmol, 20 mol %), and CuI (381 mg, 2.0 mmol, 10 mol%) in anhydrous DMSO (12 mL) was added pyrrolidine (5.0 mL, 60.0 mmol, 3.0 equiv.) and (hetero)aryl halide (20 mmol, 1.0 equiv.). The resulting slurry was heated at 80 °C for the specified time then diluted with EtOAc and washed with NH_4OH (10 mL, 30% aq.). The layers were separated, and the aqueous layer was extracted with EtOAc (2 × 5 mL). The combined organic layers were washed with brine, dried over $MgSO_4$ and concentrated *in vacuo*. The crude product was then purified by column chromatography.

3. Reaction Optimization

Catalyst	Solvent (0.4 mL)	Additive	Amine Eq.	Time (h)	Yield	pdt : staffane
$Ir[(dF(CF_3)ppy)_2dtbbpy]PF_6$ (1%)	DMA	-	3	18	19%	4:1
$Ir[(dF(CF_3)ppy)_2dtbbpy]PF_6$ (1%)	DMA	-	5	18	26%	4:1
$Ir[(dF(CF_3)ppy)_2dtbbpy]PF_6$ (1%)	DMA	-	5	48	36%	4:1
$Ir[(dF(CF_3)ppy)_2dtbbpy]PF_6$ (1%)	DMA	-	5	66	38%	4:1
$Ir[(dF(CF_3)ppy)_2dtbbpy]PF_6$ (2%)	DMA	-	5	48	7%	4:1
$Ir[(dF(CF_3)ppy)_2dtbbpy]PF_6$ (0.5%)	DMA	-	5	48	45%	4:1
$Ir[(dF(CF_3)ppy)_2dtbbpy]PF_6$ (1%)	MeCN	-	5	48	25%	4:1
$Ir[(dF(CF_3)ppy)dtbbpy]PF_6$ (1%)	DCM	-	5	48	42%	4:1
Ir[(dF(CF3)ppy)dtbbpy]PF6 (1%)	MeOH	-	5	48	16%	4:1
$Ir[(dF(CF_3)ppy)dtbbpy]PF_6$ (1%)	DMF	-	5	48	42%	4:1
$Ir[(dF(CF_3)ppy)dtbbpy]PF_6$ (1%)	DME	-	5	48	30%	4:1
$Ir[(dF(CF_3)ppy)_2dtbbpy]PF_6$ (1%)	DCE	-	5	48	43%	4:1
$Ir[(dF(CF_3)ppy)_2dtbbpy]PF_6$ (0.5%)	DCE	-	5	48	40%	4:1
Ir(4'- $CF_3ppy)_3$ (1%)	DCE	-	5	48	24%	4:1
fac-$Ir(ppy)_3$ (1%)	DCE	-	5	48	20%	4:1
triphenylpyrillium tetrafluoroborate (5%)	DCE	-	5	48	25%	4:1
$Ru(bpy)_3(PF6)_2$ (1%)	DCE	-	5	48	10%	4:1
$Ru(bpy)_3(PF6)_2$ (1%)	DMA	-	5	48	3.5%	4:1
9-mesityl-10-methylacridinium tetrafluoroborate (5%)	DMA	-	5	48	0%	4:1
4CzIPN (2.5%)	DMA	-	5	48	42%	4:1
$Ir[(ppy)_2(dtbbpy)]PF_6$ (1%)	DMA	-	5	48	30%	4:1
$Ir[Me(Me)ppy]_2(dtbbpy)]PF_6$ (1%)	DMA	-	5	48	35%	4:1
4DPAIPN (5%)	DMA	-	5	48	45%	4:1
Rhodamine B (5%)	DMA	-	5	48	2.5%	4:1
Eosin Y (5%)	DMA	-	5	48	40%	4:1
4CzIPN (2.5%)	DMA	-	5	48	42%	4:1
$Ir[dF(CF_3)ppy]_2(bpy)PF_6$ (5%)	DMA	-	5	48	15%	4:1
4DPAIPN (5%)	DMA	-	5	48	47%	4:1
$Ir[(dF(CF_3)ppy)_2dtbbpy]PF_6$ (0.5%)	DMA	2-phenylmalonitrile (0.2 eq.)	5	48	18%	4:1
$Ir[(dF(CF_3)ppy)_2dtbbpy]PF_6$ (0.5%)	DMA	9-phenyl-9H-fluorene (0.2 eq.)	5	48	30%	4:1
Ir[(dF(CF3)ppy)2dtbbpy]PF6 (0.5%)	DMA	1,4-cyclohexadiene (1 eq.)	5	48	20%	4:1
Ir[(dF(CF3)ppy)2dtbbpy]PF6 (0.5%)	DMA	2,4,6-$(iPr)_3$PhSH (0.2 eq.)	5	48	10%	4:1
$Ir[(dF(CF_3)ppy)_2dtbbpy]PF_6$ (0.5%)	DMA	-	10	48	67%	6:1
4DPAIPN (2.5%)	DMA	-	10	48	65%	6:1
4CzIPN (2.5%)	DMA	-	10	48	64%	6:1
4CzIPN (5%)	DMA	-	10	48	64%	6:1

Low Yielding and Unsuccessful Substrates

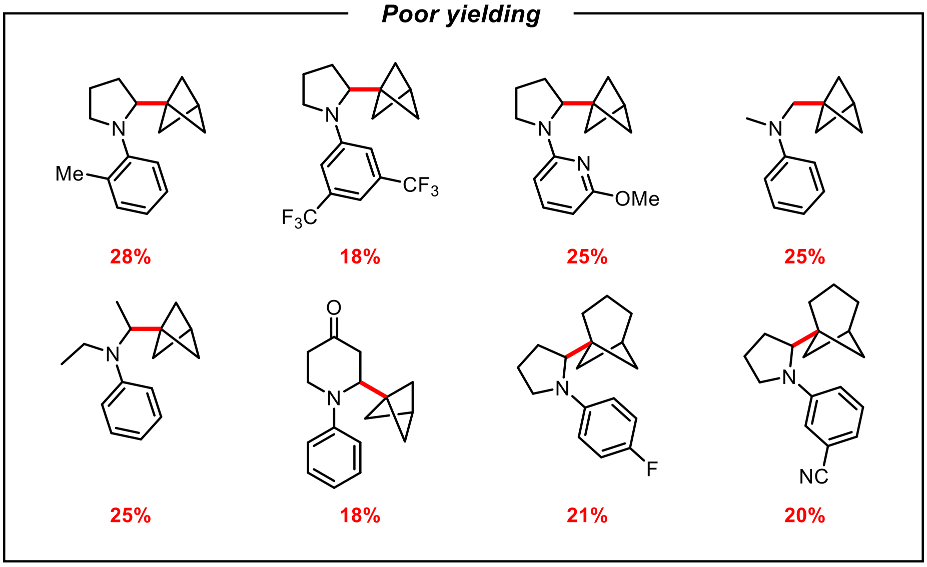

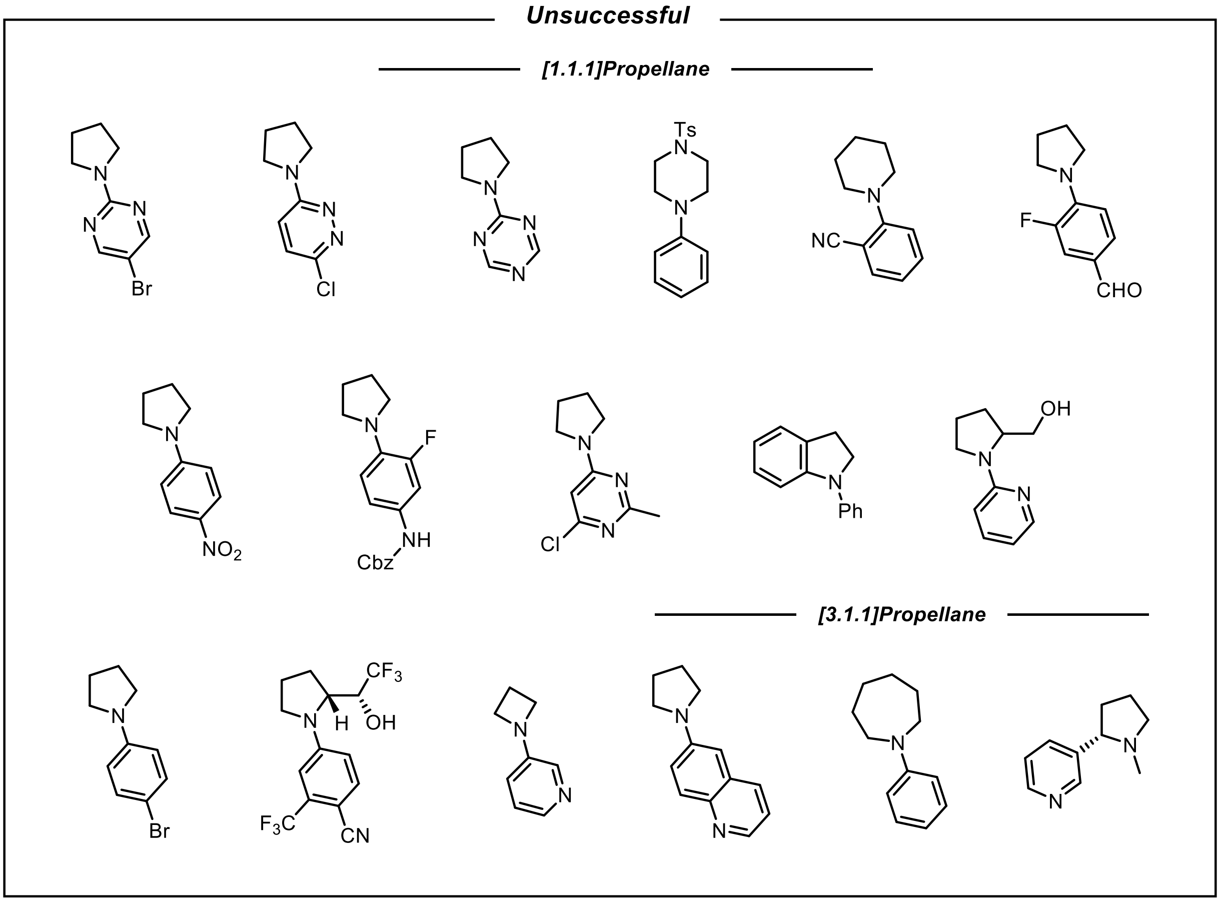

4. Photochemical Set-up

Under optimised conditions, reactions were irradiated with a Kessil PR160L 440 nm LED lamp from a distance of approximately 6 cm (Figure S1, left) or in an Evoluchem™ PhotoRedOx box (Figure S2, right).

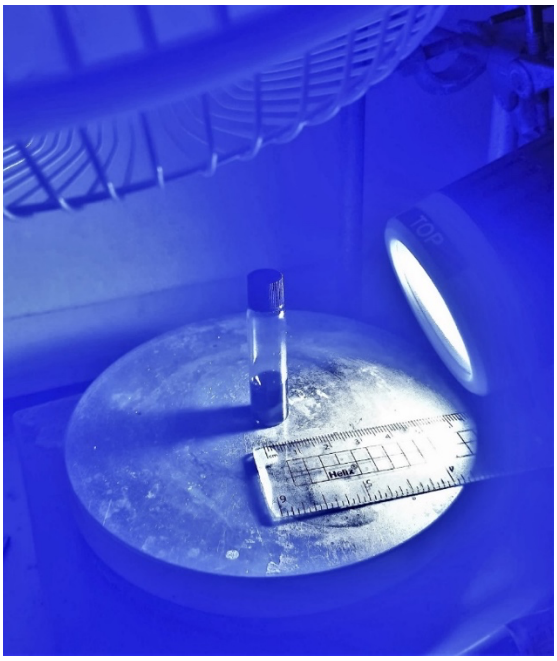

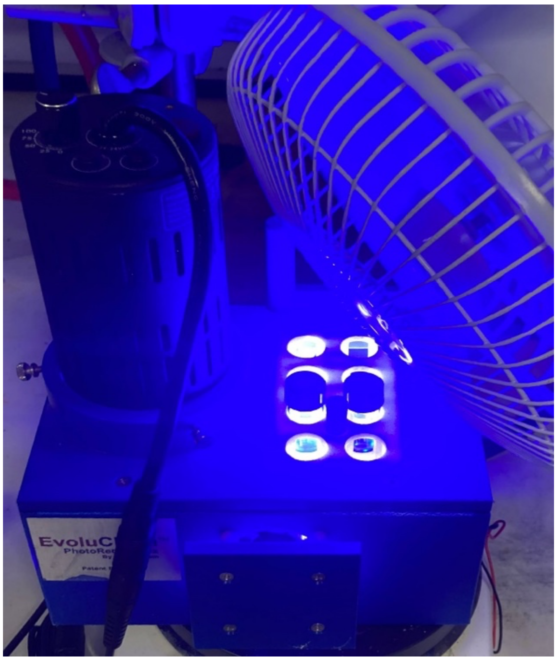

Fig. S1. Kessil PR160L 440 nm LED lamp from a distance of approximately 6 cm.

Fig. S2. Kessil PR160L 440 nm LED in an Evoluchem™ PhotoRedOx box.

5. [3.1.1]Propellane Stability Experiment

Parallel experiment: two dry septum-fitted NMR tubes were evacuated and refilled with N_2 (g) for three cycles. Then [3.1.1]propellane solution (0.20 mL), d_7-DMF (0.30 mL) and 1,2-dichloroethane (50 µL, 0.63 mmol) were added under nitrogen atmosphere in each tube. Immediately, a ^{1}H NMR spectrum was measured to determine the concentration of propellane, using 1,2-dichloroethane as internal standard. One NMR tube was expose to 440 nm blue LEDs irradiation conditions, and ^{1}H NMR spectra were taken at intervals (Table S1 / Figure S3) to calculate the residual propellane concentration. The other tube was maintained under ambient conditions, in the dark, and subjected to the same procedure to determine propellane concentration. *Note:* After 6 hours, the experiment was halted, and the NMR tubes were frozen at -20 ºC for 12 hours. No decomposition was observed during this period. Irradiation continued after this time.

Time / [h]	440 nm Blue LEDs / [mol L^{-1}]	Ambient Conditions / [mol L^{-1}]
0	0.41	0.41
1	0.38	0.41
2	0.35	0.40
3	0.33	0.40
4	0.32	0.40
5	0.31	0.39
6	0.30	0.39
8	0.29	0.38
10	0.27	0.37
16	0.24	0.36

Table S1. Decomposition of [3.1.1]propellane in d_7-DMF under 440 nm blue LED irradiation.

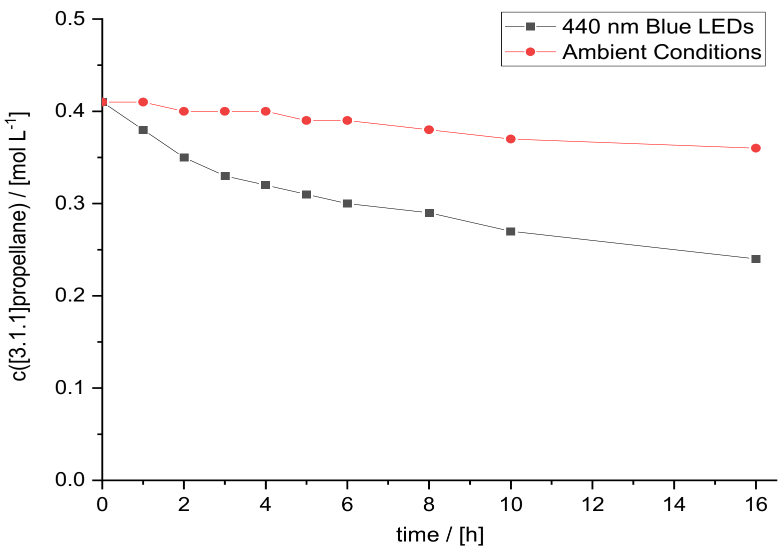

Fig. S3. Decomposition of [3.1.1]propellane in D7-DMF under 440 nm blue LED irradiation.

6. Deuterium-labelling studies

The general procedures for α-amino bicyclopentylation (General Procedure 1A) and α-amino bicycloheptylation (General Procedure 1B), were applied using *N*-phenylpyrollidine (h_4-PhPyr: 294 mg, 2.0 mmol, 10.0 eq.) or d_4-*N*-phenylpyrollidine (d_4-PhPyr (98% D): 302 mg, 2.0 mmol, 10.0 eq.) based on the specific deuteration experiment (Table S2). In certain instances, d_7-DMF (0.5 mL) was used as the solvent instead of DMA, and/or H_2O was replaced with D_2O (40 µl, 10 equiv.) (Table S2).

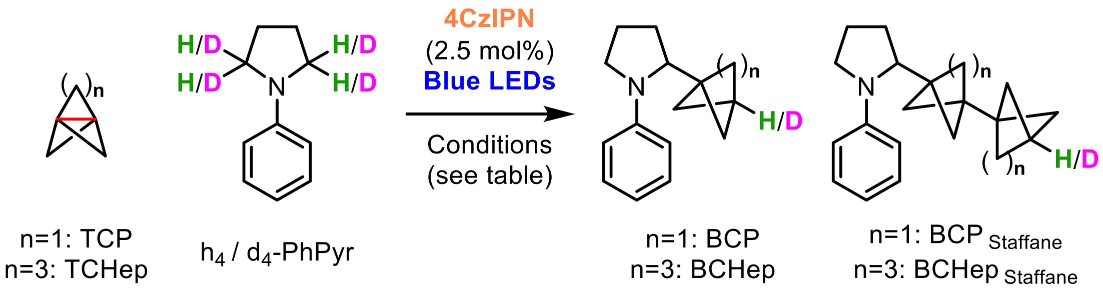

Substrates	Solvent	Additive	%D	Yield (%)
TCP + h_4-PhPyr	DMA/Et_2O	H_2O	0	70 (6.4:1)[a]
TCP + h_4-PhPyr	DMA/Et_2O	D_2O	0	70 (6.4:1)[a]
TCP + h_4-PhPyr	d_7-DMF/pentane	D_2O	0	70 (6.4:1)[a]
TCP + d_4-PhPyr	d_7-DMF/pentane	D_2O	80	7 (1.3:1)[a]
TCP + d_4-PhPyr	DMA/Et_2O	D_2O	34	10 (1.6:1)[a]
TCP + d_4-PhPyr	d_7-DMF/Et_2O	D_2O	63	10 (1.5:1)[a]
TCHep + h_4-PhPyr	DMA/n-Bu_2O	H_2O	0	82 (5:1)[b]
TCHep + h_4-PhPyr	DMA/*n*-Bu_2O	D_2O	21	79 (5:1)[b]
TCHep + d_4-PhPyr	DMA/*n*-Bu_2O	H_2O	38	19 (3.5:1)[b]
TCHep + d_4-PhPyr	DMA/*n*-Bu_2O	D_2O	49	10 (3.5:1)[b]
TCHep + d_4-PhPyr	d_7-DMF/*n*-Bu_2O	D_2O	74	8 (2:1)[b]

[a] Ratio BCP : BCP staffane. [b] Ratio BCHep : BCHep staffane.

Table S2. Deuterium-labelling studies.

7. Kinetic isotope effect studies

An approximate value for the kinetic isotope effect (KIE) for the reaction of [1.1.1]propellane **1** with amine **3a** was obtained according the following derivation:

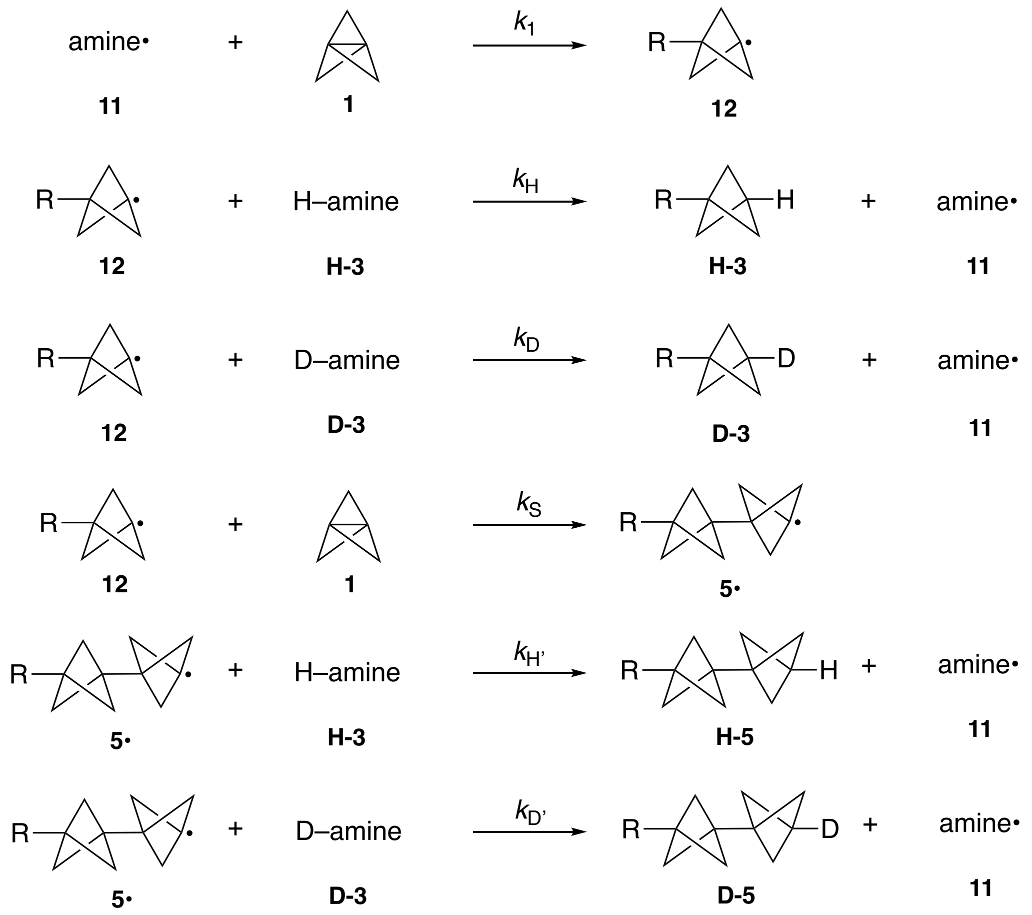

Rate of formation of **H/D-3**:

$$\frac{d[\mathbf{H/D-3}]}{dt} = \mathrm{k}_{H/D}[\mathbf{12}][\mathbf{H/D-3}]$$

Rate of formation of **H/D-5**:

$$\frac{d[\mathbf{H/D-5}]}{dt} = \mathrm{k}_{H'/D'}[\mathbf{5}\bullet][\mathbf{H/D-3}]$$

$$\frac{d[\mathbf{5}\bullet]}{dt} = \mathrm{k}_{S}[\mathbf{12}][\mathbf{1}] - \mathrm{k}_{H'/D'}[\mathbf{5}\bullet][\mathbf{H/D-3}]$$

$$\approx 0 \quad (steady\ state\ approx.)$$

$$\therefore \frac{d[\mathbf{H/D-5}]}{dt} \approx \mathrm{k}_{S}[\mathbf{12}][\mathbf{1}]$$

Relative product/staffane formation rate:

$$\frac{d[\mathbf{H/D-3}]}{dt} \Bigg/ \frac{d[\mathbf{H/D-5}]}{dt} = \frac{[\mathbf{H/D-3}]_t}{[\mathbf{H/D-5}]_t} = \frac{\mathrm{k}_{H/D}[\mathbf{H/D-3}]}{\mathrm{k}_S[\mathbf{1}]}$$

Kinetic isotope effect:

$$\frac{[\mathbf{H-3}]_t}{[\mathbf{H-5}]_t} \Bigg/ \frac{[\mathbf{D-3}]_t}{[\mathbf{D-5}]_t} = \frac{\mathrm{k}_H[\mathbf{H-3}]}{\mathrm{k}_D[\mathbf{D-3}]} \approx \frac{\mathrm{k}_H}{\mathrm{k}_D}$$

Assumptions of the model:

1. The rate of formation of radical **12** is independent of the deuteration state of amine **3**.
2. There is a large excess of **H/D-3** such that their respective concentrations at any given time remain approximately constant, and is equal across different reactions.
3. The concentration of staffane intermediate **5•** is constant and negligible (steady-state approximation).
4. The concentration of **1** is approximately equal in each of the reactions.

Using Entries 1 and 4 of Table 3, we obtain values of $\frac{[\mathbf{H-3}]_t}{[\mathbf{H-5}]_t}$ = 6.4 and $\frac{[\mathbf{D-3}]_t}{[\mathbf{D-5}]_t}$ = 1.3, resulting in $\frac{\mathrm{k}_H}{\mathrm{k}_D}$ = 4.9. Upper and lower bounds, given an uncertainty in the integrated NMR values of ±0.1, are 5.4 and 4.5, leading to an estimated KIE of **4.9 ± 0.5**.

8. Competition experiment: [1.1.1]Propellane vs [3.1.1]Propellane

In an NMR tube, 1-(4-fluorophenyl)pyrrolidine (80 mg, 0.5 mmol, 10.0 eq.) and 4CzIPN (1 mg, 1.0 μmol, 2.5 mol%) were added. The NMR tube was then evacuated and refilled with N_2 (g) for three cycles. Then [1.1.1]propellane solution (0.20 mL) (0.80 M in Et_2O, 0.06 mL, 0.05 mmol, 1.0 eq.), [3.1.1]propellane solution (0.35 M in *n*-Bu_2O, 0.1 mL, 0.05 mmol, 1.0 eq.), d_7-DMF (0.125 mL) and deionised H_2O (36 μl, 10 equiv.) were added under nitrogen atmosphere. Immediately, a proton-decoupled ^{19}F NMR spectrum was measured to record the t=0 composition of the reaction. Then, the NMR tube was expose to standard 440 nm blue LED irradiation conditions, and proton-decoupled ^{19}F NMR spectra were recorded at different time intervals (Table S3 / Figure S4), with integration of distinctive signals of the BCP/BCHep products. We assumed that the BCP peak obtained after 12 hours corresponds to 100% of conversion of the [1.1.1]propellane into BCP or degradation (albeit this does not allow for different rates of degradation of the two propellanes). Importantly, a consistent ratio of integrals was observed over the first 8 hours of the experiment (presumably before the concentration of the residual propellane becomes limiting / propellane degradation becomes influential).

Note: After 6 hours, the experiment was halted, and the NMR tubes were frozen at -20 ºC for 12 hours. No decomposition was observed during this period. Irradiation continued after this time.

Time / [h]	Relative [1.1.1]propellane conversion / [%]	Relative [3.1.1]propellane conversion / [%]	Ratio (BCP:BCHep)
0	0	0	–
0.5	19	5	**3.8**
1	28	7	**4.0**
2	35	9	**3.9**
4	46	12	**3.8**
6	56	14	**4.0**
8	71	18	**3.9**
10	87	24	3.6
12	100	30	3.3

Table S3. Relative conversion of propellanes, considering 100% as the conversion into BCP after 12 hours.

See graph below

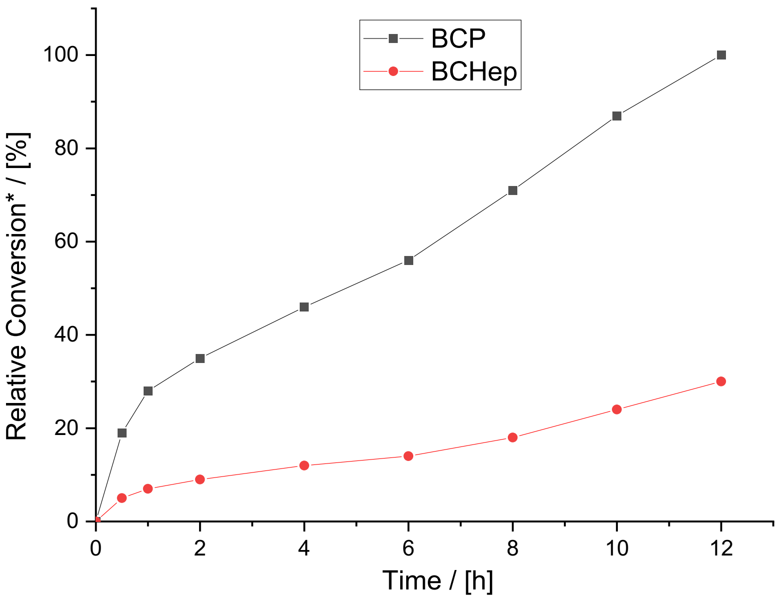

Fig. S4. Relative conversion of propellanes into considering 100% as the conversion into BCP after 12 hours*.

9. Photophysical experiments

Stern-Volmer quenching

Emission spectra were recorded at 20 ºC using an Edinburgh Instruments FS5 spectrofluorimeter, equipped with a xenon arc lamp (400 nm excitation), an SC-20 thermostatic sample holder, and a Hamamatsu R13456 PMT detector measuring at 410 – 800 nm. A quartz cuvette (10 mm path length) was charged with 2.5 mL of a 5 µM solution of photocatalyst 4CzIPN dissolved in DMA and was degassed by sparging with argon for 10 minutes. The appropriate volume of a 0.5 M solution (in DMA) of each quencher was added sequentially and the emission of the solution was measured.

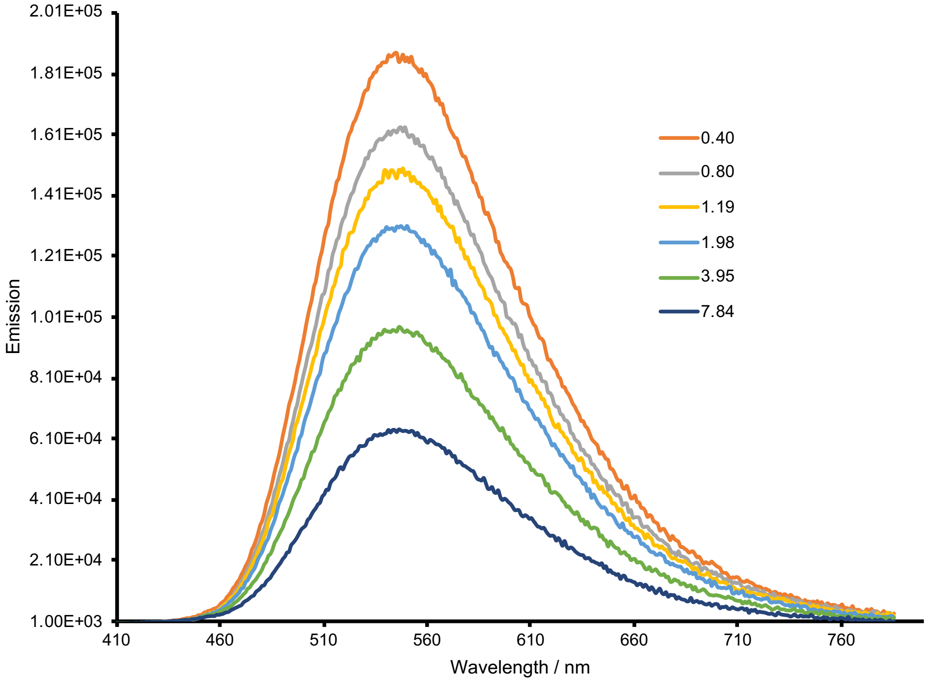

Figure S5. Fluorescence quenching titration for amine **3a** with a 5 µM solution of 4CzIPN in DMA.

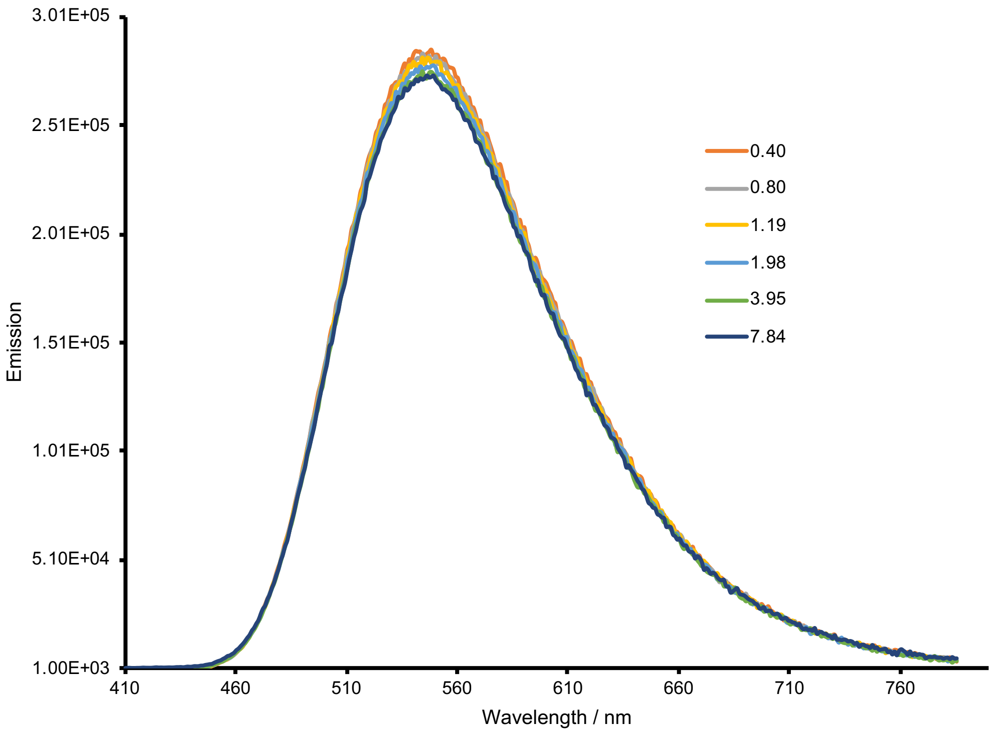

Figure S6. Fluorescence quenching titration for [1.1.1]propellane **1** with a 5 µM solution of 4CzIPN in DMA.

Table S4. Stern-Volmer quenching data for amine **3a** and [1.1.1]propellane **1** with a 5 µM solution of 4CzIPN in DMA.

	I_0/I (562 nm)	
[Q] / uM	amine **3a**	[1.1.1]propellane **1**
0.40	1.00	1.00
0.80	1.15	1.00
1.19	1.27	1.01
1.98	1.45	1.03
3.95	1.96	1.03
7.84	2.92	1.04

Reaction quantum yield measurement

Actinometry

The procedure was followed as written in Ref 1. Actinometry was carried out using an Evoluchem™ blue 18 W LED and Teflon™-capped quartz cuvette (path length = 1.000 cm, V = 2.35 mL). UV/vis absorption measurements were carried out using a Perkin Elmer Lambda20 spectrometer.

A 0.15 M solution of ferrioxalate was prepared by dissolving 2.21 g of potassium ferrioxalate hydrate in 30 mL of 0.05 M H_2SO_4, and was kept in the dark. A buffered solution of phenanthroline was prepared by dissolving 50 mg of phenanthroline and 11.25 g of sodium acetate in 50 mL of 0.5 M H_2SO_4. Each solution was sparged with N_2 for 10 min before use.

A cuvette was charged with 2.0 mL of ferrioxalate solution and irradiated for 45 seconds. 0.35 mL of phenanthroline buffer solution was subsequently added, and the solution was left to rest for 1 h. The absorbance of this solution (1.401 a.u.) was measured at 510 nm and compared with that of a non-irradiated sample (0.120 a.u.), resulting in ΔA = 1.281 a.u. and mol $Fe^{2+} = 2.71 \times 10^{-7}$ using Equation S1 ($\boldsymbol{\varepsilon}$ = 11000 $dm^3\ mol^{-1}\ cm^{-1}$)

$$\text{mol } Fe^{2+} = \frac{V \cdot \Delta A}{l \cdot \varepsilon} \qquad \text{(S1)}$$

The photon flux was calculated using Equation S2. The value of the fraction of light absorbed by the actinometer (f) was taken as 0.99877 from Ref. 1, and the quantum yield for the ferrioxalate actinometer (ϕ) was taken as 1.01 for a 0.15 M solution at 436 nm from Ref. 2.

$$\text{flux} = \frac{mol\, Fe^{2+}}{\phi \cdot t \cdot f} \qquad \text{(S2)}$$

The photon flux was calculated to be 5.97×10^{-9} einstein s^{-1}.

Substrate quantum yield measurement

A cuvette was charged with [1.1.1]propellane **1** (0.75 M in Et_2O, 0.27 mL, 0.2 mmol, 1 eq.), *N*-phenylpyrollidine **3a** (294 mg, 2.0 mmol, 10.0 eq.), deionised water (36 μl, 2.0 mmol, 10 equiv.), 4CzIPN (3.9 mg, 5.0 μmol, 2.5 mol%), and DMA (2.08 mL), and the resulting solution was irradiated using the actinometry setup described above for 3 h (10800 s). The yield of products **4a** and **5a** summed to 27%, resulting in 5.40×10^{-5} moles of product. The fraction of light absorbed by 4CzIPN at 436 nm was measured to be 0.998. Using Equation S3, the quantum yield of the reaction was calculated to be 0.84.

$$\phi = \frac{\text{mol products}}{\text{flux} \cdot \text{t} \cdot \text{f}} \qquad \text{(S3)}$$

10. Experimental Procedures

Tricyclo[1.1.1.0^{1,3}]pentane ([1.1.1]propellane)

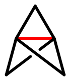

To a flame-dried round-bottom flask equipped with a stirrer bar was added 1,1-dibromo-2,2-bis(chloromethyl)cyclopropane (5.0 g, 16.9 mmol, 1.0 equiv.). The reaction vessel was evacuated and back-filled with argon three times, and then anhydrous Et_2O (10 mL) was added. The reaction vessel was cooled to −45 °C (dry ice / isopropanol bath). Phenyllithium (17.8 mL, 1.9 M in Bu_2O, 33.7 mmol, 2.0 equiv.) was added dropwise over 15 min at –45 °C, and the resulting mixture was stirred for 15 min at −45 °C. The cooling bath was replaced with an ice bath, and the reaction mixture was warmed to 0 °C, and then stirred at this temperature for 2 h. The mixture was then distilled at room temperature (10 mbar) using a rotary evaporator, the receiving flask of which was immersed in a dry ice / acetone bath. The TCP-containing distillate (12 mL, TCP concentration 0.8 M in Et_2O, 54%) was transferred to a flame-dried septum-sealed bottle under an inert atmosphere, and stored at −20 °C. The yield was determined by ^{1}H NMR spectroscopy with 1,2-dichloroethane as an internal standard.

Tricyclo[3.1.1.0^{1,5}]heptane ([3.1.1]propellane)

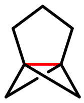

To a cooled (–78 °C) solution of 1,1-dibromo-2-(chloromethyl)-2-(3-chloropropyl)cyclopropane (9.74 g, 30.0 mmol, 1.0 equiv.) in anhydrous Et_2O (160 mL) was slowly added phenyllithium (31.8 mL, 60.4 mmol, 2.01 equiv., 1.9 M in *n*-Bu_2O). The resulting mixture was stirred at −78 °C for 15 minutes then warmed to room temperature and stirred for 7 h. The mixture was then distilled using a rotary evaporator (25 °C water bath temperature) equipped with a dry-ice cold finger condenser, with the receiving flask immersed in a dry ice / acetone bath. The Et_2O fraction was removed by slowly decreasing the applied pressure to 150 mbar. This fraction was then discarded. The remaining solution was distilled by slowly reducing the applied pressure to <10 mbar to afford a solution of [3.1.1]propellane 1 in *n*-Bu_2O which was stored under an inert atmosphere at −20 °C. The yield was determined by ^{1}H NMR spectroscopy using 1,2-dichloroethane as an internal standard. The concentration of the [3.1.1]propellane solution ranged between 0.25 M and 0.50 M, with yields of 43-61%.

a) Synthesis of Bicyclo[1.1.1.]pentanes

2-(Bicyclo[1.1.1]pentan-1-yl)-1-phenylpyrrolidine, 3a and 4a

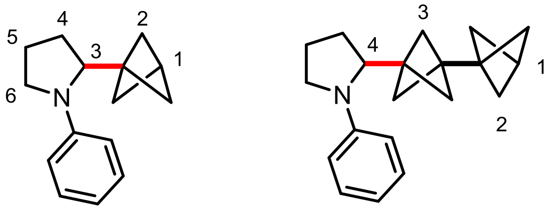

N-phenylpyrollidine (294 mg, 2.0 mmol, 10.0 eq.), 4CzIPN (3.9 mg, 5.0 μmol, 2.5 mol%), deionised H_2O (36 μl, 10 equiv.) and [1.1.1]propellane (0.80 M in Et_2O, 0.25 mL, 0.2 mmol, 1.0 eq.) in DMA (0.4 mL) were submitted to General Procedure 1A for 24 h. Purification by column chromatography (SiO_2, pentane) gave **3a** and **4a** (31 mg of a 6:1 ratio of **3a**:**4a**, 80%) as a colourless oil.

R_f = 0.16 (pentane).

^{1}H NMR (400 MHz, $CDCl_3$) δ 7.23 – 7.15 (2H, m, ArH), 6.67 – 6.59 (3H, m, ArH), 3.73 (1H, dd, *J* = 7.9, 1.3 Hz, H3), 3.51 – 3.43 (1H, m, H6), 3.15 – 3.06 (1H, m, H6), 2.45 (1H, s, H1), 2.07 – 1.77 (4H, m, H2, H3), 1.75 – 1.68 (6H, m, H2).

^{13}C NMR (101 MHz, $CDCl_3$) δ 148.7, 129.0, 115.4, 112.0, 58.9, 49.8, 49.0, 48.2, 28.6, 27.3, 24.0.

HRMS (ESI) calc. for $C_{15}H_{20}N$ $[M+H]^+$ 214.1590, found 214.1591.

IR (film, ν_{max}/cm^{-1}) 3659, 2979, 2904, 2886, 2870, 1596, 1533, 1360, 1157, 953.

Characteristic peaks for staffane product:

^{1}H NMR (400 MHz, $CDCl_3$) δ 3.77 (1H, dd, *J* = 7.4, 1.9 Hz, H4), 2.37 (1H, s, H1), 1.58 (6H, s, H2), 1.54 – 1.47 (6H, m, H3).

HRMS (ESI^+) calc. for $C_{20}H_{26}N$ $[M+H]^+$ 280.2060, found 280.2061.

2-(Bicyclo[1.1.1]pentan-1-yl)-1-(4-(methylsulfonyl)phenyl)pyrrolidine, 3b

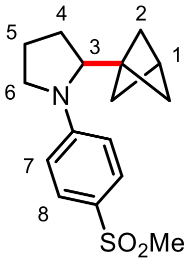

1-(4-(Methylsulfonyl)phenyl)pyrrolidine (450 mg, 2.0 mmol, 10.0 eq.), 4CzIPN (3.9 mg, 5.0 µmol, 2.5 mol%), deionised H_2O (36 µl, 10 equiv.) and [1.1.1]propellane (0.80 M in Et_2O, 0.25 mL, 0.2 mmol, 1.0 eq.) in DMA (2.0 mL) were submitted to General Procedure 1A for 48 h. Purification by column chromatography (SiO_2, pentane / ethyl acetate, 7:3→1:1) gave **3b** (36 mg, 64%) as a white solid.

R_f = 0.41 (pentane / ethyl acetate, 1:1).
m.p. = 130–132 °C ($CDCl_3$).
^{1}H NMR (400 MHz, $CDCl_3$) 7.68 (2H, d, *J* = 9.1 Hz, H8), 6.63 (2H, d, *J* = 9.1 Hz, H7), 3.85 (1H, dd, *J* = 7.8, 1.3 Hz, H3), 3.49 (1H, ddd, *J* = 9.9, 7.6, 2.8 Hz, H6), 3.19 (1H, td, *J* = 9.5, 7.6 Hz, H6), 3.00 (3H, s, Me), 2.46 (1H, s, H1), 2.12 – 1.79 (4H, m, H4, H5), 1.77 – 1.66 (6H, m, H2).
^{13}C NMR (101 MHz, $CDCl_3$) δ 151.6, 129.0, 125.4, 111.6, 58.9, 50.0, 48.6, 47.5, 45.3, 28.6, 27.4, 23.7.
HRMS (ESI^+) calc. for $C_{16}H_{22}NO_2S$ $[M+H]^+$ 292.1366, found 292.1366.
IR (film, ν_{max}/cm^{-1}) 2961, 2906, 2865, 1592, 1290, 1139.

Methyl-4-(2-(bicyclo[1.1.1]pentan-1-yl)pyrrolidin-1-yl)benzoate, 3c

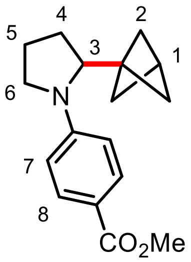

Methyl 4-(pyrrolidin-1-yl)benzoate (410 mg, 2.0 mmol, 10.0 eq.), 4CzIPN (3.9 mg, 5.0 µmol, 2.5 mol%), deionised H_2O (36 µl, 10 equiv.) and [1.1.1]propellane (0.80 M in Et_2O, 0.25 mL, 0.2 mmol, 1.0 eq.) in DMA (2.0 mL) were submitted to General Procedure 1A for 48 h. Purification by column chromatography (DCM / pentane / ethyl acetate, 5:5:0.2) gave **3c** (27 mg, 52%) as a colourless oil.

$\mathbf{R_f}$ = 0.49 (DCM / pentane / ethyl acetate, 5:5:0.4).

^{1}H NMR (400 MHz, $CDCl_3$) 7.86 (2H, d, J = 9.0 Hz, H8), 6.57 (2H, d, J = 9.0 Hz, H7), 3.90 – 3.81 (4H, m, H3 and OMe), 3.49 (1H, ddd, J = 9.8, 7.7, 2.6 Hz, H6), 3.19 (1H, td, J = 9.6, 7.5 Hz, H6), 2.45 (1H, s, H1), 2.11 – 1.78 (4H, m, H4, H5), 1.77 – 1.66 (6H, m, H2).

^{13}C NMR (101 MHz, $CDCl_3$) δ 167.7, 151.5, 131.2, 116.4, 111.2, 58.7, 51.5, 50.0, 48.5, 47.7, 28.6, 27.3, 23.7.

HRMS (ESI$^+$) calc. for C17H22NO2 $[M+H]^+$ 272.1645, found 272.1645.

IR (film, ν_{max}/cm^{-1}) 2966, 2906, 2870, 1701, 1562, 1313, 1195, 720.

4-(2-(Bicyclo[1.1.1]pentan-1-yl)pyrrolidin-1-yl)benzonitrile, 3d and 4d

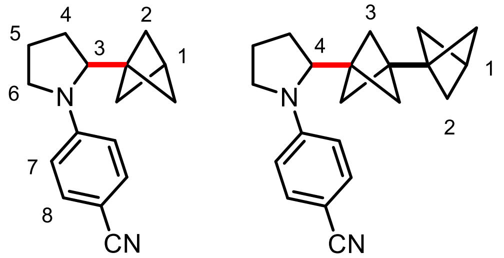

4-(Pyrrolidin-1-yl)benzonitrile (344 mg, 2.0 mmol, 10.0 eq.), 4CzIPN (3.9 mg, 5.0 µmol, 2.5 mol%), deionised H_2O (36 µl, 10 equiv.) and [1.1.1]propellane (0.80 M in Et_2O, 0.25 mL, 0.2 mmol, 1.0 eq.) in DMA (0.4 mL) were submitted to General Procedure 1A. Purification by column chromatography (SiO_2, pentane / Et_2O, 95:5) gave **3d** and **4d** (31 mg of a 6:1 ratio of **3d**:**4d**, 72%) as a colourless oil.

$\mathbf{R_f}$ = 0.31 (pentane / Et_2O, 9:1).

^{1}H NMR (400 MHz, $CDCl_3$) δ 7.44 – 7.40 (2H, m, H8), 6.61 – 6.55 (2H, m, H7), 3.84 – 3.81 (1H, m, H3), 3.47 (1H, ddd, *J* = 9.8, 7.7, 2.6 Hz, H6), 3.17 (1H, ddd, *J* = 9.8, 9.5, 7.7 Hz, H6), 2.47 (1H, s, H1), 2.08 – 1.83 (4H, m, H4, H5), 1.75 – 1.67 (6H, m, H2).

^{13}C NMR (101 MHz, $CDCl_3$) δ 150.7, 133.3, 121.1, 112.0, 96.7, 58.8, 50.0, 48.5, 47.4, 28.5, 27.4, 23.6.

HRMS (ESI$^+$) calc. for $C_{16}H_{19}N_2$ $[M+H]^+$ 239.1543, found 239.1544.

IR (film, ν_{max}/cm^{-1}) 2963, 2906, 2870, 2211, 1605, 1519, 1382, 1205, 1177, 816.

Characteristic peaks for staffane product:

^{1}H NMR (400 MHz, $CDCl_3$) δ 3.86 (1H, dd, *J* = 7.3, 2.0 Hz, H4), 2.37 (1H, s, H1), 1.58 (6H, s, H2), 1.54 – 1.46 (6H, m, H3).

HRMS (ESI$^+$) calc. for $C_{21}H_{24}N_2Na$ $[M+Na]^+$ 327.1832, found 327.1833.

2-(Bicyclo[1.1.1]pentan-1-yl)-1-(4-fluorophenyl)pyrrolidine, 3e and 4e

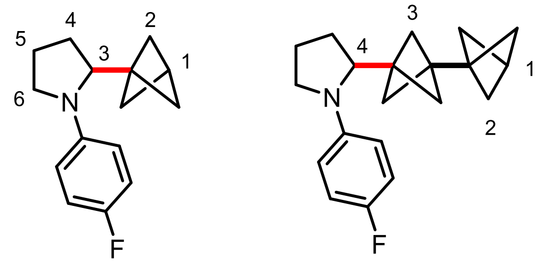

1-(4-Fluorophenyl)pyrrolidine (330 mg, 2.0 mmol, 10.0 eq.), 4CzIPN (3.9 mg, 5.0 µmol, 2.5 mol%), deionised H_2O (36 µl, 10 equiv.) and [1.1.1]propellane (0.80 M in Et_2O, 0.25 mL, 0.2 mmol, 1.0 eq.) in DMA (0.4 mL) were submitted to General Procedure 1A. Purification by column chromatography (SiO_2, pentane) gave **3e** and **4e** (30 mg of a 6:1 ratio of **3e**:**4e**, 72%) as a colourless oil.

$\mathbf{R_f}$ = 0.31 (pentane / Et_2O, 50:1).

^{1}H NMR (400 MHz, $CDCl_3$) δ 6.95 – 6.86 (2H, m, ArH), 6.56 – 6.50 (2H, m, ArH), 3.65 (1H, dd, J = 7.9, 1.5 Hz, H3), 3.44 (1H, ddd, J = 9.0, 7.5, 2.2 Hz, H6), 3.05 (1H, ddd, J = 9.2, 9.0, 7.0 Hz, H6), 2.46 (1H, s, H1), 2.06 – 1.76 (4H, m, H4, H5), 1.75 – 1.65 (6H, m, H6).

^{13}C NMR (101 MHz, $CDCl_3$) δ 154.8 (d, $^1J_{CF}$ = 232.9 Hz), 145.4, 115.3 (d, $^2J_{CF}$ = 21.9 Hz), 112.3 (d, $^3J_{CF}$ = 7.1 Hz), 59.3, 49.8, 49.5, 48.2, 28.7, 27.4, 24.2.

^{19}F NMR (377 MHz, $CDCl_3$) δ –131.3.

HRMS (ESI$^+$) calc. for $C_{15}H_{19}FN$ $[M+H]^+$ 232.1496, found 232.1497.

IR (film, ν_{max}/cm^{-1}) 2963, 2907, 2869, 1516, 1361, 1343, 1226, 1204, 811.

Characteristic peaks for staffane product:

^{1}H NMR (400 MHz, $CDCl_3$) δ 3.70 (1H, dd, J = 6.8, 2.6 Hz, H4), 2.37 (1H, s, H1), 1.59 (6H, s, H2), 1.53 – 1.45 (6H, m, H3).

HRMS (ESI$^+$) calc. for $C_{20}H_{25}FN$ $[M+H]^+$ 298.1966, found 298.1966.

2-(Bicyclo[1.1.1]pentan-1-yl)-1-(4-chlorophenyl)pyrrolidine, 3f and 4f

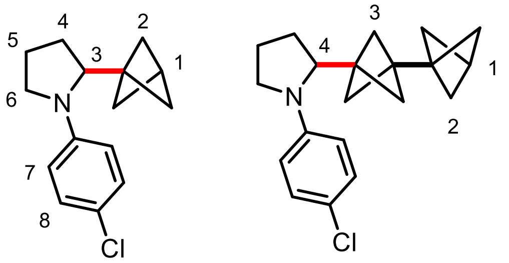

1-(4-Chlorophenyl)pyrrolidine (362 mg, 2.0 mmol, 10.0 eq.), 4CzIPN (3.9 mg, 5.0 µmol, 2.5 mol%), deionised H_2O (36 µl, 10 equiv.) and [1.1.1]propellane (0.80 M in Et_2O, 0.25 mL, 0.2 mmol, 1.0 eq.) in DMA (1.0 mL) were submitted to General Procedure 1A. Purification by column chromatography (SiO_2, pentane) gave **3f** and **4f** (26 mg of a 6:1 ratio of **3f**:**4f**, 58%) as a colourless oil.

R_f = 0.43 (pentane / Et_2O, 50:1);

^{1}H NMR (400 MHz, $CDCl_3$) δ 7.15 – 7.09 (2H, m, ArH), 6.56 – 6.49 (2H, m, ArH), 3.69 (1H, dd, *J* = 7.8, 1.4 Hz, H4), 3.43 (1H, ddd, *J* = 9.3, 7.5, 2.4 Hz, H6), 3.07 (1H, ddd, *J* = 9.3, 9.2, 7.1 Hz, H6), 2.46 (1H, s, H1), 2.05 – 1.77 (4H, m, H4, H5), 1.75 – 1.66 (6H, m, H2).

^{13}C NMR (101 MHz, $CDCl_3$) δ 147.1, 128.7, 120.1, 113.0, 59.0, 49.8, 49.0, 48.0, 28.7, 27.4, 24.0.

HRMS (ESI$^+$) calc. for $C_{15}H_{19}ClN$ $[M+H]^+$ 248.1201, found 248.1201.

IR (film, ν_{max}/cm^{-1}) 2963, 2906, 2869, 1598, 1497, 1361, 1204, 1184, 1158, 1096, 807.

Characteristic peaks for staffane product:

^{1}H NMR (400 MHz, $CDCl_3$) δ 3.73 (1H, dd, *J* = 6.9, 2.4 Hz, H4), 2.37 (1H, s, H1), 1.59 (6H, s, H2), 1.53 – 1.45 (6H, m, H3).

HRMS (ESI$^+$) calc. for $C_{20}H_{25}ClN$ $[M+H]^+$ 314.1670, found 314.1670.

3-(2-(Bicyclo[1.1.1]pentan-1-yl)pyrrolidin-1-yl)benzonitrile, 3g

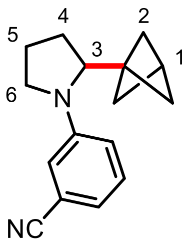

3-(pyrrolidin-1-yl)benzonitrile (344 mg, 2.0 mmol, 10.0 eq.), 4CzIPN (3.9 mg, 5.0 µmol, 2.5 mol%), deionised H_2O (36 µl, 10 equiv.) and [1.1.1]propellane (0.80 M in Et_2O, 0.25 mL, 0.2 mmol, 1.0 eq.) in DMA (1.0 mL) were submitted to General Procedure 1A. Purification by column chromatography (SiO_2, pentane / DCM / EtOAc, 90:7.5:2.5) gave **3g** (21 mg, 46%) as a colourless oil.

R_f = 0.65 (pentane / DCM / EtOAc, 80:15:5).

^{1}H NMR (400 MHz, $CDCl_3$) δ 7.23 – 7.16 (1H, m, ArH), 6.88 (1H, dt, J = 7.4, 1.2 Hz, ArH), 6.82 – 6.77 (2H, m, ArH), 3.73 (1H, d, J = 7.8 Hz, H3), 3.44 (1H, ddd, J = 9.4, 7.4, 2.7 Hz, H6), 3.10 (1H, td, J = 9.4, 7.4 Hz, H6), 2.47 (1H, s, H1), 2.09 – 1.76 (4H, m, H4, H5), 1.76 – 1.67 (6H, m, H2).

^{13}C NMR (101 MHz, $CDCl_3$) δ 148.3, 129.6, 120.1, 118.7, 116.2, 114.8, 112.7, 58.9, 49.9, 48.7, 48.7, 47.6, 28.6, 27.4, 23.8.

HRMS (ESI$^+$) calc. for $C_{16}H_{19}N_2$ $[M+H]^+$ 239.1543, found 239.1543.

IR (film, ν_{max}/cm^{-1}) 2964, 2869, 2227, 1596, 1494, 1368.

2-(2-(Bicyclo[1.1.1]pentan-1-yl)pyrrolidin-1-yl)benzonitrile, 3h and 4h

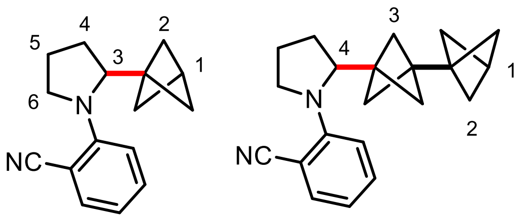

2-(Pyrrolidin-1-yl)benzonitrile (344 mg, 2.0 mmol, 10.0 eq.), 4CzIPN (3.9 mg, 5.0 µmol, 2.5 mol%), deionised H_2O (36 µl, 10 equiv.) and [1.1.1]propellane (0.80 M in Et_2O, 0.25 mL, 0.2 mmol, 1.0 eq.) in DMA (0.4 mL) were submitted to General Procedure 1A. Purification by column chromatography (SiO_2, pentane / EtOAc, 95:5) gave **3h** and **4h** (15 mg of a 5:1 ratio of **3h**:**4h**, 35%) as a colourless oil.

R_f = 0.40 (pentane / EtOAc, 9:1).

^{1}H NMR (400 MHz, $CDCl_3$) δ 7.44 – 7.40 (1H, m, ArH), 7.31 – 7.26 (1H, m, ArH), 6.80 – 6.77 (1H, m, ArH), 6.68 – 6.62 (1H, m, ArH), 4.27 (1H, dd, J = 6.9, 4.2 Hz, H3), 3.99 – 3.91 (1H, m, H6), 3.51 – 3.41 (1H, m, H6), 2.42 (1H, s, H1), 2.07 – 1.79 (4H, m, H4, H5), 1.75 – 1.64 (6H, m, H2).

^{13}C NMR (101 MHz, $CDCl_3$) δ 150.9, 135.8, 133.0, 121.6, 116.5, 115.9, 96.2, 58.5, 51.5, 49.7, 47.4, 28.4, 27.5, 24.3.

HRMS (ESI$^+$) calc. for $C_{16}H_{19}N_2$ $[M+H]^+$ 239.1543, found 239.1543.

IR (film, ν_{max}/cm^{-1}) 2965, 2870, 2208, 1600, 1492, 1446, 746.

Characteristic peaks for staffane product:

^{1}H NMR (400 MHz, $CDCl_3$) δ 3.33 – 3.30 (1H, m, H4), 2.35 (1H, s, H1), 1.56 (6H, s, H2), 1.50 – 1.42 (6H, m, H3).

HRMS (ESI$^+$) calc. for $C_{21}H_{24}N_2Na$ $[M+Na]^+$ 327.1832, found 327.1833.

1-(4-(2-(Bicyclo[1.1.1]pentan-1-yl)pyrrolidin-1-yl)-2,5-difluorophenyl)ethan-1-one, 3i

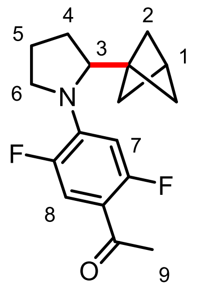

1-(2,5-Difluoro-4-(pyrrolidin-1-yl)phenyl)ethan-1-one (450 mg, 2.0 mmol, 10.0 eq.), 4CzIPN (3.9 mg, 5.0 μmol, 2.5 mol%), deionised H_2O (36 μl, 10 equiv.) and [1.1.1]propellane (0.80 M in Et_2O, 0.25 mL, 0.2 mmol, 1.0 eq.) in DMA (1.0 mL) were submitted to General Procedure 1A. Purification by column chromatography (SiO_2, pentane / EtOAc, 98:2 → 95:5) gave **3i** (32 mg, 60%) as a yellow solid.

$\mathbf{R_f}$ = 0.42 (pentane / EtOAc, 95:5).

m.p. = 70–72 °C ($CDCl_3$).
^{1}H NMR (400 MHz, $CDCl_3$) δ 7.49 (1H, dd, J = 15.3, 7.0 Hz, H8), 6.27 (1H, dd, J = 14.2, 7.0 Hz, H7), 4.24 – 4.14 (1H, m, H3), 3.66 – 3.55 (1H, m, H6), 3.40 – 3.29 (1H, m, H6), 2.53 (3H, d, J = 5.2 Hz, H9), 2.42 (1H, s, H1), 2.08 – 1.85 (4H, m, H4, H5), 1.72 – 1.64 (6H, m, H2).

^{13}C NMR (101 MHz, $CDCl_3$) δ 193.2 (d, J = 4.2 Hz), 160.2 (d, J = 249.2 Hz), 147.2 (d, J = 239.2 Hz), 142.3 (m), 116.8 (dd, J = 25.1, 5.5 Hz), 112.6 (d, J = 9.8 Hz), 101.8 (dd, J = 31.8, 5.6 Hz), 59.7 (d, J = 7.1 Hz), 50.2 (d, J = 4.9 Hz), 49.8, 47.9, 30.9 (d, J = 8.1 Hz), 28.4, 27.2, 23.2 (d, J = 2.2 Hz).

^{19}F NMR (377 MHz, $CDCl_3$) δ –111.80 (m), –131.20 (m).

HRMS (ESI$^+$) calc. for $C_{17}H_{19}F_2NO$ $[M+H]^+$ 292.1507, found 292.1507.

IR (film, ν_{max}/cm^{-1}) 2980, 2906, 2869, 1617, 1365.

Characteristic peaks for staffane product:

^{1}H NMR (400 MHz, $CDCl_3$) δ 1.55 (6H, s, H2), 1.48 – 1.41 (6H, m, H3).

HRMS (ESI$^+$) calc. for $C_{22}H_{25}F_2NO$ $[M+H]^+$ 358.1977, found 358.1977.

2-(2-(Bicyclo[1.1.1]pentan-1-yl)pyrrolidin-1-yl)pyridine, 3j and 4j

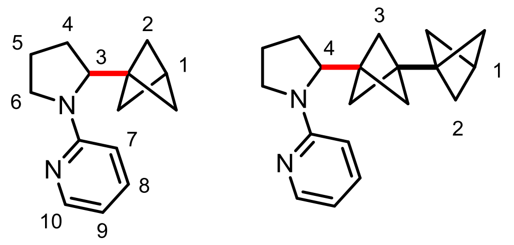

2-(Pyrrolidin-1-yl)pyridine (296 mg, 2.0 mmol, 10.0 eq.), 4CzIPN (3.9 mg, 5.0 µmol, 2.5 mol%), deionised H_2O (36 µl, 10 equiv.) and [1.1.1]propellane (0.80 M in Et_2O, 0.25 mL, 0.2 mmol, 1.0 eq.) in DMA (0.4 mL) were submitted to General Procedure 1A. Purification by column chromatography (SiO_2, pentane / EtOAc, 95:5) gave **3j** and **4j** (28 mg of a 12:1 ratio of **3j**:**4j**, 70%) as a colourless oil.

R_f = 0.52 (pentane / EtOAc, 8:2).

^{1}H NMR (400 MHz, $CDCl_3$) δ 8.12 (1H, ddd, *J* = 5.0, 2.0, 1.0 Hz, H10), 7.37 (1H, ddd, *J* = 8.8, 7.1, 2.0 Hz, H8), 6.48 (1H, ddd, *J* = 7.1, 5.0, 1.0 Hz, H9), 6.42 (1H, dt, *J* = 8.8, 1.0 Hz, H7), 3.97 (1H, dd, *J* = 7.4, 1.8 Hz, H3), 3.59 (1H, ddd, *J* = 10.4, 7.7, 2.3 Hz, H6), 3.39 (1H, td, *J* = 10.0, 9.6, 7.2 Hz, H6), 2.43 (1H, s, H1), 2.05 – 1.80 (4H, m, H4, H5), 1.76 – 1.64 (6H, m, H2).

^{13}C NMR (101 MHz, $CDCl_3$) δ 158.0, 148.1, 136.6, 111.2, 107.1, 57.7, 49.8, 47.8, 47.7, 28.5, 27.3, 23.7.

HRMS (ESI$^+$) calc. for $C_{14}H_{19}N_2$ [M+H]$^+$ 215.1543, found 215.1543.

IR (film, ν_{max}/cm^{-1}) 2963, 2906, 2868, 1596, 1477, 1438.

Characteristic peaks for staffane product:

^{1}H NMR (400 MHz, $CDCl_3$) δ 2.36 (1H, s, H1), 1.58 (6H, s, H2), 1.52 – 1.45 (6H, m, H3).

3-(2-(Bicyclo[1.1.1]pentan-1-yl)pyrrolidin-1-yl)pyridine, 3k

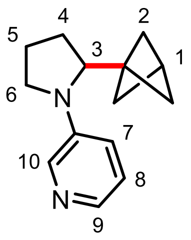

3-(Pyrrolidin-1-yl)pyridine (296 mg, 2.0 mmol, 10.0 eq.), 4CzIPN (3.9 mg, 5.0 μmol, 2.5 mol%), deionised H_2O (36 μl, 10 equiv.) and [1.1.1]propellane (0.80 M in Et_2O, 0.25 mL, 0.2 mmol, 1.0 eq.) in DMA (0.4 mL) were submitted to General Procedure 1A. Purification by column chromatography (SiO_2, pentane / EtOAc, 3:1) gave **3k** (27 mg, 63%) as a colourless oil.

R_f = 0.44 (pentane / EtOAc, 1:1).

^{1}H NMR (400 MHz, $CDCl_3$) δ 8.03 (1H, d, *J* = 1.8 Hz, H10), 7.89 (1H, d, *J* = 4.6 Hz, H9), 7.06 (1H, dd, *J* = 8.4, 4.6 Hz, H8), 6.85 (1H, ddd, *J* = 8.4, 3.0, 1.8 Hz, H7), 3.75 (1H, d, *J* = 7.9 Hz, H3), 3.52 – 3.41 (1H, m, H6), 3.10 (1H, td, *J* = 9.1, 7.4 Hz, H6), 2.45 (s, 1H), 2.07 – 1.79 (4H, m, H4, H5), 1.75 – 1.64 (6H, m, H2).

^{13}C NMR (101 MHz, $CDCl_3$) δ 144.3, 136.9, 134.7, 123.4, 118.1, 58.6, 49.8, 48.5, 47.7, 28.6, 27.4, 23.8.

HRMS (ESI$^+$) calc. for $C_{14}H_{19}N_2$ $[M+H]^+$ 215.1543, found 215.1543.

IR (film, ν_{max}/cm^{-1}) 3038, 2963, 1583, 1490, 1361.

4-(2-(Bicyclo[1.1.1]pentan-1-yl)pyrrolidin-1-yl)pyridine, 3l

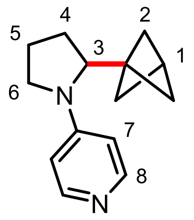

4-(Pyrrolidin-1-yl)pyridine (296 mg, 2.0 mmol, 10.0 eq.), 4CzIPN (3.9 mg, 5.0 μmol, 2.5 mol%), deionised H_2O (36 μl, 10 equiv.) and [1.1.1]propellane (0.80 M in Et_2O, 0.25 mL, 0.2 mmol, 1.0 eq.) in DMA (0.4 mL) were submitted to General Procedure 1A. Purification by column chromatography (SiO_2, CH_2Cl_2 / MeOH / 30% NH_3OH, 9:1:0.1) gave **3l** (25 mg, 65%) as a colourless oil.

R_f = 0.33 (CH_2Cl_2 / MeOH / 30% NH_3OH, 9:1:0.1).

^{1}H NMR (400 MHz, $CDCl_3$) δ 8.19 – 8.11 (2H, m, H8), 6.47 – 6.39 (2H, m, H7), 3.81 (1H, d, J = 7.7 Hz, H3), 3.45 (1H, ddd, J = 9.9, 7.5, 2.8 Hz, H6), 3.17 (1H, td, J = 9.5, 7.7 Hz, H6), 2.47 (1H, s, H1), 2.10 – 1.78 (4H, m, H4, H5), 1.77 – 1.66 (6H, m, H2).

^{13}C NMR (101 MHz, $CDCl_3$) δ 152.6, 148.9, 107.5, 58.5, 50.0, 48.0, 47.4, 28.5, 27.4, 23.5.

HRMS (ESI$^+$) calc. for $C_{14}H_{19}N_2$ $[M+H]^+$ 215.1543, found 215.1543.

IR (film, ν_{max}/cm^{-1}) 2963, 2906, 2869, 1563, 1511, 1388.

6-(2-(Bicyclo[1.1.1]pentan-1-yl)pyrrolidin-1-yl)quinoline, 3m

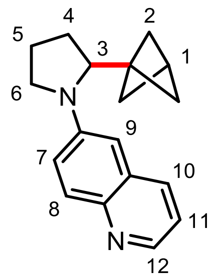

6-(Pyrrolidin-1-yl)quinoline (397 mg, 2.0 mmol, 10.0 eq.), 4CzIPN (3.9 mg, 5.0 µmol, 2.5 mol%), deionised H_2O (36 µl, 10 equiv.) and [1.1.1]propellane (0.80 M in Et_2O, 0.25 mL, 0.2 mmol, 1.0 eq.) in DMA (2.0 mL) were submitted to General Procedure 1A. Purification by column chromatography (SiO_2, pentane / EtOAc, 3:1) gave **3m** (32 mg, 61%) as a yellow oil.

R_f = 0.55 (pentane / EtOAc, 1:1).

^{1}H NMR (400 MHz, $CDCl_3$) δ 8.56 (1H, dd, *J* = 4.2, 1.7 Hz, H12), 7.94 – 7.86 (2H, m, ArH), 7.27 (1H, dd, *J* = 9.2, 2.9 Hz, ArH), 7.22 (1H, dd, *J* = 8.3, 4.2 Hz, H11), 6.67 (1H, d, *J* = 2.8 Hz, ArH), 3.92 (1H, dd, *J* = 7.6, 1.6 Hz, H3), 3.57 (1H, ddd, *J* = 9.0, 7.9, 2.3 Hz, H6), 3.25 (1H, td, *J* = 9.3, 7.3 Hz, H6), 2.46 (1H, s, H1), 2.14 – 1.83 (4H, m, H4, H5), 1.80 – 1.68 (6H, m, H2).

^{13}C NMR (101 MHz, $CDCl_3$) δ 146.4, 145.6, 142.2, 133.7, 130.2, 129.8, 121.3, 119.6, 103.8, 58.8, 50.0, 48.9, 48.0, 28.7, 27.4, 24.0.

HRMS (ESI$^+$) calc. for $C_{18}H_{21}N_2$ $[M+H]^+$ 265.1700, found 265.1699.

IR (film, ν_{max}/cm^{-1}) 2964, 2869, 1617, 1506, 1378.

2-(Bicyclo[1.1.1]pentan-1-yl)-1-phenylpiperidine, 3n and 4n

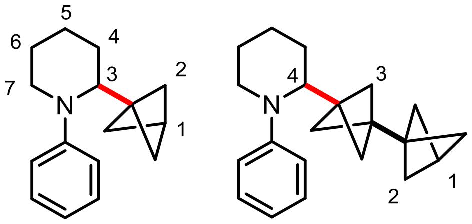

1-phenylpiperidine (322 mg, 2.0 mmol, 10.0 eq.), 4CzIPN (3.9 mg, 5.0 µmol, 2.5 mol%), deionised H_2O (36 µl, 10 equiv.) and [1.1.1]propellane (0.80 M in Et_2O, 0.25 mL, 0.2 mmol, 1.0 eq.) in DMA (0.4 mL) were submitted to General Procedure 1A. Purification by column chromatography (SiO_2, pentane / EtOAc, 99:1) gave **3n** and **4n** (21 mg of a 4:1 ratio of **3n**:**4n**, 52%) as a colourless oil.

R_f = 0.23 (pentane).

^{1}H NMR (400 MHz, $CDCl_3$) δ 7.24 – 7.14 (2H, m, ArH), 6.98 – 6.86 (2H, m, ArH), 6.78 – 6.69 (1H, m, ArH), 3.86 – 3.78 (1H, m, H3), 3.40 – 3.26 (1H, m, H7), 3.20 – 3.06 (1H, m, H7), 2.33 (1H, s, H1), 1.82 – 1.55 (12H, m, H2, H4, H5, H6).

^{13}C NMR (101 MHz, $CDCl_3$) δ 152.3, 128.9, 118.3, 116.5, 55.6, 52.3, 48.6, 45.2, 28.0, 27.4, 25.7, 20.5.

HRMS (ESI$^+$) calc. for $C_{16}H_{22}N$ $[M+H]^+$ 228.1747, found 228.1748.

IR (film, ν_{max}/cm^{-1}) 2961, 2867, 1596, 1495, 1252.

Characteristic peaks for staffane product:

^{1}H NMR (400 MHz, $CDCl_3$) δ 3.89 – 3.87 (1H, m, H4), 2.32 (1H, s, H1), 1.53 (6H, s, H2), 1.47 – 1.41 (6H, m, H3).

HRMS (ESI$^+$) calc. for $C_{21}H_{28}N$ $[M+H]^+$ 294.2216, found 294.2217.

2-(Bicyclo[1.1.1]pentan-1-yl)-1-phenylazepane, 3o and 4o

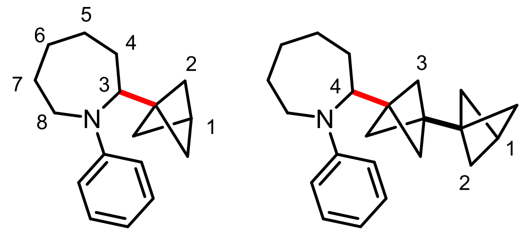

1-phenylazepane (350 mg, 2.0 mmol, 10.0 eq.), 4CzIPN (3.9 mg, 5.0 µmol, 2.5 mol%), deionised H_2O (36 µl, 10 equiv.) and [1.1.1]propellane (0.80 M in Et_2O, 0.25 mL, 0.2 mmol, 1.0 eq.) in DMA (0.4 mL) were submitted to General Procedure 1A. Purification by column chromatography (SiO_2, pentane) gave **3o** and **4o** (20 mg of a 5:1 ratio of **3o**:**4o**, 47%) as a colourless oil.

$\mathbf{R_f}$ = 0.77 (pentane / Et_2O, 99:1).

^{1}H NMR (400 MHz, $CDCl_3$) δ 7.25 – 7.17 (2H, m, ArH), 6.74 – 6.67 (2H, m, ArH), 6.63 – 6.55 (1H, m, ArH), 3.67 (1H, dd, *J* = 11.6, 6.2 Hz, H3), 3.56 – 3.44 (1H, m, H8), 3.22 – 3.07 (1H, m, H8), 2.46 (1H, s, H1), 2.06 – 1.92 (1H, m, H4), 1.84 – 1.47 (11H, m, H2), 1.33 – 1.14 (2H, m).

^{13}C NMR (101 MHz, $CDCl_3$) δ 149.3, 129.2, 114.4, 110.5, 56.6, 49.6, 47.7, 43.7, 32.0, 30.2, 27.8, 27.1, 25.3.

HRMS (ESI^+) calc. for $C_{17}H_{24}N$ $[M+H]^+$ 242.1903, found 242.1903.

IR (film, ν_{max}/cm^{-1}) 2960, 2924, 2904, 2867, 1595, 1503, 1198.

Characteristic peaks for staffane product:

^{1}H NMR (400 MHz, $CDCl_3$) δ 2.37 (1H, s, H1), 1.58 (6H, s, H2).
HRMS (ESI^+) calc. for $C_{22}H_{30}N$ $[M+H]^+$ 308.2373, found 308.2373.

2-(Bicyclo[1.1.1]pentan-1-yl)-1-(pyridin-3-yl)azepane, 3p

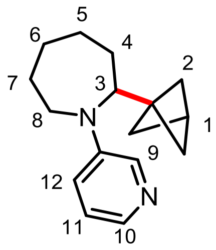

1-phenylazepane (350 mg, 2.0 mmol, 10.0 eq.), 4CzIPN (3.9 mg, 5.0 µmol, 2.5 mol%), deionised H_2O (36 µl, 10 equiv.) and [1.1.1]propellane (0.80 M in Et_2O, 0.25 mL, 0.2 mmol, 1.0 eq.) in DMA (1.0 mL) were submitted to General Procedure 1A. Purification by column chromatography (SiO_2, pentane / EtOAc, 9:1) gave **3p** (21 mg, 43%) as a yellow oil.

R_f = 0.42 (pentane / EtOAc, 7:3).

^{1}H NMR (400 MHz, $CDCl_3$) δ 8.13 (1H, app. s, H9), 7.83 (1H, d, J = 4.5 Hz, H10), 7.05 (1H, dd, J = 8.6, 4.5 Hz, H11), 6.93 (1H, ddd, J = 8.6, 3.2, 1.3 Hz, H12), 3.64 (1H, dd, J = 11.5, 6.2 Hz, H3), 3.53 – 3.43 (1H, m, H8), 3.17 (1H, ddd, J = 15.6, 11.5, 1.8 Hz, H8), 2.47 (1H, s, H1), 2.06 – 1.94 (1H, m, H4), 1.81 – 1.37 (11H, m, H2, H4, H7), 1.35 – 1.21 (1H, m, H6), 1.20 – 1.07 (1H, m, H5).

^{13}C NMR (101 MHz, $CDCl_3$) δ 145.1, 136.0, 133.4, 123.5, 116.5, 56.7, 49.4, 47.3, 43.4, 31.7, 30.0, 27.9, 26.8, 25.3.

HRMS (ESI$^+$) calc. for $C_{16}H_{22}N_2$ $[M+H]^+$ 243.1856, found 243.1856.

IR (film, ν_{max}/cm^{-1}) 2962, 2925, 2868, 1578, 1490, 1168.

3-(Bicyclo[1.1.1]pentan-1-yl)-4-phenylmorpholine, 3q and 4q

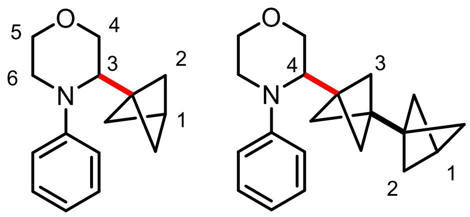

1-phenylmorpholine (326 mg, 2.0 mmol, 10.0 eq.), 4CzIPN (3.9 mg, 5.0 µmol, 2.5 mol%), deionised H_2O (36 µl, 10 equiv.) and [1.1.1]propellane (0.80 M in Et_2O, 0.25 mL, 0.2 mmol, 1.0 eq.) in DMA (1.0 mL) were submitted to General Procedure 1A. Purification by column chromatography (SiO_2, pentane / EtOAc, 95:5) gave **3q** and **4q** (17 mg of a 10:1 ratio of **3q**:**4q**, 40%) as a colourless oil.

$\mathbf{R_f}$ = 0.33 (pentane / EtOAc, 95:5).

^{1}H NMR (400 MHz, $CDCl_3$) δ 7.25 – 7.19 (2H, m, ArH), 6.93 – 6.85 (2H, m, ArH), 6.83 – 6.77 (1H, m, ArH), 4.01 – 3.93 (2H, m, H4, H5), 3.75 (1H, dd, *J* = 11.3, 3.5 Hz, H4), 3.71 – 3.61 (2H, m, H3, H5), 3.38 (1H, ddd, *J* = 12.1, 10.9, 3.6 Hz, H6), 3.12 (1H, dt, *J* = 12.1, 2.6 Hz, H6), 2.34 (1H, s, H1), 1.73 – 1.65 (6H, m, H2).

^{13}C NMR (101 MHz, $CDCl_3$) δ 150.9, 129.1, 119.0, 115.5, 68.7, 67.0, 54.6, 52.4, 46.9, 44.6, 28.5.

HRMS (ESI^+) calc. for $C_{15}H_{20}ON$ $[M+H]^+$ 230.1539, found 230.1541.

IR (film, ν_{max}/cm^{-1}) 2962, 2904, 2868, 1598, 1500, 1121.

Characteristic peaks for staffane product:

^{1}H NMR (400 MHz, $CDCl_3$) δ 2.32 (1H, s, H1), 1.52 (6H, s, H2), 1.47 (6H, m, H3).

^{13}C NMR (101 MHz, $CDCl_3$) δ 51.2, 49.2.

HRMS (ESI^+) calc. for $C_{20}H_{26}ON$ $[M+H]^+$ 296.2009, found 296.2010.

1-(Bicyclo[1.1.1]pentan-1-yl)-2-(pyridin-3-yl)octahydrocyclopenta[c]pyrrole, 3r

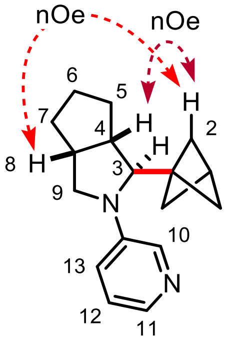

2-(Pyridin-3-yl)octahydrocyclopenta[c]pyrrole (376 mg, 2.0 mmol, 10.0 eq.), 4CzIPN (3.9 mg, 5.0 µmol, 2.5 mol%), deionised H_2O (36 µl, 10 equiv.) and [1.1.1]propellane (0.80 M in Et_2O, 0.25 mL, 0.2 mmol, 1.0 eq.) in DMA (0.4 mL) were submitted to General Procedure 1A. Purification by column chromatography (SiO_2, pentane / EtOAc, 2:1) gave **3r** (36 mg, 71%) as a yellow oil.

$\mathbf{R_f}$ = 0.45 (pentane / EtOAc, 1:1).

^{1}H NMR (400 MHz, $CDCl_3$) δ ^{1}H NMR (400 MHz, $CDCl_3$) δ 8.00 (1H, d, J = 2.9 Hz, H10), 7.86 (1H, d, J = 4.6 Hz, H11), 7.05 (1H, dd, J = 8.5, 4.6 Hz, H12), 6.84 (1H, ddd, J = 8.5, 3.0, 1.3 Hz, H13), 3.74 (1H, s, H3), 3.53 (1H, t, J = 9.4 Hz, H9), 2.94 (1H, dd, J = 9.4, 5.5 Hz, H9), 2.85 – 2.73 (1H, m, H8), 2.47 (1H, q, J = 8.5 Hz, H4), 2.43 (1H, s, H1), 1.96 – 1.80 (2H, m, H5, H7), 1.72 – 1.63 (7H, m, H2, H6) 1.58 – 1.48 (2H, m,, H6, H7), 1.34 – 1.22 (1H, m, H5).

^{13}C NMR (101 MHz, $CDCl_3$) δ 144.1, 136.4, 134.7, 123.5, 118.6, 64.9, 54.5, 50.1, 47.8, 46.5, 41.6, 33.1, 32.8, 27.8, 25.5.

HRMS (ESI$^+$) calc. for $C_{17}H_{23}N_2$ $[M+H]^+$ 255.1856, found 255.1856.

IR (film, ν_{max}/cm^{-1}) 2944, 2962, 1582, 1481, 1364.

***tert*-Butyl 2-(bicyclo[1.1.1]pentan-1-yl)-1-(pyridin-3-yl)pyrrolidin-3-yl)carbamate (3s) AND *tert*-butyl 5-(bicyclo[1.1.1]pentan-1-yl)-1-(pyridin-3-yl)pyrrolidin-3-yl)carbamate (3t)**

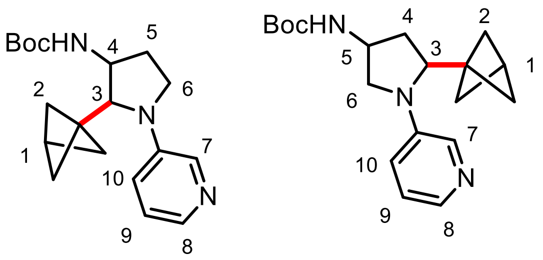

2s **2t**

tert-butyl (1-(pyridin-3-yl)pyrrolidin-3-yl)carbamate (527 mg, 2.0 mmol, 10.0 eq.), 4CzIPN (3.9 mg, 5.0 µmol, 2.5 mol%), deionised H_2O (36 µl, 10 equiv.) and [1.1.1]propellane (0.80 M in Et_2O, 0.25 mL, 0.2 mmol, 1.0 eq.) in DMA (1.0 mL) were submitted to General Procedure 1A for 48 h. Purification by column chromatography (SiO_2, EtOAc) gave an inseparable mixture of **3s** and **3t** (41 mg of a 1.5:1 mixture, 61%) as a yellow oil.

R$_f$ = 0.41 (EtOAc).

HRMS (ESI$^+$) calc. for $C_{19}H_{28}O_2N_3$ $[M+H]^+$ 330.2176, found 330.2175.

IR (film, ν_{max}/cm^{-1}) 3221, 2970, 1717, 1537, 1156.

Data for 3s

^{1}H NMR (400 MHz, $CDCl_3$) δ 8.04 – 7.98 (1H, m, H7), 7.97 – 7.88 (1H, m, H8), 7.15 – 7.06 (1H, m, ArH), 6.91 – 6.81 (1H, m, ArH), 4.64 (1H, s, NH), 4.14 – 4.07 (1H, m, H4), 3.67 (1H, s, H3), 3.48 (1H, td, *J* = 9.6, 1.6 Hz, H6), 3.28 (1H, td, *J* = 9.6, 7.2 Hz, H6), 2.47 (1H, s, H1), 2.37 – 2.15 (1H, m, H5), 1.98 (1H, dd, *J* = 13.8, 7.2 Hz, H5), 1.81 – 1.74 (6H, m, H2), 1.42 (9H, s, *t*Bu).

^{13}C NMR (101 MHz, $CDCl_3$) δ 155.2, 143.9, 136.9, 133.9, 123.7, 118.7, 79.9, 65.8, 53.5, 50.2, 46.0, 45.9, 30.3, 28.5, 27.7.

Data for 3t

^{1}H NMR (400 MHz, $CDCl_3$) δ 8.04 – 7.98 (1H, m, H7), 7.97 – 7.88 (1H, m, H8), 7.15 – 7.06 (1H, m, ArH), 6.91 – 6.81 (1H, m, ArH), 4.64 (1H, s, NH), 4.48 – 4.41 (1H, m, H4), 3.86 (1H, d, *J* = 7.5 Hz, H3), 3.80 (1H, t, *J* = 8.0 Hz, H6), 2.89 (1H, dd, *J* = 9.3, 7.9 Hz, H6), 2.47 (1H, s, H1), 2.34 – 2.24 (1H, m, H4), 1.77 – 1.67 (7H, m, H2, H4), 1.45 (9H, s, *t*Bu).

^{13}C NMR (101 MHz, $CDCl_3$) δ 155.6, 143.9, 137.0, 134.0, 123.7, 118.6, 79.9, 58.0, 54.0, 49.7, 49.1, 47.3, 35.2, 28.5, 27.5.

b) Synthesis of Bicyclo[3.1.1.]heptanes

2-(Bicyclo[3.1.1]heptan-1-yl)-1-phenylpyrrolidine, 8a and 9a

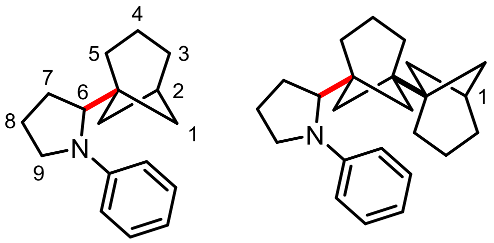

1-Phenylpyrollidine (294 mg, 2.0 mmol, 10.0 eq.), 4CzIPN (3.9 mg, 5.0 µmol, 2.5 mol%), deionised H_2O (36 µl, 10 equiv.) and [3.1.1]propellane (0.35 M in *n*-Bu_2O, 0.58 mL, 0.2 mmol, 1.0 eq.) in DMA (0.5 mL) were submitted to General Procedure 1B for 16 h. Purification by column chromatography (SiO_2, pentane) gave **8a** and **9a** (40 mg of a 5:1 ratio of **8a**:**9a**, 82%) as a colourless oil.

$\mathbf{R_f}$ = 0.19 (pentane).

^{1}H NMR (400 MHz, $CDCl_3$) δ 7.21 – 7.17 (2H, m, ArH), 6.64 (1H, t, *J* = 7.3 Hz, ArH), 6.57 (2H, t, *J* = 7.7 Hz, ArH), 3.58 (1H, ddd, *J* = 9.2, 8.0, 2.5 Hz, H9), 3.42 (1H, dd, *J* = 7.5, 2.7 Hz, H6), 3.19 (1H, td, *J* = 9.3, 7.4 Hz, H9), 2.13 (1H, tt, *J* = 6.4, 3.1 Hz, H2), 1.93 – 1.65 (12H, m, H1, H3/H5, H4, H7, H8), 1.34 – 1.30 (2H, m, H3/H5).

^{13}C NMR (126 MHz, $CDCl_3$) δ 149.8, 128.8, 115.5, 112.7, 64.8, 50.6, 48.3, 35.7, 34.0, 32.7, 30.2, 28.8, 27.6, 24.4, 17.1.

HRMS (ESI$^+$) calc. for $C_{17}H_{24}N$ [M+H]$^+$ 242.1903, found 242.1898.

IR (film, ν_{max}/cm^{-1}) 2937, 2856, 1599, 1504, 1451, 1362, 1338, 1125, 933, 748.

Characteristic peaks for staffane product:

^{1}H NMR (400 MHz, $CDCl_3$) δ 2.07 (1H, tt, *J* = 6.3, 3.4 Hz, H1).

HRMS (ESI$^+$) calc. for $C_{24}H_{34}N$ [M+H]$^+$ 336.2686, found 336.2689.

2-(Bicyclo[3.1.1]heptan-1-yl)-1-(4-(methylsulfonyl)phenyl)pyrrolidine, 8b

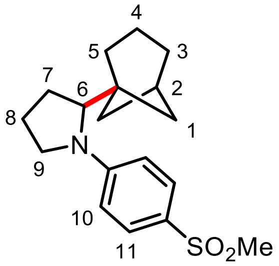

1-(4-(Methylsulfonyl)phenyl)pyrrolidine (450 mg, 2.0 mmol, 10.0 eq.), 4CzIPN (3.9 mg, 5.0 µmol, 2.5 mol%), deionised H_2O (36 µl, 10 equiv.) and [3.1.1]propellane (0.35 M in *n*-Bu_2O, 0.58 mL, 0.2 mmol, 1.0 eq.) in DMA (0.5 mL) were submitted to General Procedure 1B for 16 h. Purification by column chromatography (SiO_2, pentane / EtOAc, 7:3) gave **8b** (33 mg, 52%) as a white solid.

R_f = 0.23 (pentane / EtOAc, 8:2).

m.p. = 142–144 °C ($CDCl_3$).

^{1}H NMR (400 MHz, $CDCl_3$) 7.69 (2H, d, *J* = 9.0 Hz, H11), 6.58 (2H, d, *J* = 9.0 Hz, H10), 3.62 – 3.54 (2H, m, H6, H9), 3.29 (1H, q, *J* = 9.0 Hz, H9), 3.00 (3H, s, Me), 2.16 (1H, tt, *J* = 6.4, 3.1 Hz, H2), 2.05 – 1.93 (2H, m, H7, H8), 1.89 – 1.77 (5H, m, H1, H3/H5, H7, H8), 1.74 – 1.69 (5H, m, H1, H4), 1.39 (1H, t, *J* = 8.2 Hz, H3/H5), 1.31 (1H, t, *J* = 8.3 Hz, H3/H5).

^{13}C NMR (101 MHz, $CDCl_3$) δ 152.7, 128.8, 125.5, 112.2, 65.0, 50.2, 48.0, 45.3, 35.8, 34.7, 32.5, 30.3, 28.5, 27.6, 24.0, 17.0.

HRMS (ESI^+) calc. for $C_{18}H_{26}NO_2S$ $[M+H]^+$ 320.1679, found 320.1690.

IR (film, ν_{max}/cm^{-1}) 2937, 2928, 2856, 1595, 1508, 1375, 1305, 1144, 957, 770.

Methyl 4-(2-(bicyclo[3.1.1]heptan-1-yl)pyrrolidin-1-yl)benzoate, 8c

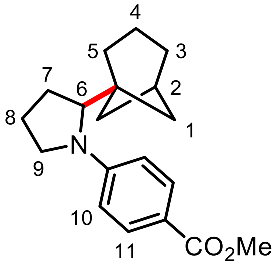

Methyl 4-(pyrrolidin-1-yl)benzoate (410 mg, 2.0 mmol, 10.0 eq.), 4CzIPN (3.9 mg, 5.0 µmol, 2.5 mol%), deionised H_2O (36 µl, 10 equiv.) and [13.1.1]propellane (0.35 M in *n*-Bu_2O, 0.58 mL, 0.2 mmol, 1.0 eq.) in DMA (0.5 mL) were submitted to General Procedure 1B for 16 h. Purification by column chromatography (SiO_2, pentane / EtOAc, 9:1) gave **8c** (44 mg, 73%) as a colourless oil.

$\mathbf{R_f}$ = 0.48 (pentane / EtOAc, 9:1).

^{1}H NMR (600 MHz, $CDCl_3$) 7.86 (2H, d, J = 9.0 Hz, H11), 6.54 (2H, d, J = 8.8 Hz, H10), 3.85 (3H, s, OMe), 3.61 (1H, ddd, J = 9.6, 8.4, 2.8 Hz, H9), 3.55 (1H, dd, J = 7.7, 2.7 Hz, H6), 3.32 – 3.24 (1H, m, H9), 2.14 (1H, tt, J = 6.4, 3.2 Hz, H2), 2.02 – 1.90 (2H, m, H7, H8), 1.88 – 1.77 (5H, m, H1, H3/H5, H7, H8), 1.75 – 1.67 (5H, m, H1, H4), 1.37 (1H, t, J = 8.2 Hz, H3/H5), 1.30 (1H, t, J = 8.3 Hz, H3/H5).

^{13}C NMR (151 MHz, $CDCl_3$) δ 167.7, 152.6, 131.1, 116.6, 111.9, 64.8, 51.6, 50.2, 48.2, 35.8, 34.6, 32.6, 30.3, 28.6, 27.5, 24.0, 17.0.

HRMS (ESI$^+$) calc. for $C_{19}H_{26}NO_2$ $[M+H]^+$ 300.1958, found 300.1961.

IR (film, ν_{max}/cm^{-1}) 2929, 2856, 1712, 1607, 1540, 1435, 1370, 1201, 1183, 1109, 770.

2-(Bicyclo[3.1.1]heptan-1-yl)-1-(4-fluorophenyl)pyrrolidine, 8d and 9d

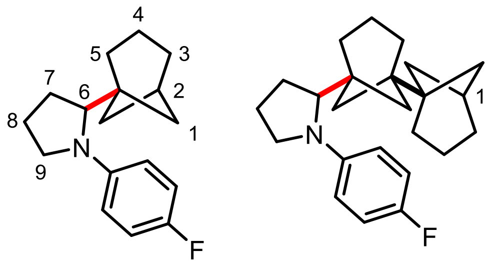

1-(4-Fluorophenyl)pyrollidine (300 mg, 2.0 mmol, 10.0 eq.), 4CzIPN (3.9 mg, 5.0 µmol, 2.5 mol%), deionised H_2O (36 µl, 10 equiv.) and [3.1.1]propellane (0.35 M in *n*-Bu_2O, 0.58 mL, 0.2 mmol, 1.0 eq.) in DMA (0.5 mL) were submitted to General Procedure 1B for 16 h. Purification by column chromatography (Al_2O_3, pentane) gave **8d** and **9d** (11 mg of a 5:1 ratio of **8d**:**9d**, 21%) as a colourless oil.

$\mathbf{R_f}$ = 0.45 (Al_2O_3, pentane).

^{1}H NMR (400 MHz, $CDCl_3$) δ 6.91 – 6.88 (2H, m, ArH), 6.48 – 6.45 (2H, m, ArH), 3.57 (1H, td, *J* = 8.8, 2.4 Hz, H9), 3.34 – 3.32 (1H, m, H6), 3.15 – 3.10 (1H, m, H9), 2.14 (1H, tt, *J* = 6.3, 3.2 Hz, H2), 1.86 – 1.68 (12H, m, H1, H3/H5, H4, H7, H8), 1.33 (1H, t, *J* = 8.1 Hz, H3/H5), 1.28 (1H, t, *J* = 8.2 Hz, H3/H5).

^{13}C NMR (126 MHz, $CDCl_3$) δ 154.9 (d, $^{1}J_{CF}$ = 233.3 Hz), 146.5, 115.1 (d, $^{2}J_{CF}$ = 22.0 Hz), 113.1 (d, $^{3}J_{CF}$ = 7.0 Hz), 65.3, 51.2, 48.3, 35.7, 33.9, 32.7, 30.2, 28.8, 27.7, 24.6, 17.1.

^{19}F NMR (377 MHz, $CDCl_3$) δ –131.1 (1H, tt, *J* = 8.6, 4.4 Hz, H1).

HRMS (ESI$^+$) calc. for $C_{17}H_{23}FN$ [M+H]$^+$ 260.1809, found 260.1812.

IR (film, ν_{max}/cm^{-1}) 2939, 2856, 1516, 1363, 1341, 1228, 1159, 812.

Characteristic peaks for staffane product:

^{1}H NMR (400 MHz, $CDCl_3$) δ 2.08 (1H, tt, *J* = 6.3, 3.2 Hz, H1).

^{19}F NMR (377 MHz, $CDCl_3$) δ –131.2 (1H, tt, *J* = 8.6, 4.4 Hz, H1).

HRMS (ESI$^+$) calc. for $C_{24}H_{33}FN$ [M+H]$^+$ 354.2592, found 354.2589.

3-(2-(Bicyclo[3.1.1]heptan-1-yl)pyrrolidin-1-yl)benzonitrile, 8e

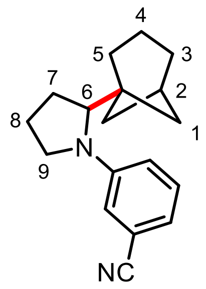

3-(Pyrrolidin-1-yl)benzonitrile (344 mg, 2.0 mmol, 10.0 eq.), 4CzIPN (3.9 mg, 5.0 µmol, 2.5 mol%), deionised H_2O (36 µl, 10 equiv.) and [3.1.1]propellane (0.35 M in *n*-Bu_2O, 0.58 mL, 0.2 mmol, 1.0 eq.) in DMA (0.5 mL) were submitted to General Procedure 1B. Purification by column chromatography (SiO_2, pentane / EtOAc, 95:5) gave **8e** (11 mg, 20%) as a colourless oil.

R_f = 0.46 (pentane / EtOAc, 95:5).

^{1}H NMR (600 MHz, $CDCl_3$) δ 7.23 – 7.20 (1H, m, ArH), 6.89 (1H, dt, *J* = 7.5, 1.2 Hz, ArH), 6.76 – 6.72 (2H, m, ArH), 3.56 (1H, ddd, *J* = 9.2, 8.1, 2.7 Hz, H9), 3.42 (1H, dd, *J* = 7.7, 2.6 Hz, H6), 3.18 (1H, td, *J* = 9.3, 7.5 Hz, H9), 2.15 (1H, tt, *J* = 6.4, 3.2 Hz, H2), 2.02 – 1.96 (1H, m, H7/H8), 1.95 – 1.90 (1H, m, H7/H8), 1.86 – 1.80 (5H, m, H1, H3/H5, H7, H8), 1.74 – 1.68 (5H, m, H1, H4), 1.36 (1H, t, *J* = 8.2 Hz, H3/H5), 1.31 (1H, t, *J* = 8.3 Hz, H3/H5).

^{13}C NMR (126 MHz, $CDCl_3$) δ 149.4, 129.4, 120.2, 118.8, 116.9, 115.5, 112.5, 65.0, 50.4, 48.1, 35.6, 34.3, 32.6, 30.3, 28.6, 27.6, 24.2, 17.0.

HRMS (ESI^+) calc. for $C_{18}H_{23}N_2$ $[M+H]^+$ 267.1856, found 267.1861.

IR (film, ν_{max}/cm^{-1}) 2971, 2873, 2228, 1598, 1495, 1366, 1208, 1015, 779.

2-(2-(Bicyclo[3.1.1]heptan-1-yl)pyrrolidin-1-yl)pyridine, 8f and 9f

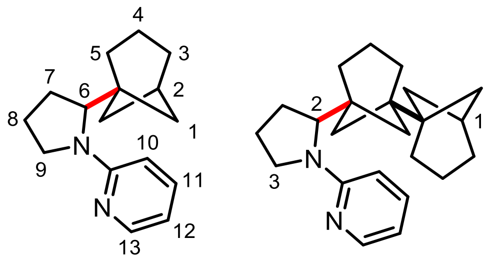

2-(Pyrrolidin-1-yl)pyridine (296 mg, 2.0 mmol, 10.0 eq.), 4CzIPN (3.9 mg, 5.0 µmol, 2.5 mol%), deionised H_2O (36 µl, 10 equiv.) and [3.1.1]propellane (0.35 M in *n*-Bu_2O, 0.58 mL, 0.2 mmol, 1.0 eq.) in DMA (0.5 mL) were submitted to General Procedure 1B. Purification by column chromatography (SiO_2, pentane / EtOAc, 9:1) gave **8f** and **9f** (21 mg of a 10:1 ratio of **8f**:**9f**, 43%) as a colourless oil.

$\mathbf{R_f}$ = 0.4 (pentane / EtOAc, 9:1).

^{1}H NMR (600 MHz, $CDCl_3$) δ 8.14 (1H, dd, J = 4.9, 1.2 Hz, H13), 7.37 (1H, ddd, J = 8.8, 7.1, 2.0 Hz, H11), 6.50 (1H, dd, J = 6.6, 5.4 Hz, H12), 6.33 (1H, d, J = 8.5 Hz, H10), 3.72 – 3.70 (1H, m, H9), 3.61 – 3.59 (2H, m, H6, H9), 2.14 (1H, tt, J = 6.4, 3.2 Hz, H2), 1.96 – 1.90 (1H, m, H7/H8), 1.88 – 1.77 (6H, m, H1, H3/H5, H7, H8), 1.75 – 1.66 (5H, m, H1, H4), 1.35 (1H, t, J = 8.1 Hz, H3/H5), 1.31 (1H, t, J = 8.2 Hz, H3/H5).

^{13}C NMR (151 MHz, $CDCl_3$) δ 159.0, 148.0, 136.5, 111.4, 107.6, 63.8, 49.2, 48.1, 35.4, 34.7, 32.4, 30.2, 28.7, 27.5, 24.2, 17.1.

HRMS (ESI$^+$) calc. for $C_{16}H_{23}N_2$ $[M+H]^+$ 243.1856, found 243.1851.

IR (film, ν_{max}/cm^{-1}) 2939, 2910, 2856, 1597, 1479, 1439, 1375, 1323, 991, 770.

Characteristic peaks for staffane product:

^{1}H NMR (600 MHz, $CDCl_3$) δ 3.76 (1H, dd, J = 7.4, 3.3 Hz, H3), 3.57 – 3.54 (2H, m, H2/H3), 2.08 (1H, tt, J = 6.2, 3.0 Hz, H1).

HRMS (ESI$^+$) calc. for $C_{23}H_{33}N_2$ $[M+H]^+$ 337.2638, found 337.2638.

3-(Bicyclo[3.1.1]heptan-1-yl)-4-phenylmorpholine, 8g

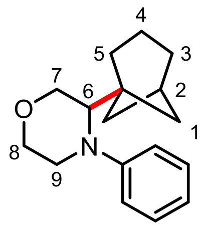

4-Phenylmorpholine (326 mg, 2.0 mmol, 10.0 eq.), 4CzIPN (3.9 mg, 5.0 µmol, 2.5 mol%), deionised H_2O (36 µl, 10 equiv.) and [3.1.1]propellane (0.35 M in *n*-Bu_2O, 0.58 mL, 0.2 mmol, 1.0 eq.) in DMA (0.5 mL) were submitted to General Procedure 1B. Purification by column chromatography (SiO_2, pentane / EtOAc, 85:15) gave **8g** (29 mg, 56%) as a colourless oil.

R_f = 0.69 (pentane / EtOAc, 8:2).

^{1}H NMR (400 MHz, $CDCl_3$) δ 7.26 – 7.22 (2H, m, ArH), 6.97 (2H, d, *J* = 8.1 Hz, ArH), 6.89 (1H, t, *J* = 7.2 Hz, ArH), 3.86– 3.78 (2H, m, H7, H8), 3.75– 3.69 (2H, m, H7, H8), 3.26 – 3.15 (3H, m, H6, H9), 2.12 (1H, tt, *J* = 6.4, 3.1 Hz, H2), 1.85 – 1.58 (8H, m, H1, H4, H3/H5), 1.29 (1H, t, *J* = 8.4 Hz, H3/H5), 0.97 (1H, t, *J* = 8.7 Hz, H3/H5).

^{13}C NMR (101 MHz, $CDCl_3$) δ 151.5, 129.1, 120.7, 119.3, 67.3, 67.0, 62.9, 49.0, 46.3, 38.8, 37.0, 32.1, 31.0, 28.6, 16.9.

HRMS (ESI$^+$) calc. for $C_{17}H_{24}NO$ $[M+H]^+$ 258.1852, found 258.1853.

IR (film, ν_{max}/cm^{-1}) 2940, 2856, 1598, 1503, 1451, 1230, 1124, 934, 750.

3-((2*S*,5*S*)-5-(Bicyclo[1.1.1]pentan-1-yl)-1-methylpyrrolidin-2-yl)pyridine, 7 and 7-staff

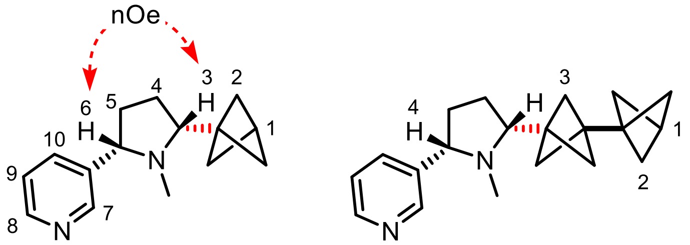

(*S*)-3-(1-Methylpyrrolidin-2-yl)pyridine (320 mg, 2.0 mmol, 10.0 eq.), 4CzIPN (3.9 mg, 5.0 µmol, 2.5 mol%), deionised H_2O (36 µl, 10 equiv.) and [1.1.1]propellane (0.81 M in Et_2O, 0.25 mL, 0.5 mmol, 1.0 eq.) in DMA (0.5 mL) were submitted to General Procedure 1A. Purification by column chromatography (SiO_2, pentane / EtOAc, 95:05) gave **7** and **7-staff** (17 mg of a 4:1 ratio of **7**:**7-staff**, 37%) as a colourless oil.

R_f = 0.40 (pentane / EtOAc, 8:2).

^{1}H NMR (400 MHz, $CDCl_3$) δ 8.56 – 8.55 (1H, m, H7), 8.47 (1H, dd, *J* = 4.8, 1.8 Hz, H8), 7.72 (1H, dt, *J* = 7.8, 2.0 Hz, H10), 7.23 (1H, dd, *J* = 8.0, 4.8 Hz, H9), 3.33 (1H, dd, *J* = 9.3, 6.7 Hz, H6), 2.53 (s, 1H, H1), 2.48 (1H, dd, *J* = 8.8, 6.0 Hz, H3), 2.16 (s, 1H, CH_3), 2.02 (1H, dddd, *J* = 12.2, 8.5, 6.6, 3.7 Hz, H4/H5), 1.86 – 1.76 (2H, m, H4/H5), 1.77 – 1.71 (6H, m, H2), 1.66 (1H, dtd, *J* = 12.5, 5.9, 3.1 Hz, H4/H5).

^{13}C NMR (151 MHz, $CDCl_3$) δ 149.6, 148.6, 139.9, 134.9, 123.6, 70.2, 66.2, 49.7, 48.8, 40.6, 34.8, 28.2, 26.9.

IR (film, ν_{max}/cm^{-1}) 2966, 2907, 2871, 1577, 1428, 1213, 1026, 717.

Characteristic peaks for staffane product **7-staff**:

^{1}H NMR (400 MHz, $CDCl_3$) δ 3.41 (1H, td, *J* = 8.5, 3.2 Hz, H4), 2.37 (1H, s, H1), 1.59 (6H, s, H2), 1.50 – 1.42 (6H, m, H3).

^{13}C NMR (151 MHz, $CDCl_3$) δ 49.3, 47.7, 23.2.

HRMS (ESI$^+$) calc. for $C_{20}H_{27}N_2$ [M+H]$^+$ 295.2169, found 295.2177.

We propose that the stereochemical outcome of this reaction can be explained by torsional strain arguments. We conducted DFT calculations (at the CPCM(MeCN)-PBE0-D3(BJ)/def2-TZVP//CPCM(MeCN)-PBE0-D3(BJ)/def2-SVP level of theory) on the intermediate alpha-amino radical **6-rad2** (see Scheme below and page S51), and its fate on reaction with [1.1.1]propellane from either face of the pyrrolidine ring to give either **7-BCP-*syn*** (major) or **7-BCP-*anti*** (minor). The energy of the latter is found to be 2.8 kcal mol^{-1} higher, which we propose will be reflected in the transition states for ring opening, explaining the observed selectivity.

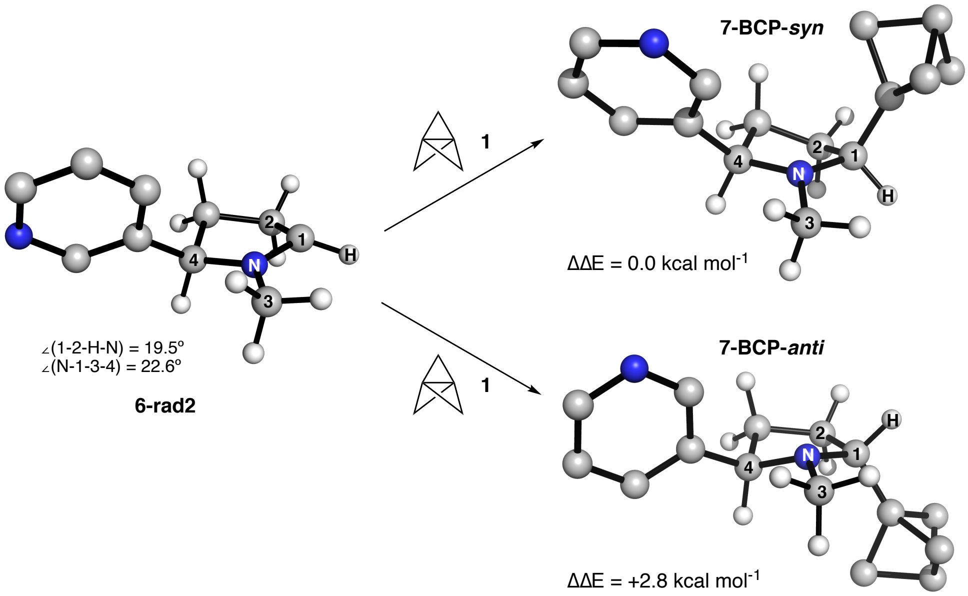

Radical **6-rad2** displays quite pyramidal character at both the N and C1 atoms. On reaction with [1.1.1]propellane, the pathway to **7-BCP-*syn*** results in a staggered arrangement around the C1–C2 bond, while the pathway to **7-BCP-*anti*** results in an eclipsed arrangement (and requires inversion at the carbon atom). We propose that the selectivity in the reaction may therefore arise from developing (eclipsing) torsional strain in the disfavoured *anti*-isomer. This argument is reminiscent of the reactions of cyclic oxocarbenium ions as explored by the Woerpel group, e.g. see Larsen *et al. J. Am. Chem. Soc.* **1999**, *121*, 12208), where similar torsional strain-based models are proposed.

c) Substrate Synthesis

1-(4-(Methylsulfonyl)phenyl)pyrrolidine

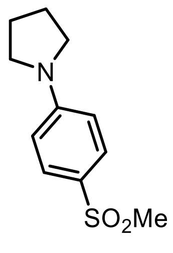

4-Bromophenyl methyl sulfone (4.7 g, 20.0 mmol, 1.0 eq.) was submitted to General Procedure 2 for 72 h. Purification by column chromatography (SiO_2, 2:1 → 1:1) gave 1-(4-(methylsulfonyl)phenyl)pyrrolidine (2.4 g, 53%) as a tan solid.

^{1}H NMR (400 MHz, $CDCl_3$) δ 7.72 (d, *J* = 8.9 Hz, 1H), 6.56 (d, *J* = 8.9 Hz, 1H), 3.48 – 3.25 (m, 4H), 2.99 (s, 3H), 2.10 – 2.00 (m, 4H).

Analytical data matches that previously reported.[3]

3-(Pyrrolidin-1-yl)benzonitrile

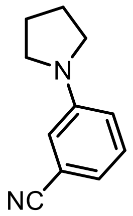

4-Bromophenyl methyl sulfone (4.6 g, 20.0 mmol, 1.0 eq.) was submitted to General Procedure 2 for 72 h. Purification by column chromatography (SiO_2, 9:1) gave 3-(pyrrolidin-1-yl)benzonitrile (2.6 g, 75%) as a tan solid.

^{1}H NMR (400 MHz, $CDCl_3$) δ 7.29 – 7.20 (m, 1H), 6.89 (dt, *J* = 7.5, 1.3 Hz, 1H), 6.76 – 6.68 (m, 2H), 3.35 – 3.23 (m, 4H), 2.15 – 1.94 (m, 4H).
Analytical data matches that previously reported.[4]

6-(pyrrolidin-1-yl)quinoline

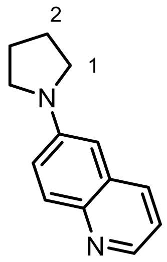

6-Bromoquinoline (1.4 mL, 10.0 mmol, 1.0 eq.) was submitted to General Procedure 2 (performed at half scale) for 48 h. Purification by column chromatography (SiO_2, 1:1) gave 6-(pyrrolidin-1-yl)quinoline (1.62 g, 82%) as a yellow solid.

^{1}H NMR (400 MHz, $CDCl_3$) δ 8.57 (dd, *J* = 4.5, 1.6 Hz, 1H), 7.95 – 7.85 (m, 2H), 7.25 – 7.12 (m, 2H), 6.59 (t, *J* = 2.7 Hz, 1H), 3.69 – 2.86 (m, 4H), 2.12 – 1.95 (m, 4H).

Analytical data matches that previously reported.[5]

1-Phenylazepane

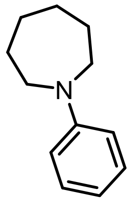

Bromobenzene (2.2 mL, 20.0 mmol, 1.0 eq.) and azepane (6.7 mL, 60.0 mmol, 3.0 equiv.) was submitted to General Procedure 2 for 72 h. Purification by column chromatography (SiO_2, pentane)

gave 1-phenylazepane (2.7 g, 77%) as a yellow oil.

^{1}H NMR (400 MHz, $CDCl_3$) δ 7.25 – 7.17 (m, 2H), 6.69 (d, J = 8.2 Hz, 2H), 6.63 (t, J = 7.2 Hz, 1H), 3.49 – 3.42 (m, 4H), 1.84 – 1.75 (m, 4H), 1.58 – 1.52 (m, 4H).

Analytical data matches that previously reported.[6]

1-(Pyridin-3-yl)azepane

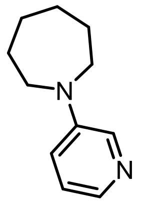

3-Bromopyridine (1.9 mL, 20.0 mmol, 1.0 eq.) and azepane (6.7 mL, 60.0 mmol, 3.0 equiv.) was submitted to General Procedure 2 for 72 h. Purification by column chromatography (SiO_2, 1:1) gave 1-(pyridin-3-yl)azepane (2.3 g, 64%) as a yellow oil.

^{1}H NMR (400 MHz, $CDCl_3$) δ 8.11 (d, J = 3.1 Hz, 1H), 7.93 – 7.85 (m, 1H), 7.11 – 7.03 (m, 1H), 6.98 – 6.86 (m, 1H), 3.49 – 3.40 (m, 4H), 1.85 – 1.74 (m, 4H), 1.58 – 1.49 (m, 4H).

Analytical data matches that previously reported.[7]

2-(Pyridin-3-yl)octahydrocyclopenta[*c*]pyrrole

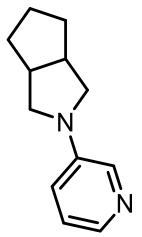

To a flame dried flask containing K_2CO_3 (4.15 g, 30.0 mmol, 3.0 equiv.), proline (231 mg, 2.0 mmol, 20 mol %), and CuI (191 mg, 1.0 mmol, 10 mol%) in anhydrous DMSO (20 mL) was added Octahydrocyclopenta[c]pyrrole hydrochloride (3.0 g, 20.0 mmol, 2.0 equiv.) and 3-bromopyridine (0.95 mL, 10 mmol, 1.0 equiv.). The resulting slurry was heated at 100 °C for 72 h then diluted with EtOAc and washed with NH4OH (10 mL, 30% aq.). The layers were separated, and the aqueous layer was extracted with EtOAc (2 × 5 mL). The combined organic layers were washed with brine, dried over $MgSO_4$ and concentrated *in vacuo*. Purification by column chromatography (SiO_2, 1:1) gave (1.0 g, 53%) as a yellow oil.

R_f = 0.41 (pentane / EtOAc, 1:1);

^{1}H NMR (400 MHz, $CDCl_3$) δ 8.01 (d, *J* = 2.9 Hz, 1H), 7.94 (d, *J* = 4.7 Hz, 1H), 7.09 (dd, *J* = 8.4, 4.7 Hz, 1H), 6.87 – 6.79 (m, 1H), 3.51 – 3.30 (m, 2H), 3.04 (dd, *J* = 9.6, 3.3 Hz, 2H), 2.86 – 2.72 (m, 2H), 1.96 – 1.80 (m, 2H), 1.81 – 1.45 (m, 4H).

^{13}C NMR (101 MHz, $CDCl_3$) δ 144.6, 137.8, 135.6, 123.6, 119.1, 54.9, 42.9, 33.1, 25.9.

HRMS (ESI^+) calc. for $C_{12}H_{17}N_2$ $[M+H]^+$ 189.1388, found 189.1386;

IR (film, ν_{max}/cm^{-1}) 2946, 1697, 1582, 1479, 1364.

d$_4$-*N*-phenylpyrollidine

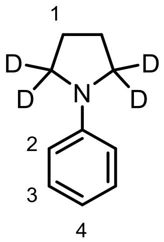

To a solution of $NaBD_4$ (1.7 g, 40 mmol, 5.0 eq.) in anhydrous THF (50 mL) at 0 °C was slowly added a solution of I_2 (5.3 g, 20.8 mmol, 2.6 eq.) in anhydrous THF (25 mL) of 1 h (Caution: H_2 release). A solution of *N*-phenylsuccinimide (1.4 g, 8 mmol, 1.0 eq.) in anhydrous THF (15 mL) was added and the resulting solution was heated at reflux for 16 h. The reaction was cooled to 0 °C and quenched by the slow addition of 3 M NaOH (50 mL) until effervescence ceased. EtOAc (50 mL) was added and the layers were separated. The aqueous layer was extracted with EtOAc (2 × 5 mL), the combined organic layers were washed with brine, dried over $MgSO_4$ and concentrated *in vacuo*. Purification by column chromatography (SiO_2, pentane) gave d$_4$-*N*-phenylpyrollidine (1.1 g, 7.3 mmol, 91%, 98% D incorporation by ^{1}H NMR).

^{1}H NMR (400 MHz, $CDCl_3$) δ δ 7.26 – 7.20 (2H, m, H3), 6.66 (1H, t, *J* = 7.3 Hz, H4), 6.58 (1H, d, *J* = 8.1 Hz, H3), 1.99 (4H, s, H1).

^{13}C NMR (101 MHz, $CDCl_3$) δ 129.3, 115.5, 111.8, 46.8, 25.4.

HRMS (ESI^+) calc. for $C_{10}H_{10}D_4N$ $[M+H]^+$ 152.1372, found 152.137.

IR (film, ν_{max}/cm^{-1}) 2964, 2875, 2081, 1599, 1503.

11. Computational details

Computational methods

Conformationally-sampled intermediates and transition state structures were obtained using *autodE* (v. 1.0.0b3),[8] utilising GFN2-xTB[9] with the GBSA[10] solvent model for MeCN as *lmethod*, and CPCM[11](MeCN)-PBE0-D3BJ[12]/def2-TZVP[13]//CPCM(MeCN)-PBE0-D3BJ/def2-SVP as *hmethod*. Stationary points for the reaction profile were reoptimised at the SMD[14](DMA)-B2GP-PLYP[15]-D3BJ/def2-TZVP level of theory, which was shown to perform well for reaction involving [1.1.1]propellane.[16] Redox calculations were carried out at the SMD(DMA)-M06-2X[17]/def2-TZVP//SMD(DMA)-PBE0-D3BJ/def2-SVP level of theory, which previous benchmark studies by Isegawa *et al.* have shown to provide values to within ~0.1 V of experiment for organic molecules.[18] Thermal corrections were applied using the *otherm* program[19] at 298.15 K and 1 M, and concentrations were adjusted to their experimental ratios ([1.1.1]propellane : amine : DMA : Et_2O = 1 : 10 : 21.6 : 12) using equation S4, where G_{1M} is the free energy of the species at a 1 M concentration, R is the gas constant (1.987×10^{-3} kcal mol^{-1} K^{-1}), T is the temperature (298.15 K), and x corresponds to the actual concentration of the species in the experiment.

$$G_{rel\_conc=xM} = G_{1M} + RT\ln x \qquad \text{(Eq S4)}$$

Estimates of the relative stability of radicals derived from nicotine **6** were carried out using the Gaussian 16 software package (Revision C.01),[20] utilising free energies (298.15 K, 1 atm) obtained at the M06-2X-D3/def2-TZVP level of theory. Thermal Gibbs corrections were computed at the same level, at the selected temperature. Solvent effects were estimated by the Polarization Continuum Model (PCM)[21] method within the Self-Consistent Reaction Field (SCRF)[22] approach. All SCRF-PCM calculations were performed using *N*,*N*-dimethylacetamide (DMA) as model solvent.

Redox potential calculations

The calculated oxidation process for amine **3a** is associated with a reduction potential of 0.81 V vs SCE, in excellent agreement with the experimental range of 0.74–0.88 V vs SCE[23] for this substrate class (Figure S7). However, while the calculated reduction potential of BCP radical **7** was –1.35 V vs SCE, a test calculation on tertiary radical **A** using the same methodology revealed a reduction potential of –0.97 V vs SCE – a 0.31 V error compared with the experimental value of –0.66 ± 0.07 V vs SCE.[24] As a result, we added this error to our calculated reduction potential of **7** to correct the value in line with experiment, giving a final value of E^{o}_{corr} = –1.04 V vs SCE (Figure S7). The same procedure was applied to estimate the reduction potential of iminium ion **14**, where the experimental / calculated values for the related compound **B** are –1.11 / –1.94 V vs SCE,[25] respectively. The

difference between these values (0.83 V) was used to adjust the calculated reduction potential of **8** (–1.16 V) to its corrected value: E^{o}_{corr} = –0.33 V vs SCE (Figure S7).

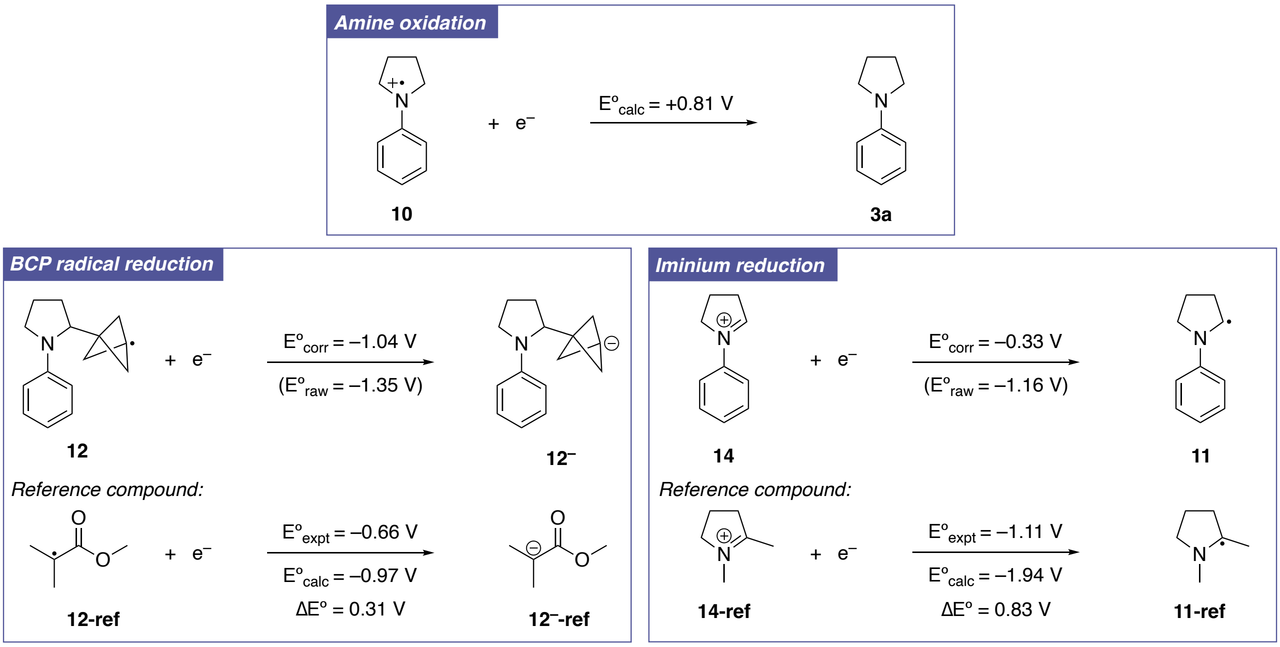

Figure S7. Redox potential estimation procedure, with potentials (in V) calculated at the SMD(DMA)-M06-2X/def2-TZVP//SMD(DMA)-PBE0-D3BJ/def2-SVP level of theory.

Kinetic isotope effect calculations

The kinetic isotope effect (KIE) for the HAT step between 3-methylBCP radical and amine **3a** can be estimated from the difference in activation free energies ($\Delta\Delta G^{\ddagger} = \Delta G^{\ddagger}_{H} - \Delta G^{\ddagger}_{D}$). The value of $\Delta\Delta G^{\ddagger}$ was calculated to be –1.07 kcal mol^{-1}, and using the equation $k_H / k_D = \exp(-\Delta\Delta G^{\ddagger}/RT)$ with T = 298.15 K, the KIE was estimated as 6.1 (Table S5), neglecting tunnelling and anharmonicity effects.

Table S5. Computed electronic and zero-point activation energies (kcal mol^{-1}) HAT from amine **3a** by Me-BCP•, at the SMD(DMA)-B2GP-PLYP-D3BJ/def2-TZVP level. Thermal corrections applied at a temperature of 298.15 K.

Reaction	$\Delta E_{el}^{\ddagger}$	$\Delta ZPE^{\ddagger}$	$\Delta H^{\ddagger}$	$T\Delta S^{\ddagger}$	$\Delta G^{\ddagger}$
H_4-amine 3**a** + Me-BCP• → TS Me-BCP_radical_h4-amine (**3a**)	7.8	-2.7	5.2	-9.3	13.1
D_4-amine 3**a** + Me-BCP• → TS Me-BCP_radical_d4-amine (**3a**)	7.8	-1.8	6.2	-9.4	14.2

Radical stability study

Firstly, we optimized the structure of nicotine **6** (PCM(DMA)-M06-2X-D3/def2-TZVP). Subsequently, we abstracted a hydrogen atom from the optimized structure at each position of the pyrrolidine ring (Figure S8) to generate the corresponding radical.

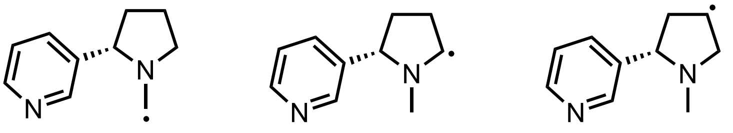

	**6-rad1**	**6-rad2**	**6-rad3**
ΔG_{rel} / kcal mol^{-1}	11.6	10.8	16.8

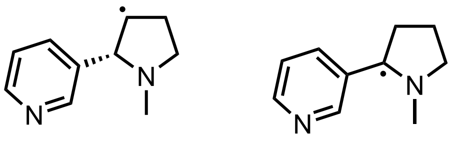

**6-rad4**	**6-rad5**
16.9	0.0

Figure S8. Relative stability (ΔG_{rel}, in kcal mol^{-1} at 298.15 K) of radicals derived from the pyrrolidine ring of nicotine **6**, calculated at the PCM(DMA)-M06-2X-D3/def2-TZVP level of theory.

Computational data

Table S6. Computed energy differences (kcal mol[-1]) for the computational HAT study, at the SMD(DMA)-B2GP-PLYP-D3BJ/def2-TZVP level.

Reaction	ΔEel	ΔZPE	ΔH	TΔqh-S	ΔTotal corr	Δqh-G (1 M)	Δqh-G (x M)
h_4-amine 3**a** + Me-BCP• → TS Me-BCP_radical_h_4-amine (**3a**)	7.8	-2.7	5.2	-9.3	6.7	14.5	13.1
h_4-amine **3a** + Me-BCP• → amino• **11** + Me-BCP-H	-14.3	-0.1	-14.6	-0.1	-0.3	-14.6	-15.9
d_4-amine 3**a** + Me-BCP• → TS Me-BCP_radical_d_4-amine (**3a**)	7.8	-1.8	6.2	-9.4	7.7	15.5	14.2
dimethylaniline **3u** + Me-BCP• → TS Me-BCP_radical_dimethylaniline (**3u**)	9.2	-2.8	6.5	-9.4	6.7	15.9	14.5
dimethylaniline **3u** + Me-BCP• → dimethylaniline• + Me-BCP-H	-13.2	-0.5	-13.8	-0.3	-0.3	-13.5	-14.9
DMA + Me-BCP• → TS Me-BCP_radical_DMA-H1	10.7	-2.6	8.0	-9.3	6.6	17.3	15.5
DMA + Me-BCP• → DMA_H1• + Me-BCP-H	-4.7	-0.4	-5.4	-0.4	-0.3	-5.1	-6.9
DMA + Me-BCP• → TS Me-BCP_radical_DMA-H2	10.4	-2.4	7.7	-9.6	6.9	17.3	15.4
DMA + Me-BCP• → DMA_H2• + Me-BCP-H	-11.2	-0.9	-12.2	-0.4	-0.6	-11.8	-13.6
Et_2O + Me-BCP• → TS Me-BCP_radical_Et_2O	10.8	-3.0	8.0	-8.3	5.5	16.3	14.8
Et_2O + Me-BCP• → Et_2O• + Me-BCP-H	-9.4	-0.8	-10.1	0.6	-1.3	-10.7	-12.2
TCP + Me-BCP• → TS Me-BCP_radical_staffane	5.3	0.0	5.5	-7.1	7.3	12.6	12.6
TCP + Me-BCP• → staffane•	-34.2	2.5	-32.8	-11.2	12.6	-21.6	-21.6

Table S7. Computed energies (Ha) and TS imaginary frequencies (cm^{-1}) for the computational HAT study, at the SMD(DMA)-B2GP-PLYP-D3BJ/def2-TZVP level.

System	E_{el}	ZPE	H	Tqh-S	Total corr	qh-G (1 M)	qh-G (x M)	TS v_{imag}
h_4-amine **3a**	-443.32623	0.21119	-443.10455	0.04173	0.17995	-443.14627	-443.14410	-
d_4-amine **3a**	-443.32623	0.19776	-443.11758	0.04243	0.16622	-443.16001	-443.15784	-
dimethylaniline **3u**	-365.95385	0.17466	-365.76967	0.03989	0.14429	-365.80956	-365.80738	-
DMA	-287.65233	0.13087	-287.51243	0.03791	0.10199	-287.55034	-287.54744	-
Et_2O	-233.49922	0.13750	-233.35390	0.03446	0.11086	-233.38836	-233.38601	-
TCP	-193.87472	0.09392	-193.77588	0.02650	0.07234	-193.80237	-193.80237	-
Me-BCP radical	-233.72087	0.13286	-233.58159	0.03072	0.10856	-233.61231	-233.61231	-
TS Me-BCP_radical_h_4-amine (**3a**)	-677.03465	0.33970	-676.67784	0.05768	0.29912	-676.73552	-676.73552	-1561
TS Me-BCP_radical_d_4-amine (**3a**)	-677.03465	0.32782	-676.68931	0.05824	0.28709	-676.74756	-676.74756	-1161
TS Me-BCP_radical_dimethylaniline (**3u**)	-599.66012	0.30305	-599.34090	0.05569	0.26352	-599.39660	-599.39660	-1671
TS Me-BCP_radical_DMA-H1	-521.35619	0.25965	-521.08131	0.05382	0.22106	-521.13513	-521.13513	-1599
TS Me-BCP_radical_DMA-H2	-521.35665	0.25982	-521.08173	0.05341	0.22151	-521.13514	-521.13514	-1724
TS Me-BCP_radical_Et_2O	-467.20286	0.26560	-466.92269	0.05202	0.22815	-466.97470	-466.97470	-1701
TS Me-BCP_radical_staffane	-427.58721	0.22684	-427.34872	0.04590	0.19259	-427.39462	-427.39462	-126
Amino radical **11**	-442.67639	0.19846	-442.46780	0.04149	0.16710	-442.50929	-442.50929	-
Dimethylaniline radical	-365.30227	0.16122	-365.13162	0.03926	0.13139	-365.17088	-365.17088	-
DMA-H1 radical	-286.98724	0.11759	-286.86103	0.03721	0.08899	-286.89824	-286.89824	-
DMA-H2 radical	-286.99752	0.11690	-286.87186	0.03711	0.08855	-286.90897	-286.90897	-
Et_2O radical	-232.84160	0.12370	-232.70997	0.03525	0.09638	-232.74522	-232.74522	-
Staffane radical	-427.65010	0.23070	-427.40976	0.03934	0.20100	-427.44910	-427.44910	-
Me-BCP-H	-234.39351	0.14542	-234.24165	0.03086	0.12100	-234.27250	-234.27250	-

Table S8. Computed energy differences (kcal mol^{-1}) for the computational redox potential study, at the SMD(DMA)-M06-2X/def2-TZVP//SMD(DMA)-PBE0-D3BJ/def2-SVP level.

	SMD(DMA)-PBE0-D3BJ/def2-SVP						SMD(DMA)-M06-2X /def2-TZVP	
**Redox process**	**ΔEel**	**ΔZPE**	**ΔH**	**TΔqh-S**	**ΔTotal corr**	**Δqh-G**	**ΔEel**	**Δqh-G**
amine-BCP radical **12** → anion **12⁻**	-62.8	-1.2	-64.0	0.0	-1.2	-64.0	-66.3	-67.5
amine radical cation **10** → amine **3a**	-112.4	-0.2	-112.5	-0.2	0.0	-112.3	-117.3	-117.3
iminium **14** → amino radical **11**	-71.7	-2.0	-73.5	0.1	-1.9	-73.7	-70.1	-72.0
Me-pyrrolidine iminium **14-ref** → amino radical **11-ref**	-53.5	-2.0	-55.6	-0.7	-1.4	-54.9	-52.6	-54.1
$CHMe_2CO_2Me$ radical **12-ref** → anion **12⁻-ref**	-69.9	-1.4	-71.2	0.4	-1.6	-71.6	-74.3	-76.0

Table S9. Computed energies (Ha) for the computational redox potential study, at the SMD(DMA)-M06-2X/def2-TZVP//SMD(DMA)-PBE0-D3BJ/def2-SVP level.

	SMD(DMA)-PBE0-D3BJ/def2-SVP						SMD(DMA)-M06-2X /def2-TZVP	
**System**	**Eel**	**ZPE**	**H**	**Tqh-S**	**Total corr**	**qh-G**	**Eel**	**qh-G**
amine-BCP radical **12**	-636.55738	0.29613	-636.24710	0.05078	0.25950	-636.29788	-637.00374	-636.74424
amine-BCP anion **12⁻**	-636.65744	0.29416	-636.34912	0.05077	0.25754	-636.39989	-637.10942	-636.85188
Amine radical cation **10**	-443.12482	0.21100	-442.90368	0.04167	0.17947	-442.94535	-443.42774	-443.24827
Amine **3a**	-443.30387	0.21060	-443.08302	0.04133	0.17952	-443.12435	-443.61466	-443.43514
Iminium **14**	-442.54129	0.20032	-442.33106	0.04137	0.16887	-442.37243	-442.85203	-442.68316
Amino radical **11**	-442.65561	0.19719	-442.44823	0.04160	0.16578	-442.48983	-442.96370	-442.79793
Me-pyrrolidine amino radical **11-ref**	-290.33362	0.17117	-290.15358	0.03763	0.14241	-290.19121	-290.52559	-290.38319
Me-pyrrolidine iminium **14-ref**	-290.24836	0.17435	-290.06498	0.03868	0.14470	-290.10366	-290.44171	-290.29701
$CHMe_2CO_2Me$ radical **12-ref**	-346.12392	0.13257	-345.98148	0.04064	0.10181	-346.02211	-346.35622	-346.25441
$CHMe_2CO_2Me$ anion **12⁻-ref**	-346.23537	0.13032	-346.09491	0.04124	0.09922	-346.13615	-346.47467	-346.37545

Table S10. Computed energies (Ha) and relative energies (kcal mol^{-1}) corresponding to radicals derived from the pyrrolidine ring of **6** computed at the PCM(DMA)-M06-2X-D3/def2-TZVP level of theory. Thermal corrections applied for a temperature of 298.15 K.

Nicotine radical	Eel	ZPE	H	TS	G	ΔEel	ΔZPE	ΔH	TΔS	ΔG
**6-rad1**	-498.29808	0.21417	-498.07227	0.04957	-498.12184	12.8	-0.7	12.2	0.6	11.6
**6-rad2**	-498.29922	0.21383	-498.07362	0.04950	-498.12312	12.1	-0.9	11.4	0.6	10.8
**6-rad3**	-498.28850	0.21278	-498.06394	0.04960	-498.11354	18.8	-1.5	17.4	0.7	16.8
**6-rad4**	-498.28871	0.21305	-498.06396	0.04934	-498.11330	18.7	-1.4	17.4	0.5	16.9
**6-rad5**	-498.31854	0.21525	-498.09175	0.04854	-498.14029	0	0.0	0	0.0	0

12. NMR Spectra

2-(bicyclo[1.1.1]pentan-1-yl)-1-phenylpyrrolidine, 3a and 4a

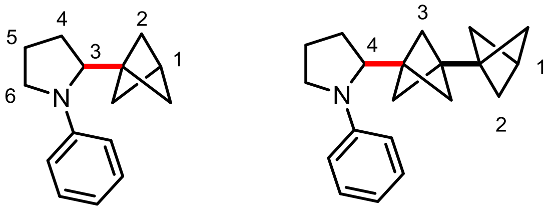

^{1}H NMR (400 MHz, $CDCl_3$)

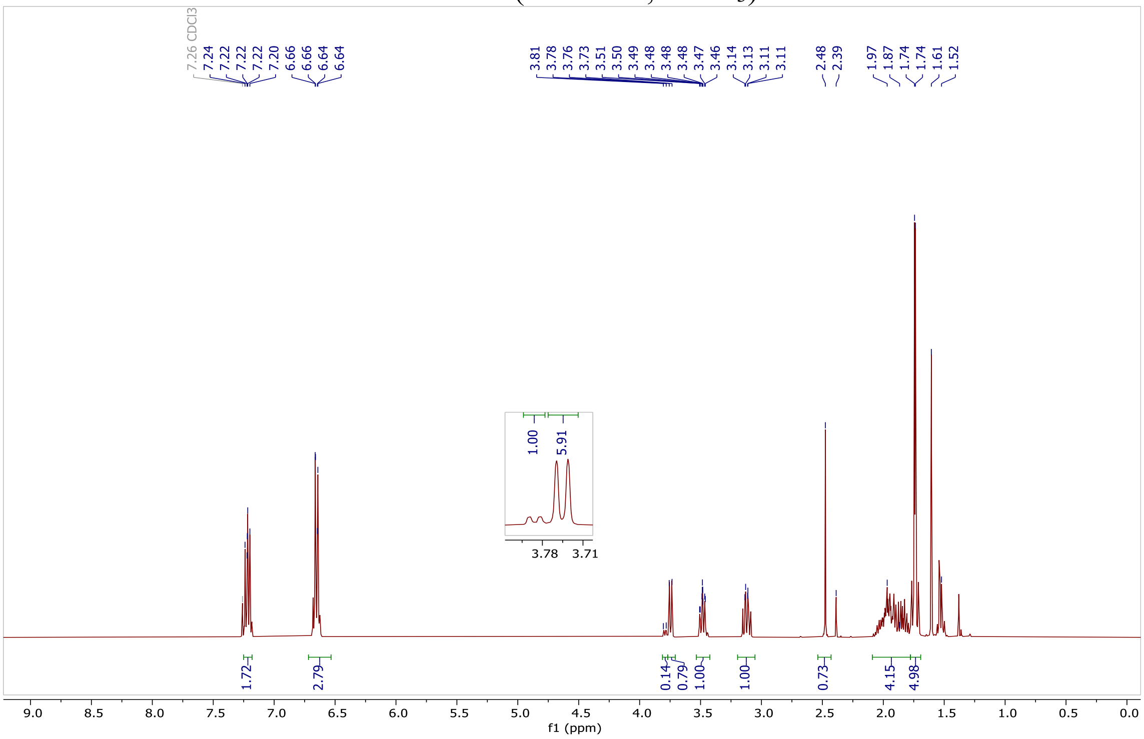

^{13}C NMR (101 MHz, $CDCl_3$)

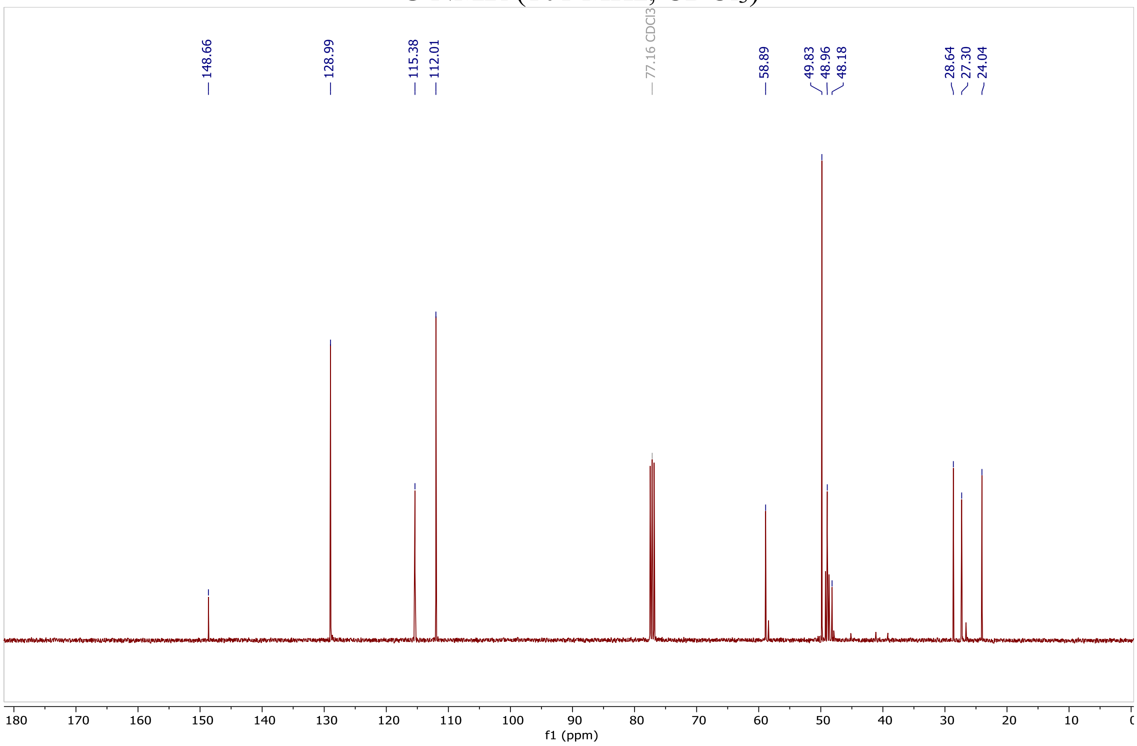

2-(bicyclo[1.1.1]pentan-1-yl)-1-(4-(methylsulfonyl)phenyl)pyrrolidine, 3b

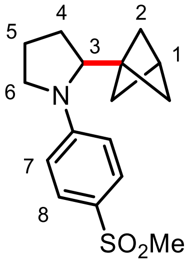

^{1}H NMR (400 MHz, $CDCl_3$)

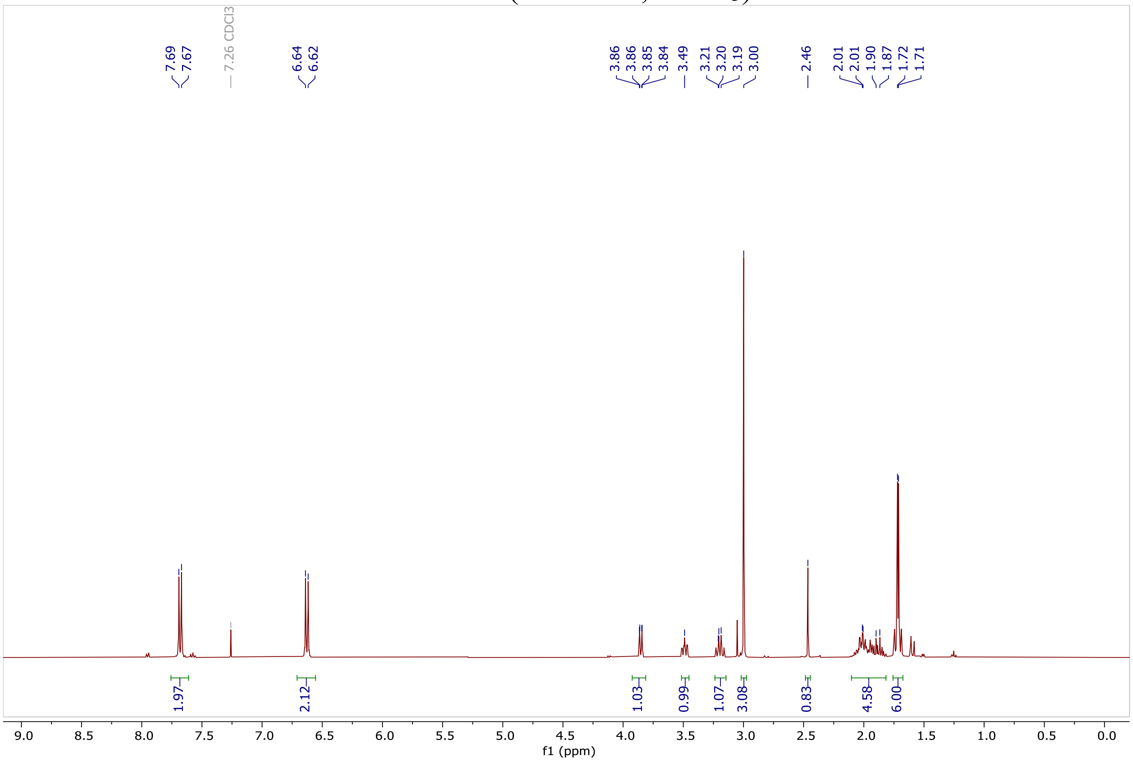

^{13}C NMR (101 MHz, $CDCl_3$)

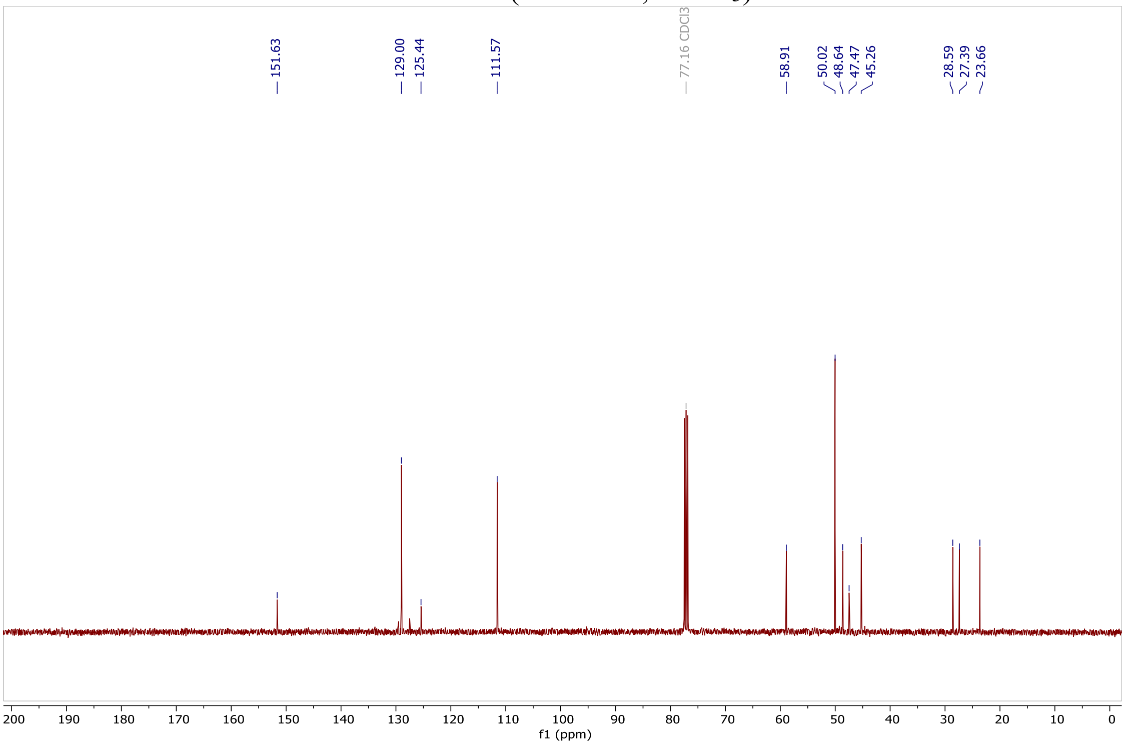

Methyl-4-(2-(bicyclo[1.1.1]pentan-1-yl)pyrrolidin-1-yl)benzoate, 3c

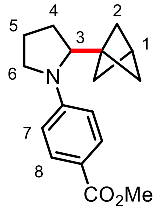

^{1}H NMR (400 MHz, $CDCl_3$)

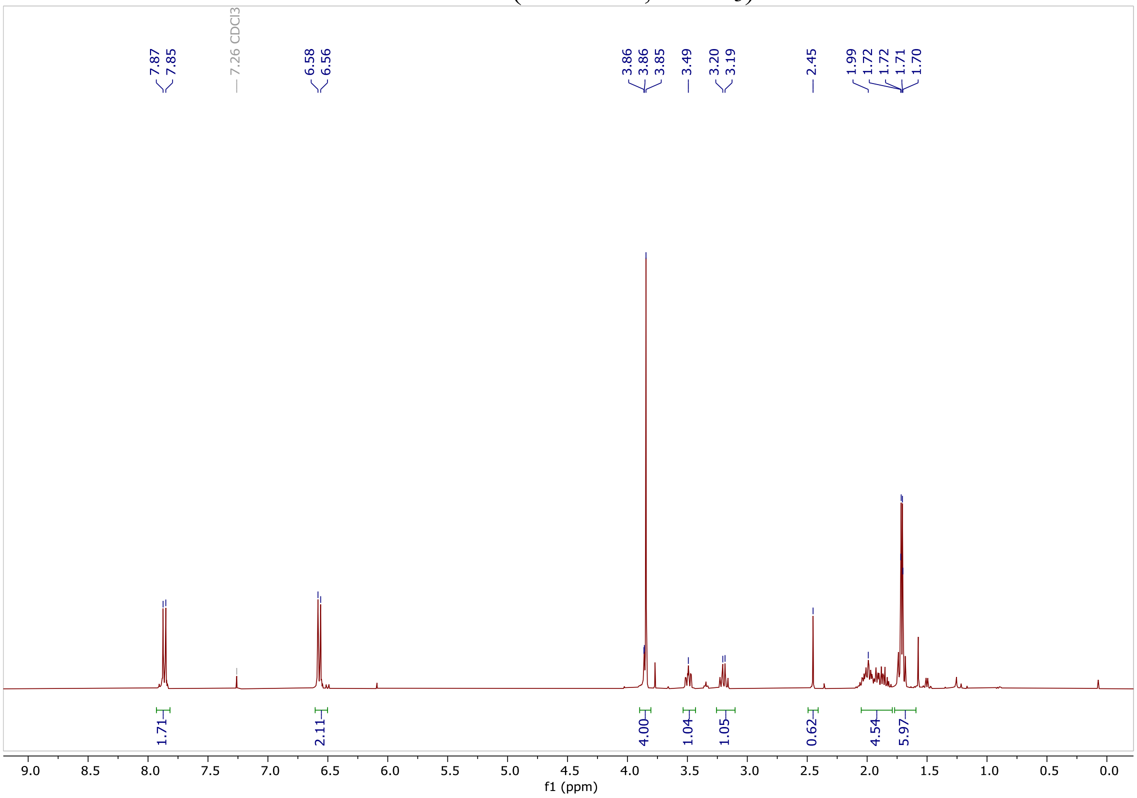

^{13}C NMR (101 MHz, $CDCl_3$)

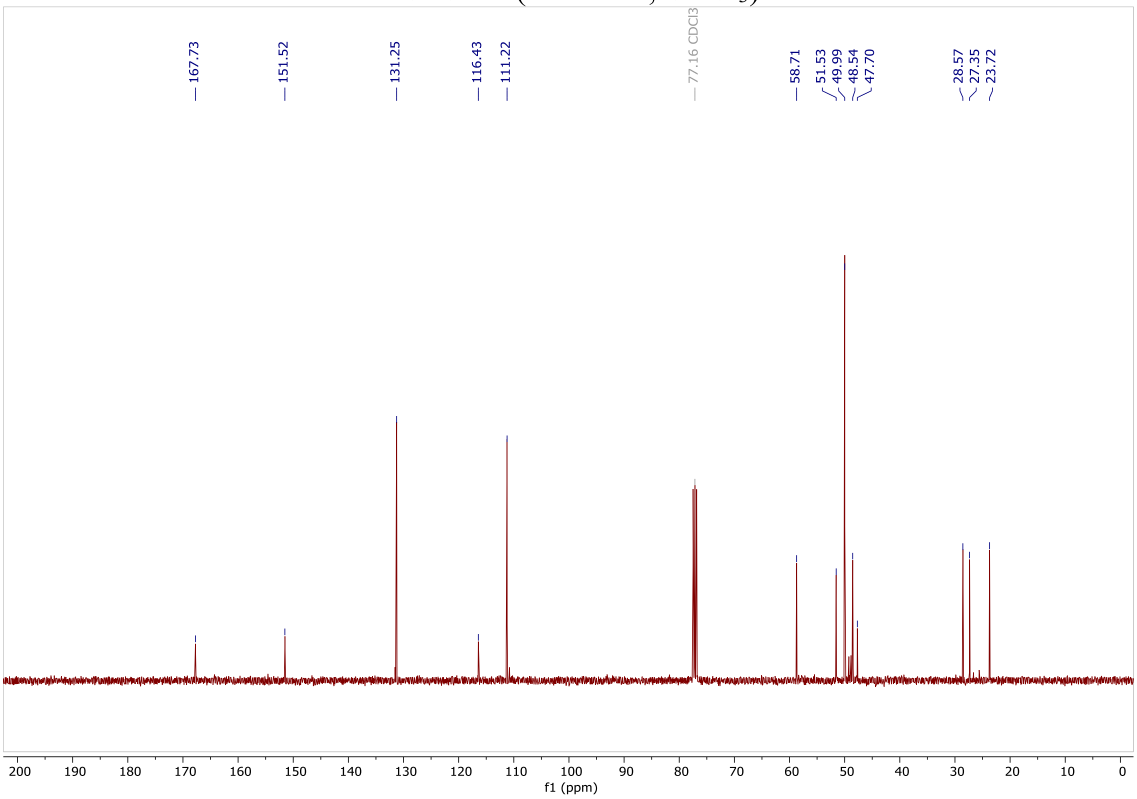

4-(2-(bicyclo[1.1.1]pentan-1-yl)pyrrolidin-1-yl)benzonitrile, 3d and 4d

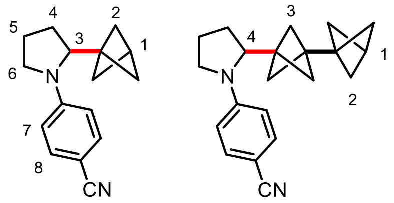

^{1}H NMR (400 MHz, $CDCl_3$)

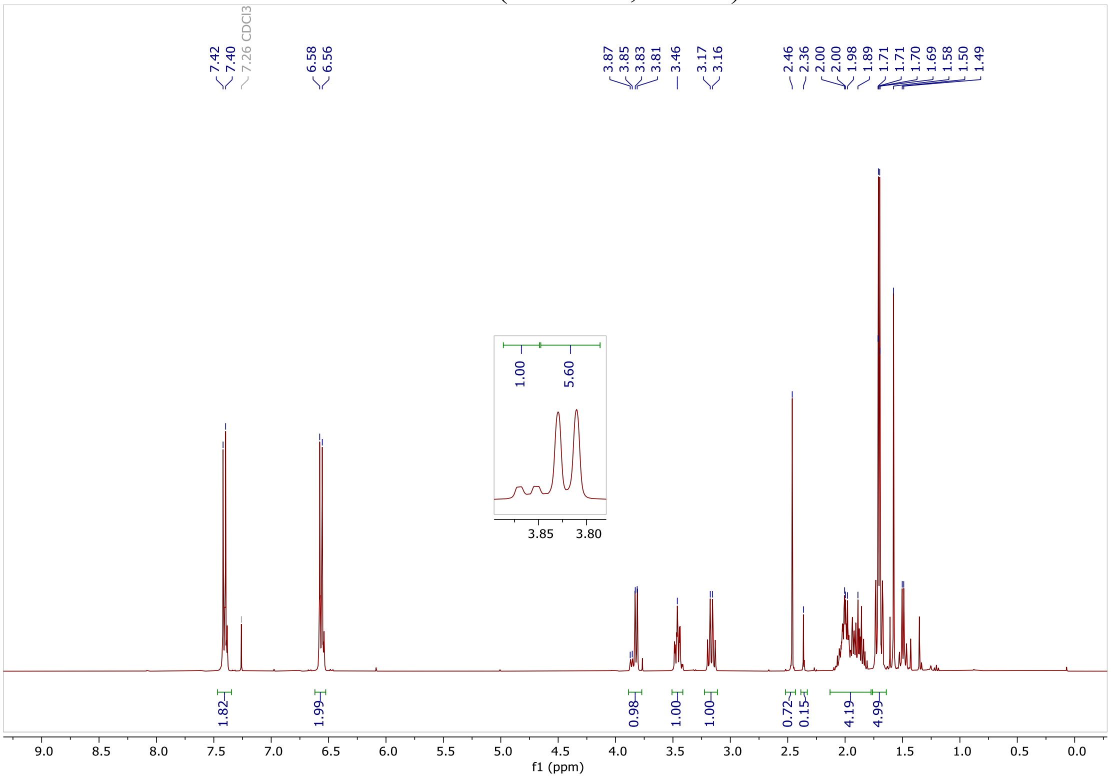

^{13}C NMR (101 MHz, $CDCl_3$)

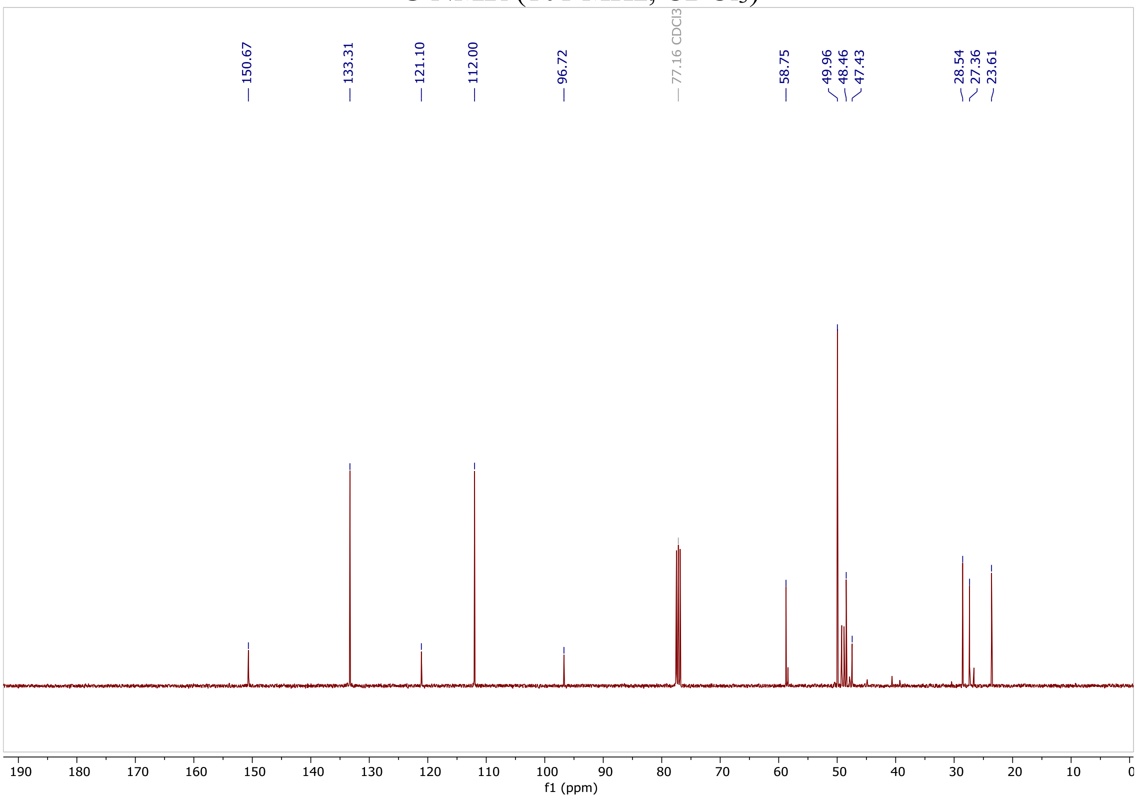

2-(bicyclo[1.1.1]pentan-1-yl)-1-(4-fluorophenyl)pyrrolidine, 3e and 4e

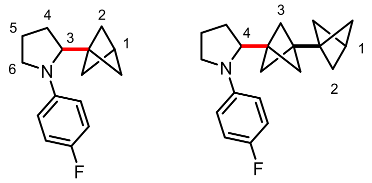

^{1}H NMR (400 MHz, CDCl$_3$)

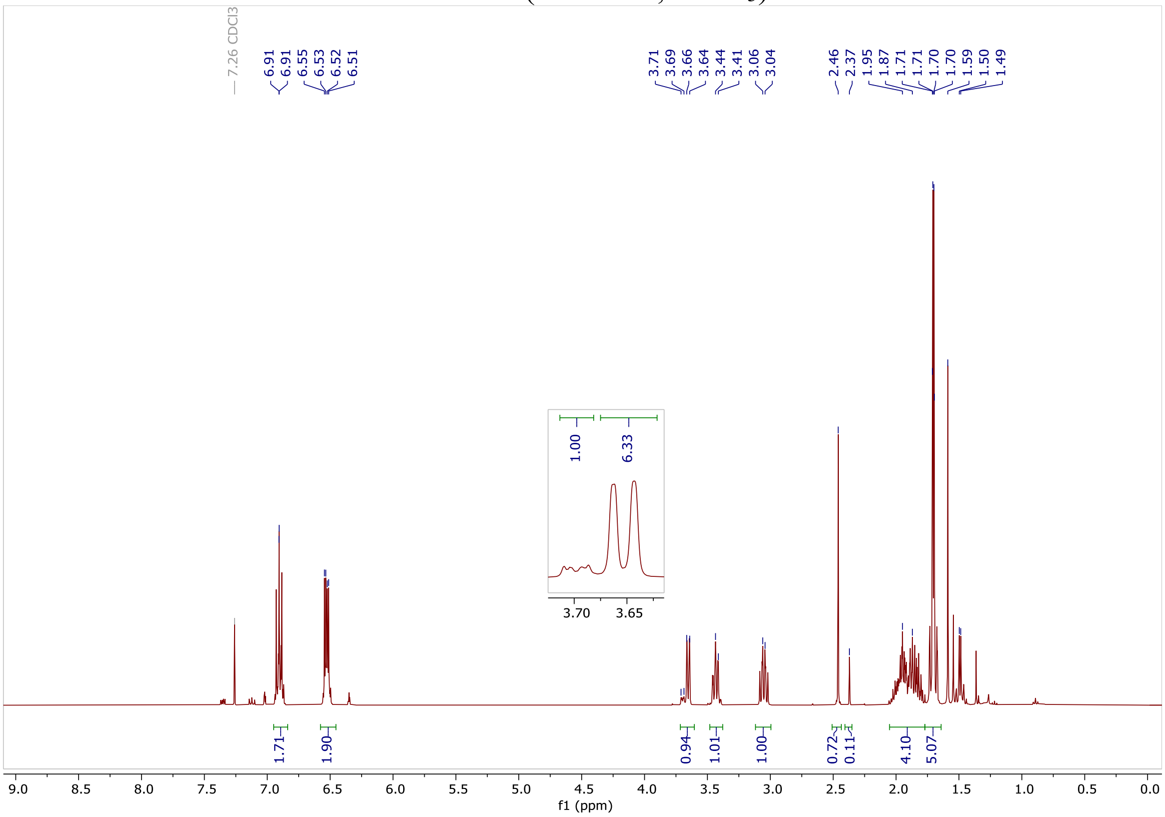

^{13}C NMR (101 MHz, CDCl$_3$)

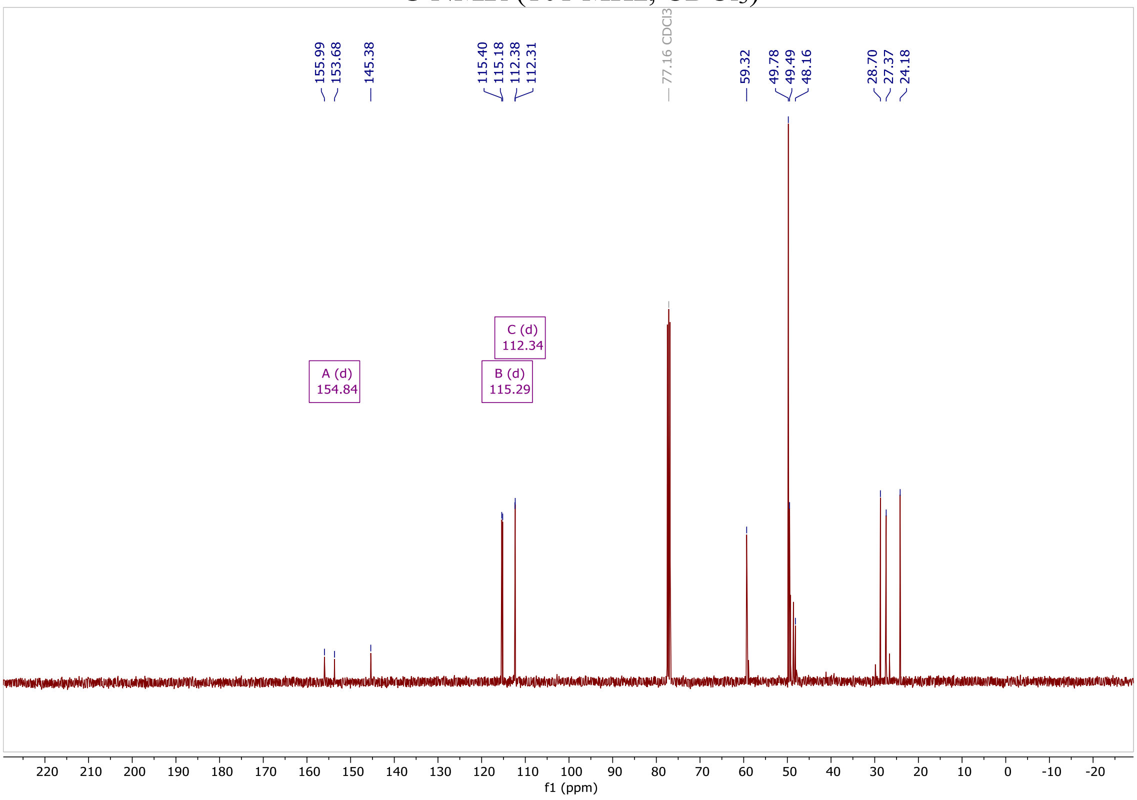

^{19}F NMR (377 MHz, $CDCl_3$)

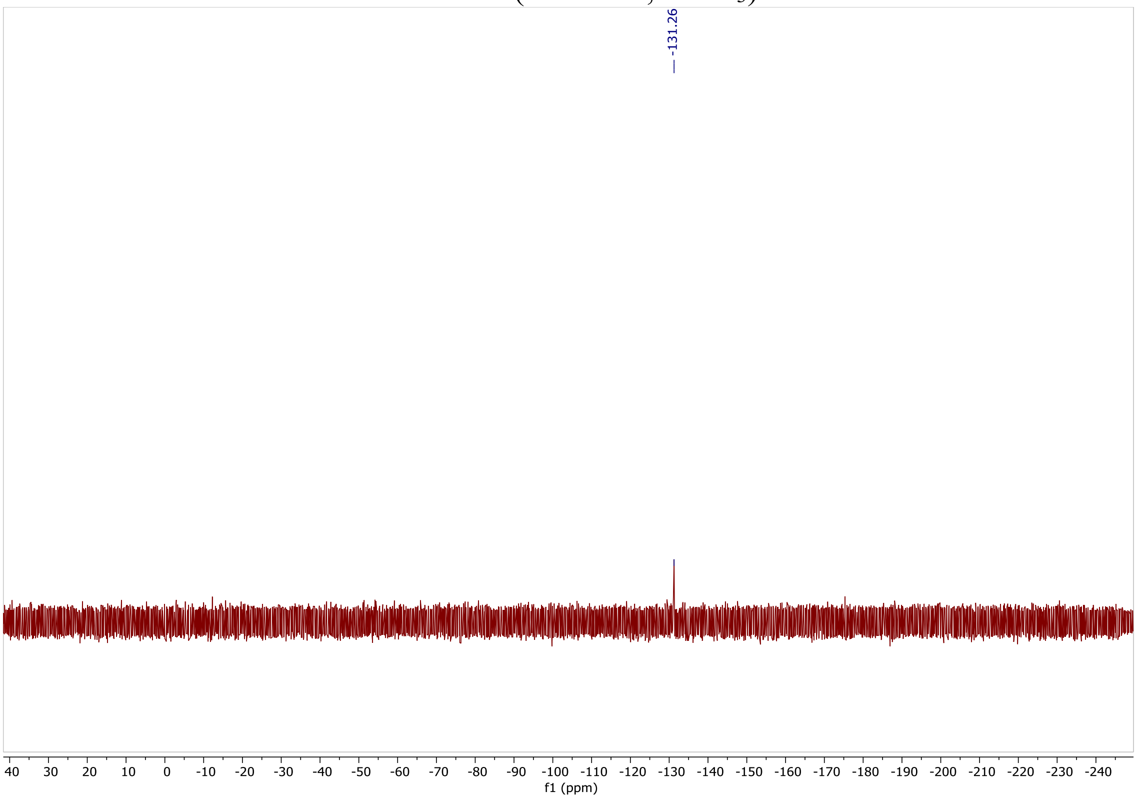

2-(Bicyclo[1.1.1]pentan-1-yl)-1-(4-chlorophenyl)pyrrolidine, 3f and 4f

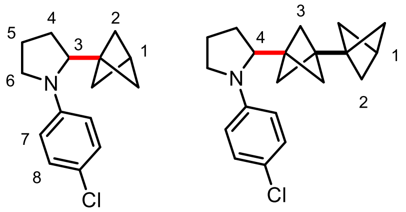

^{1}H NMR (400 MHz, $CDCl_3$)

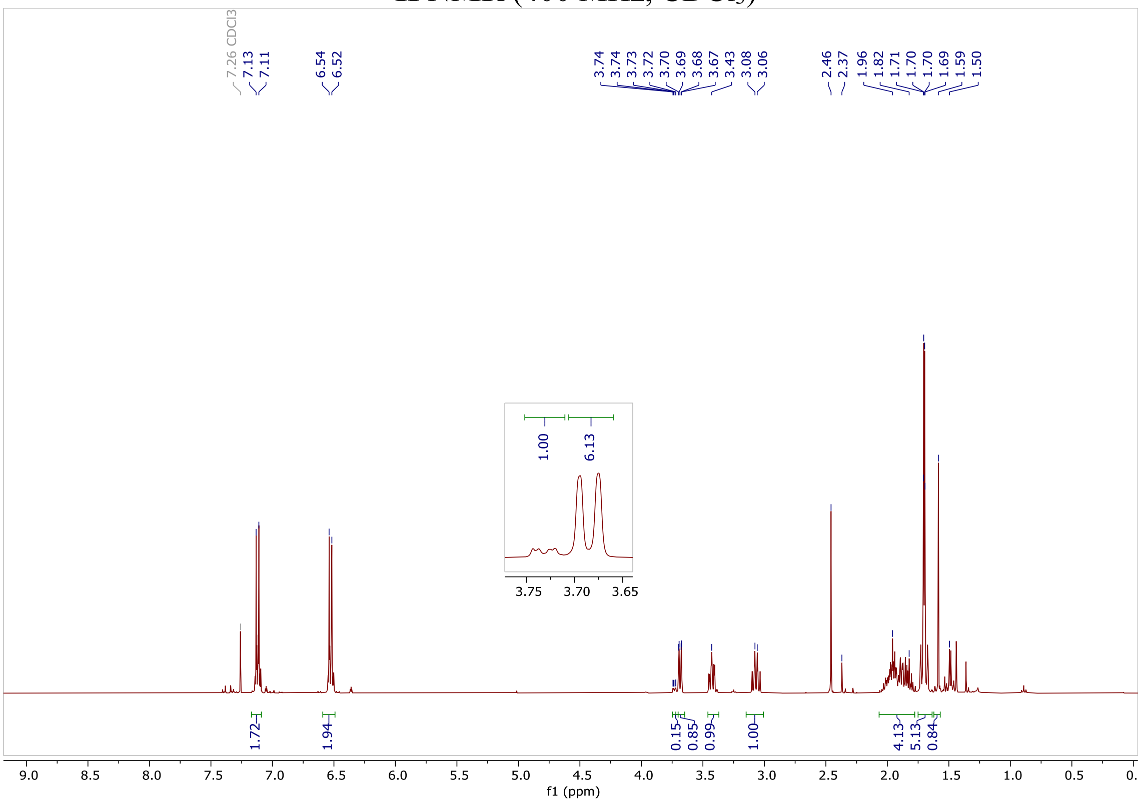

^{13}C NMR (101 MHz, $CDCl_3$)

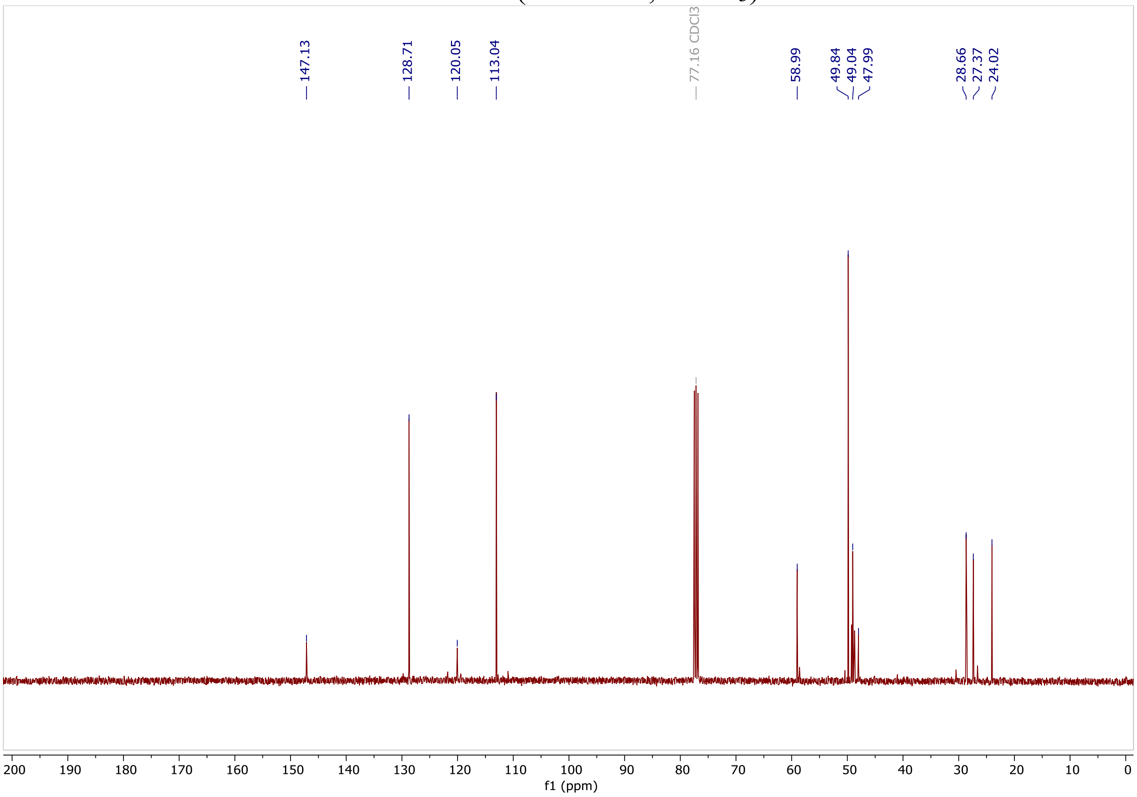

3-(2-(Bicyclo[1.1.1]pentan-1-yl)pyrrolidin-1-yl)benzonitrile, 3g

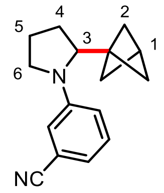

^{1}**H NMR** (400 MHz, $CDCl_3$)

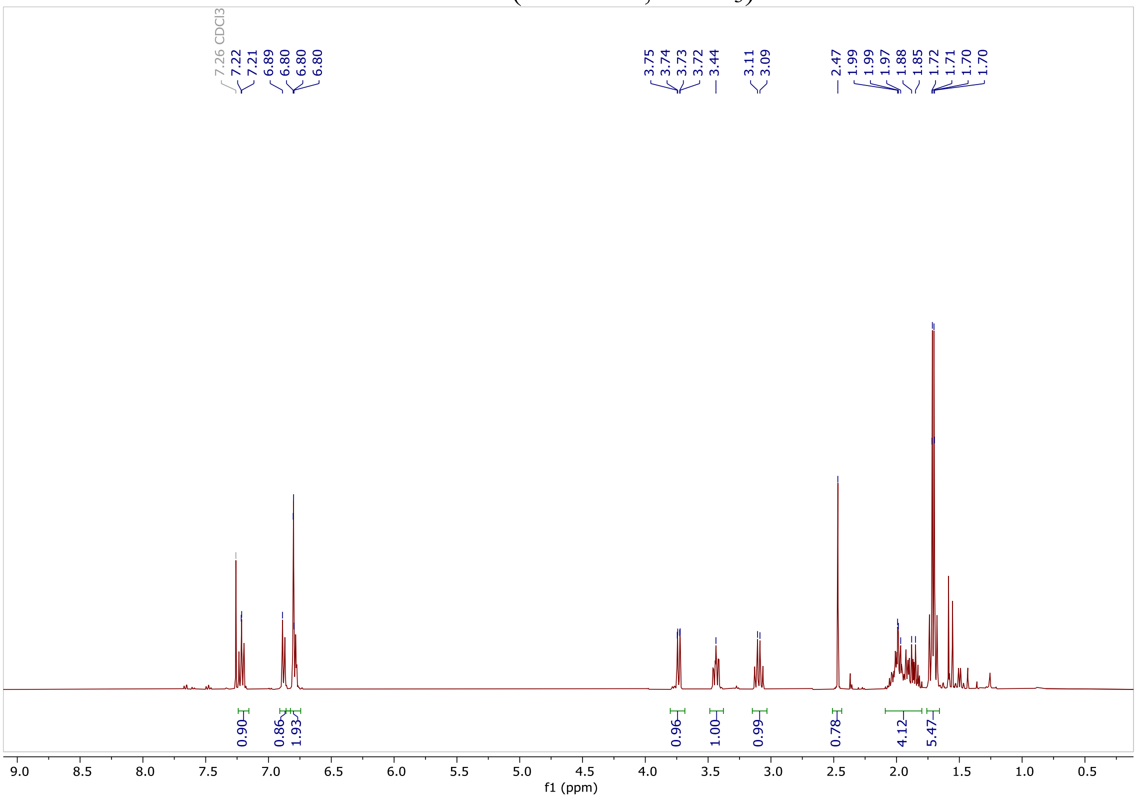

^{13}**C NMR** (101 MHz, $CDCl_3$)

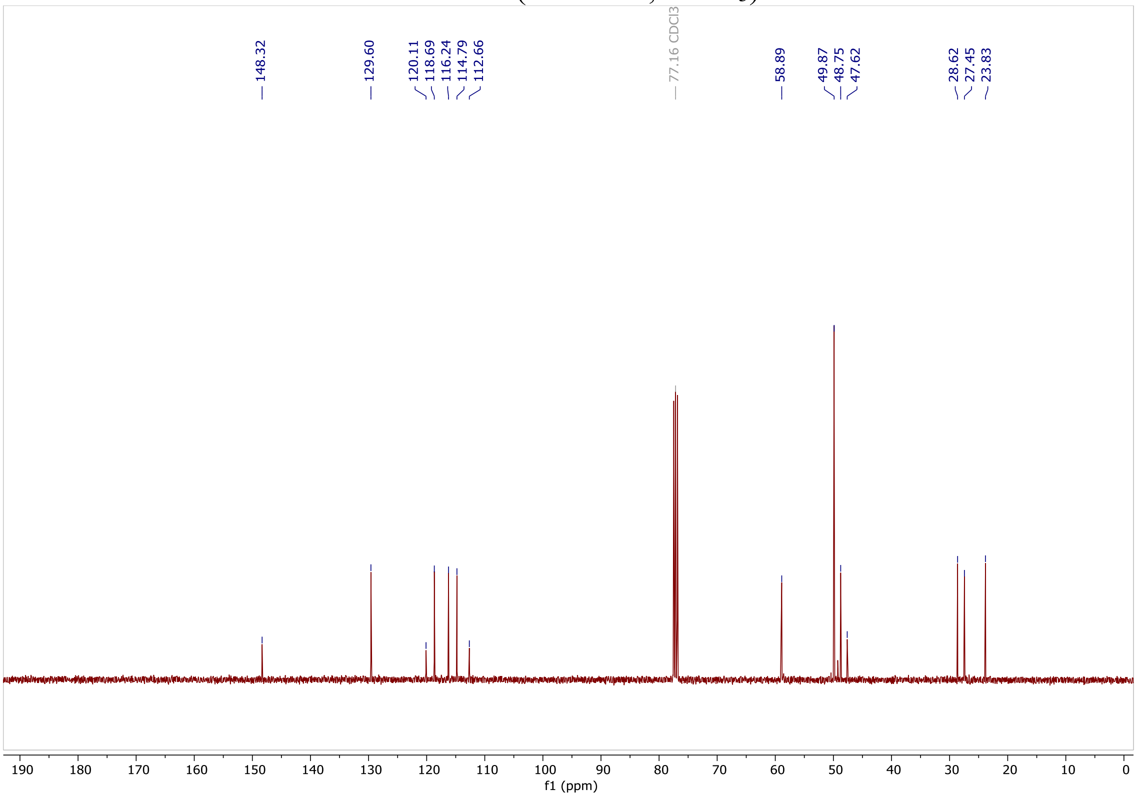

2-(2-(Bicyclo[1.1.1]pentan-1-yl)pyrrolidin-1-yl)benzonitrile, 3h and 4h

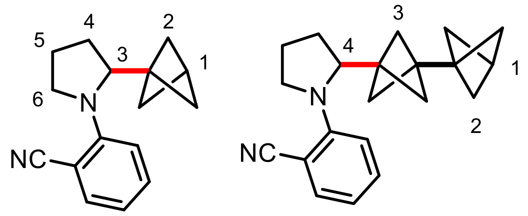

^{1}**H NMR** (400 MHz, CDCl$_3$)

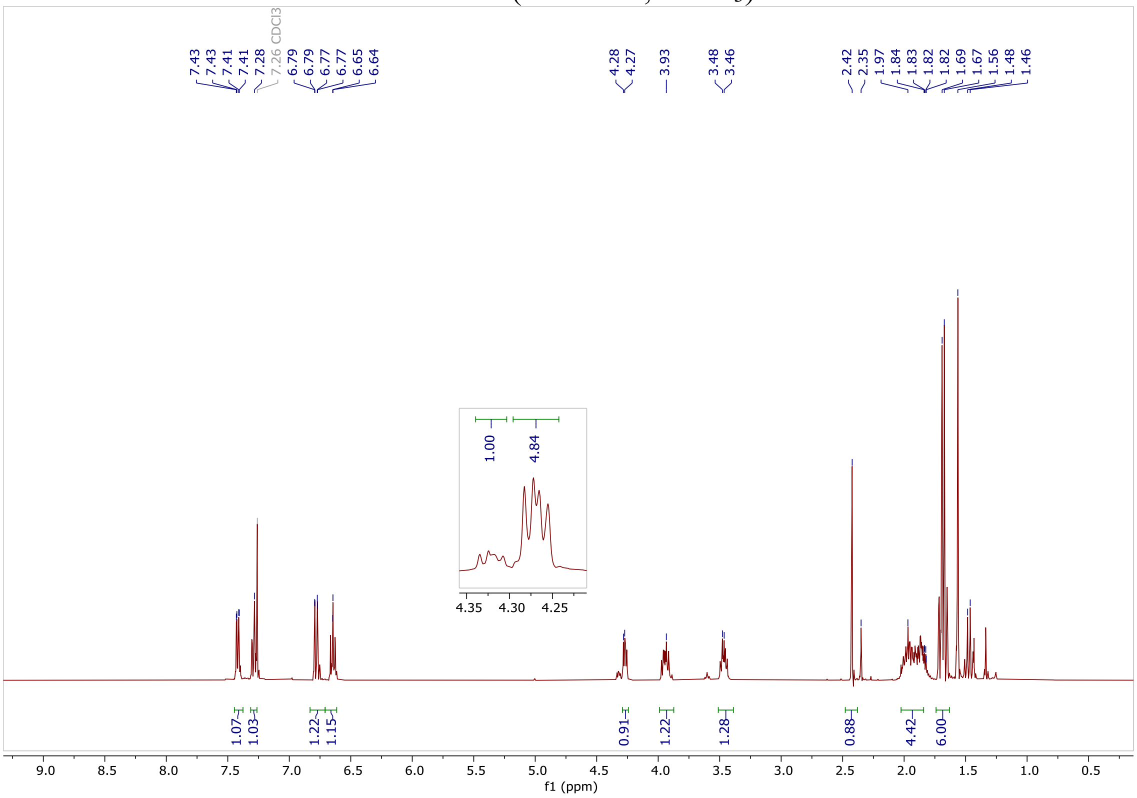

^{13}**C NMR** (101 MHz, CDCl$_3$)

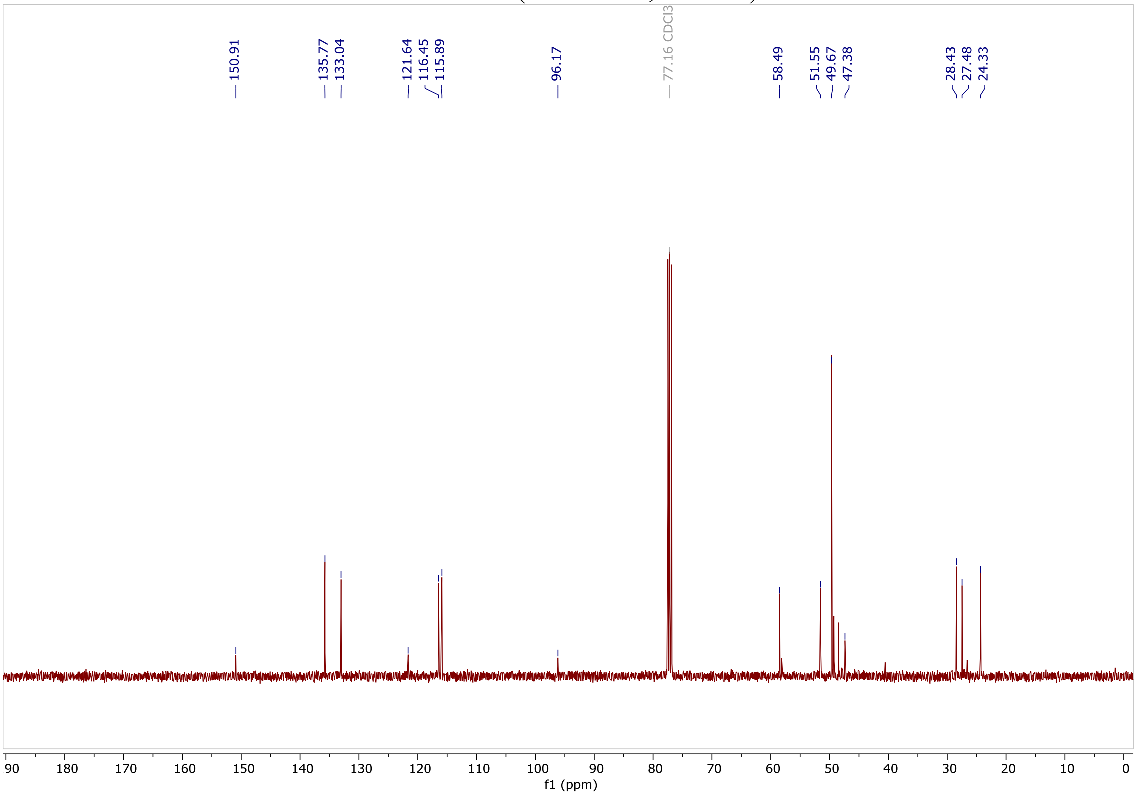

1-(4-(2-(Bicyclo[1.1.1]pentan-1-yl)pyrrolidin-1-yl)-2,5-difluorophenyl)ethan-1-one, 3i

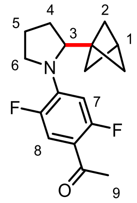

^{1}H NMR (400 MHz, $CDCl_3$)

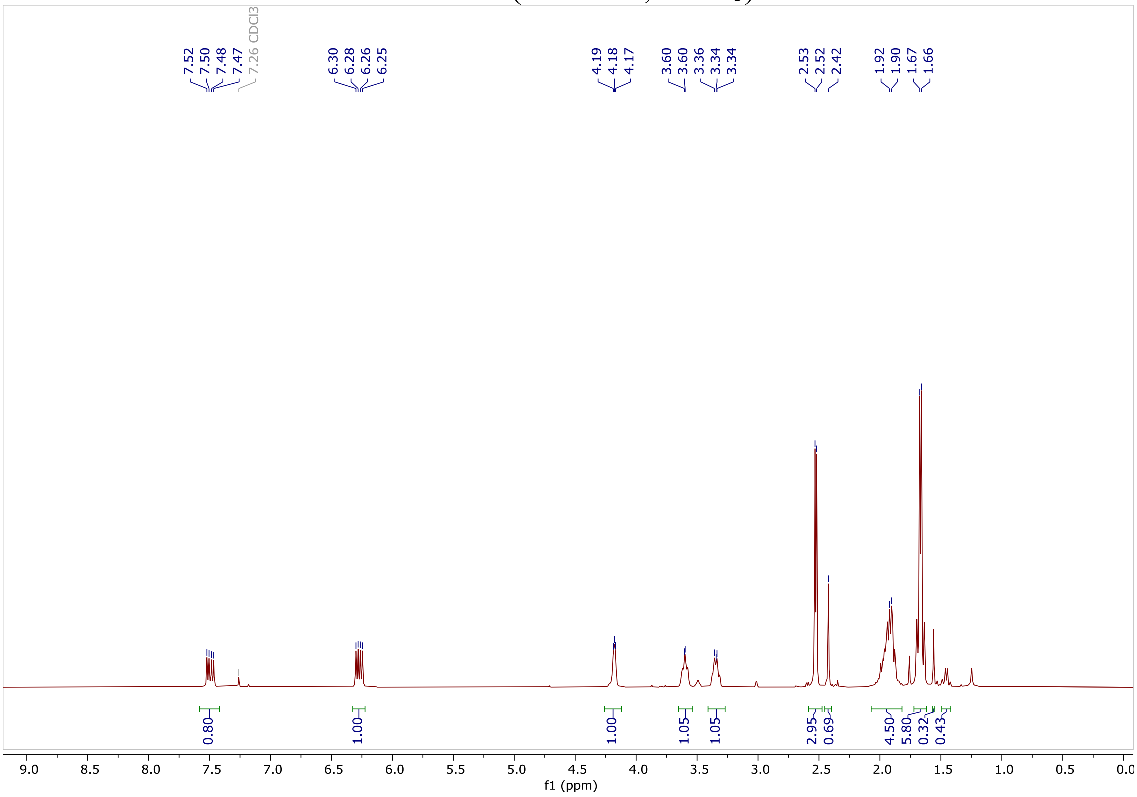

^{13}C NMR (101 MHz, $CDCl_3$)

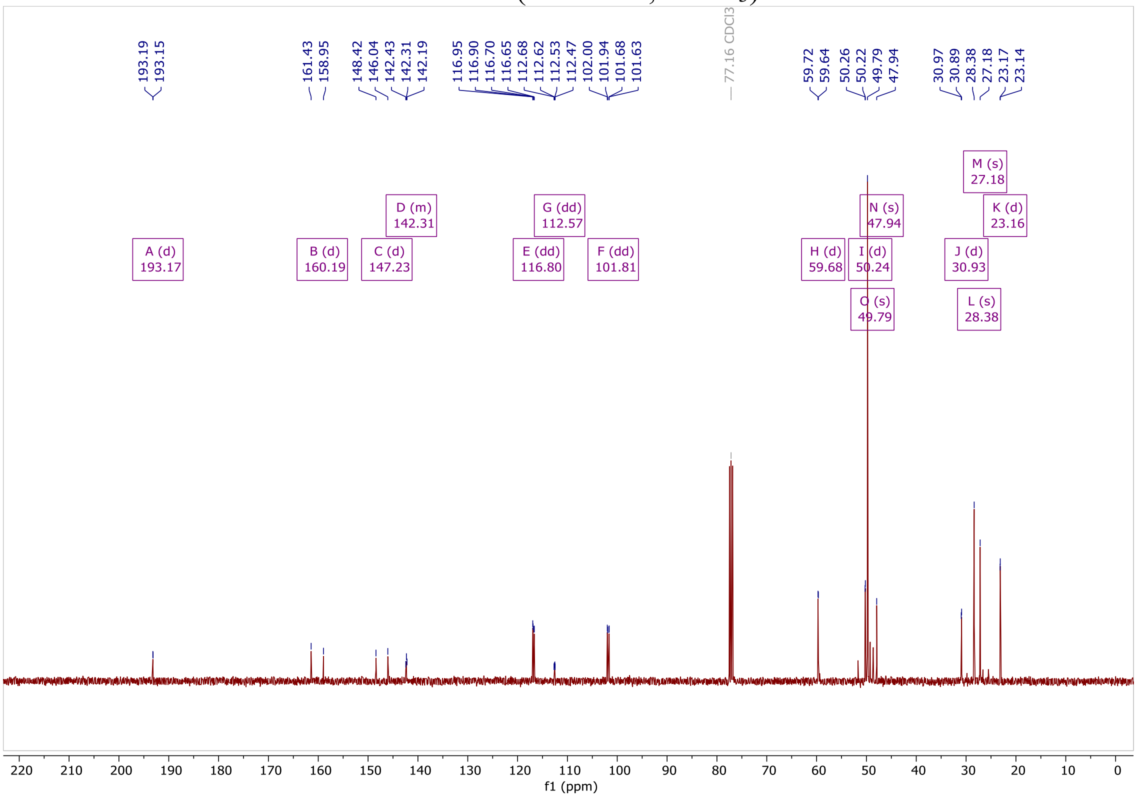

^{19}F NMR (377 MHz, $CDCl_3$)

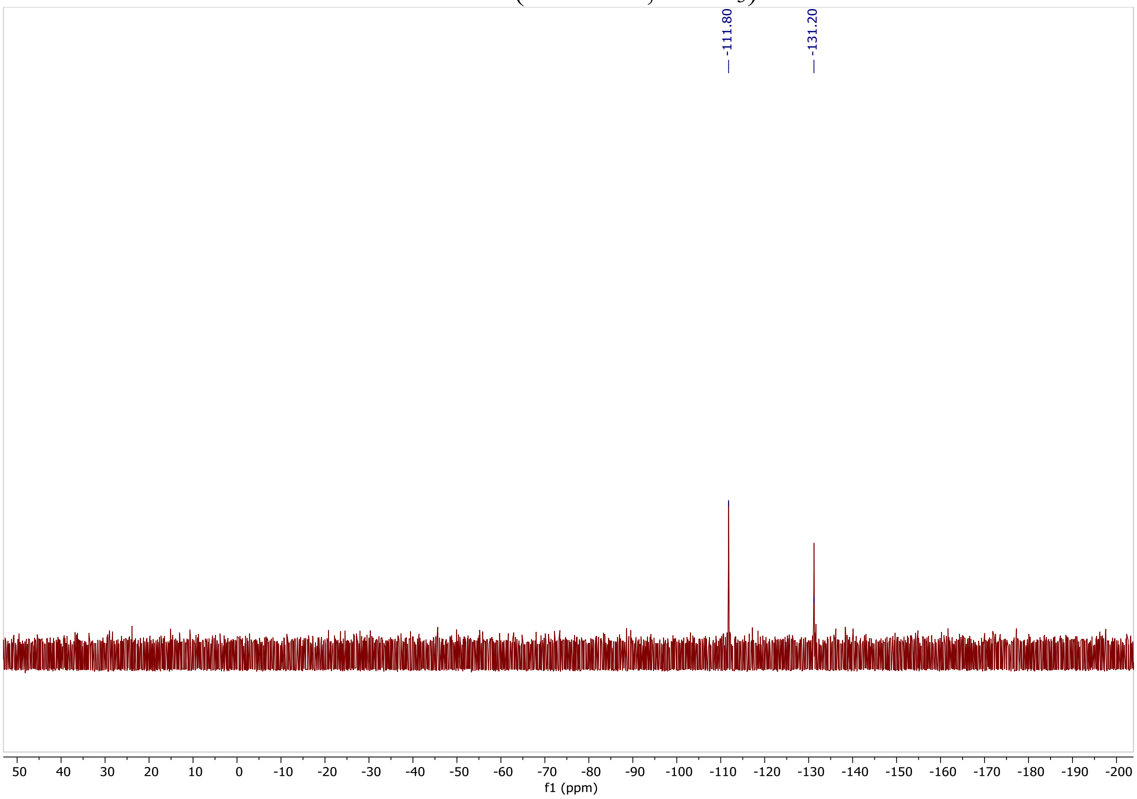

2-(2-(Bicyclo[1.1.1]pentan-1-yl)pyrrolidin-1-yl)pyridine, 3j and 4j

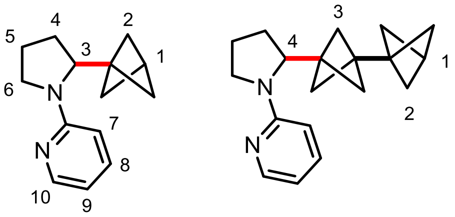

^{1}H NMR (400 MHz, $CDCl_3$)

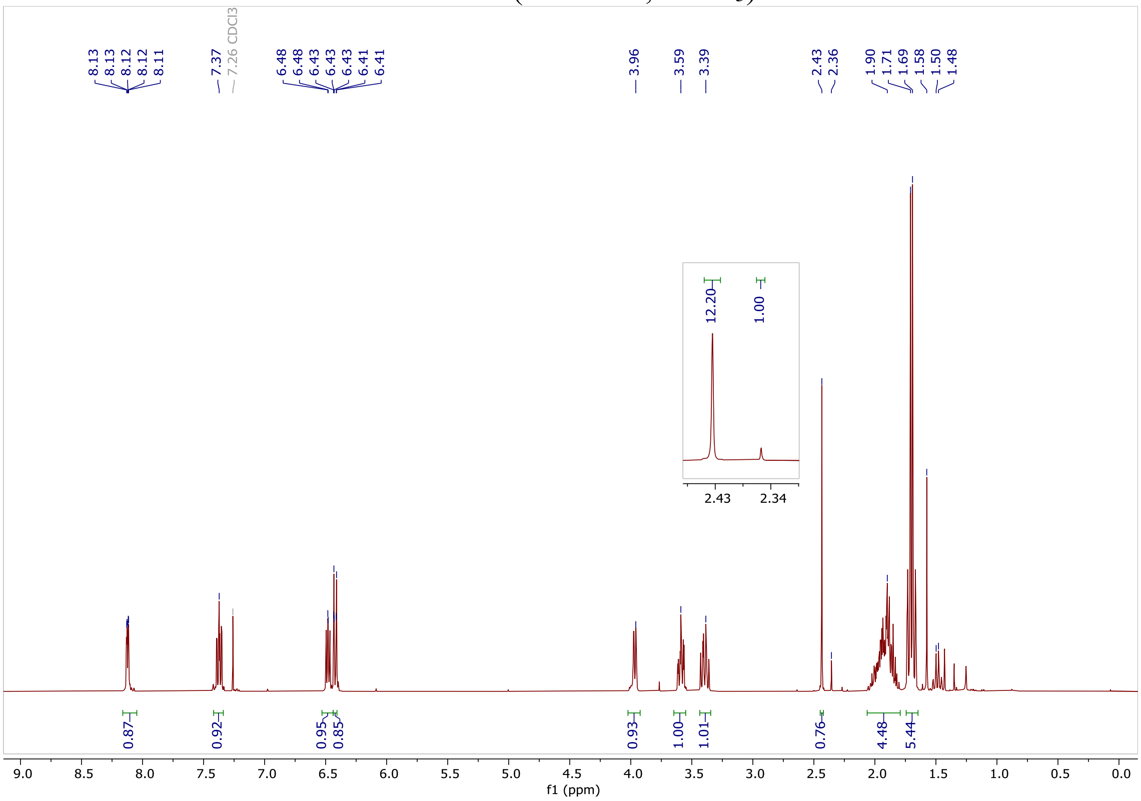

^{13}C NMR (101 MHz, $CDCl_3$)

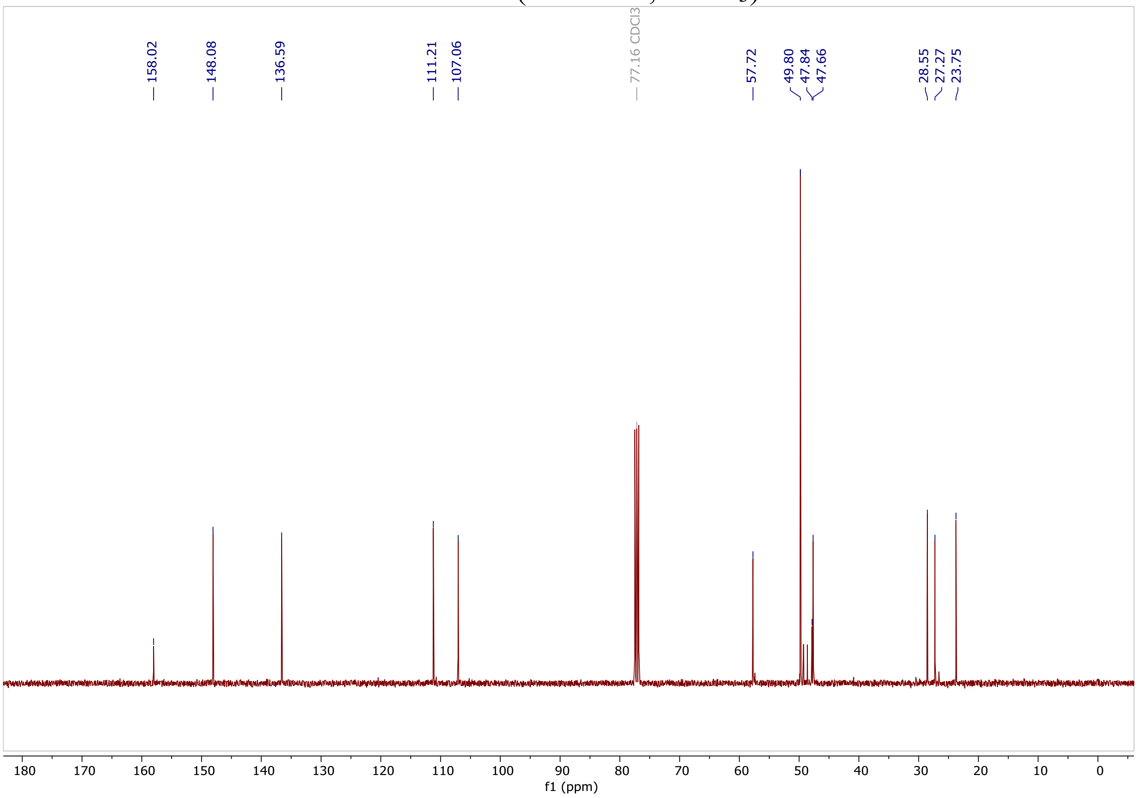

3-(2-(Bicyclo[1.1.1]pentan-1-yl)pyrrolidin-1-yl)pyridine, 3k

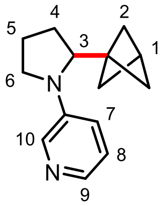

^{1}H NMR (400 MHz, $CDCl_3$)

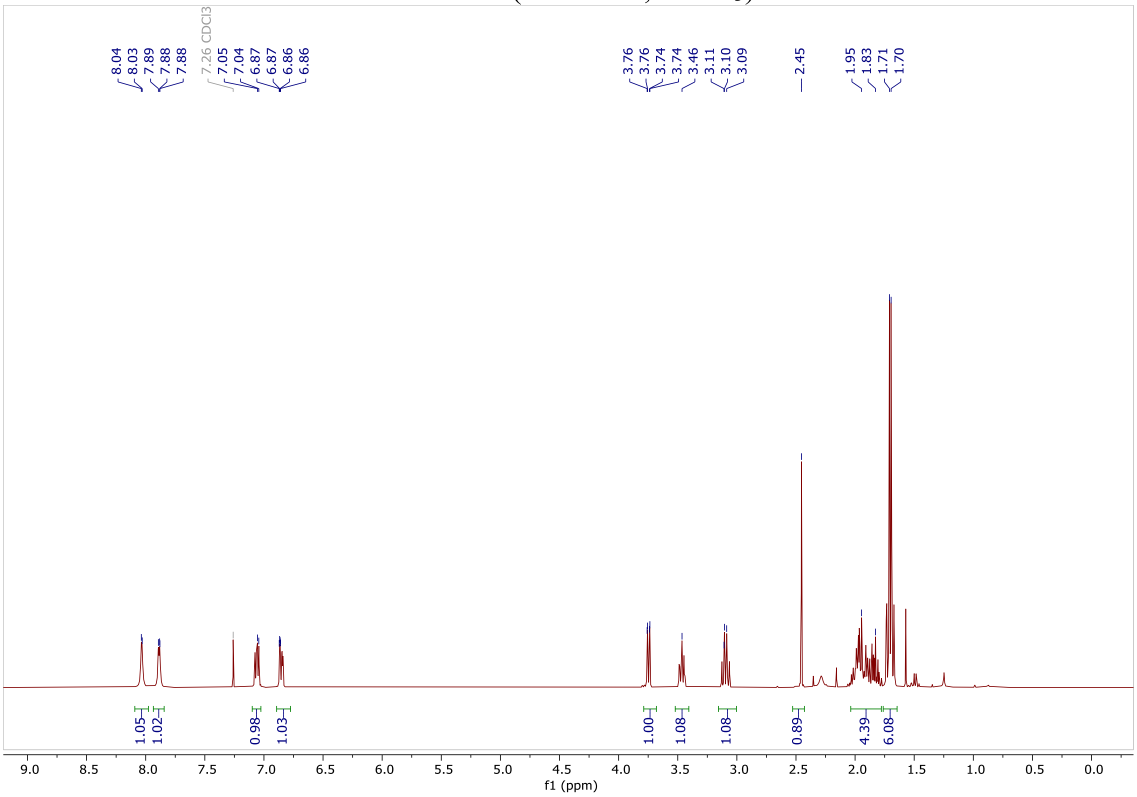

^{13}C NMR (101 MHz, $CDCl_3$)

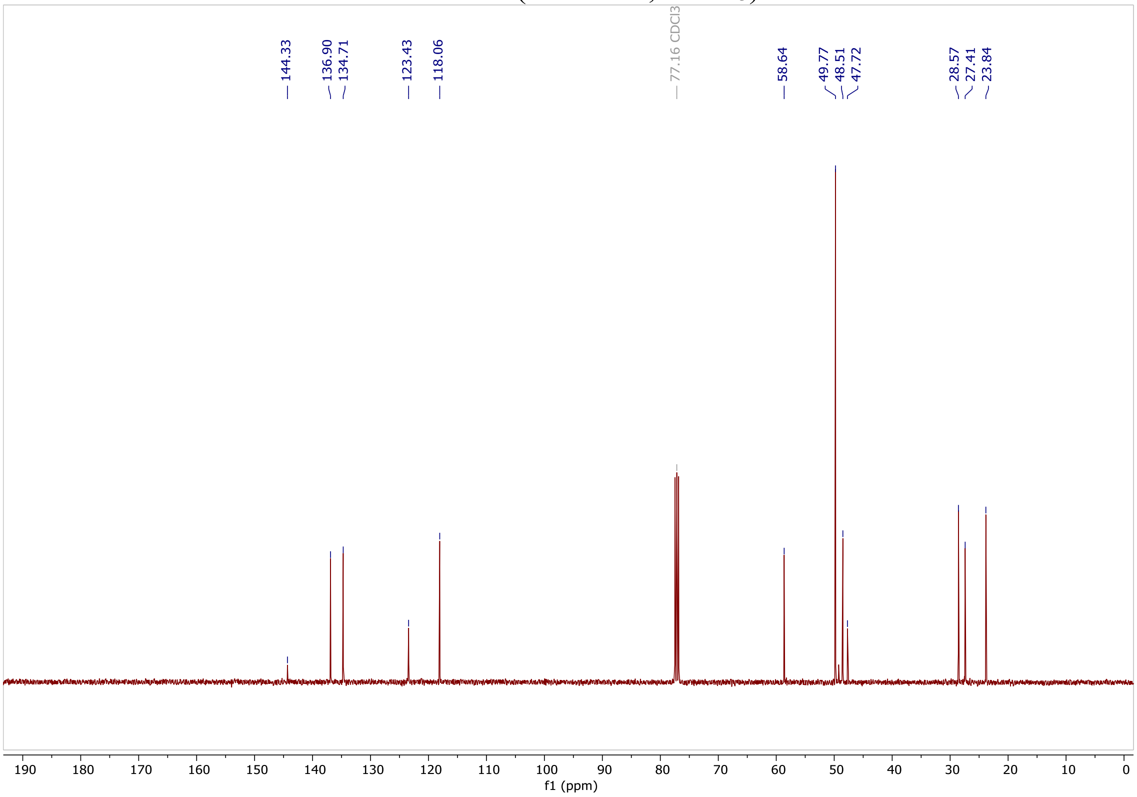

4-(2-(Bicyclo[1.1.1]pentan-1-yl)pyrrolidin-1-yl)pyridine, 3l

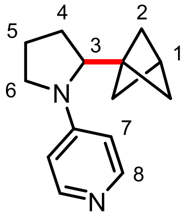

^{1}H NMR (400 MHz, $CDCl_3$)

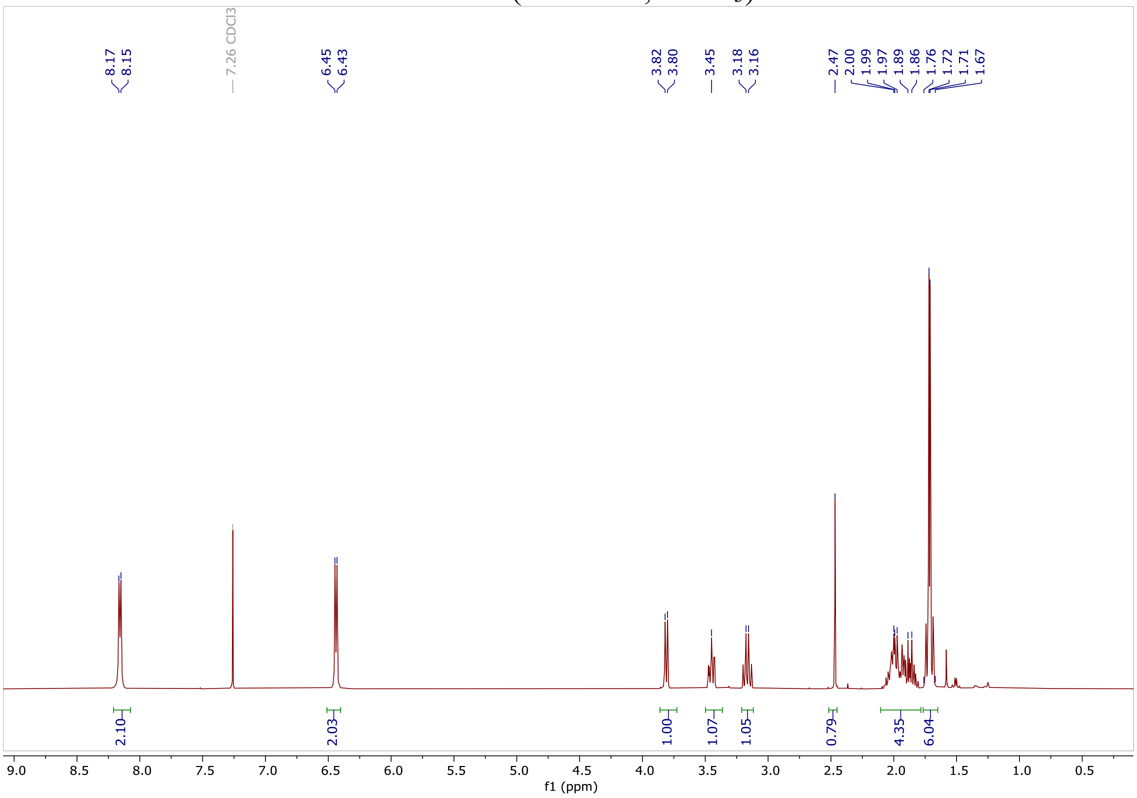

^{13}C NMR (101 MHz, $CDCl_3$)

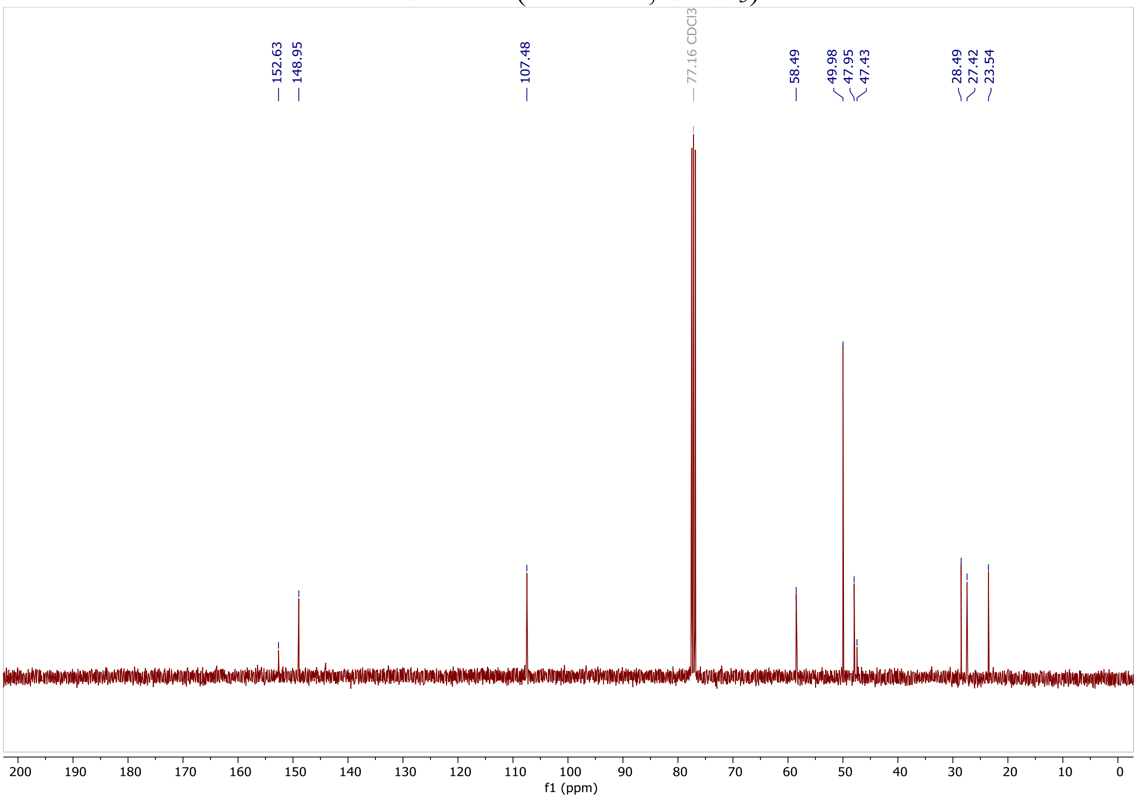

6-(2-(Bicyclo[1.1.1]pentan-1-yl)pyrrolidin-1-yl)quinoline, 3m

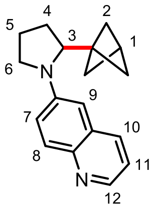

^{1}H NMR (400 MHz, $CDCl_3$)

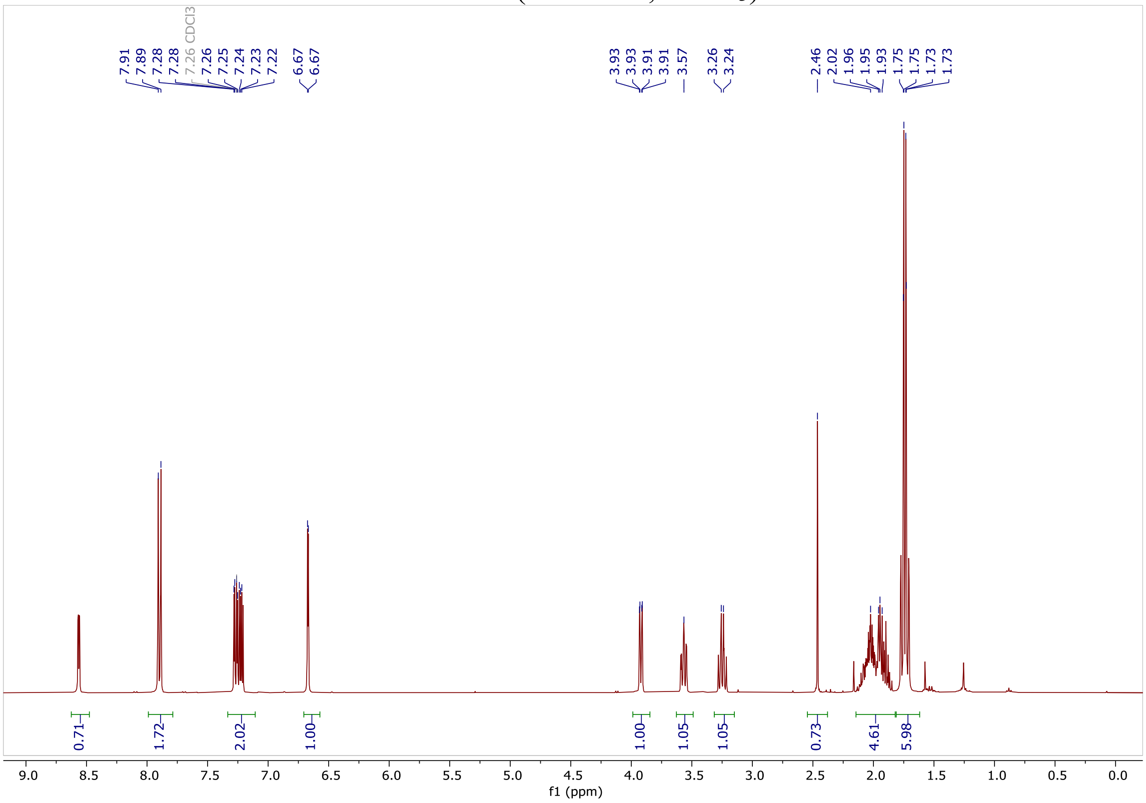

^{13}C NMR (101 MHz, $CDCl_3$)

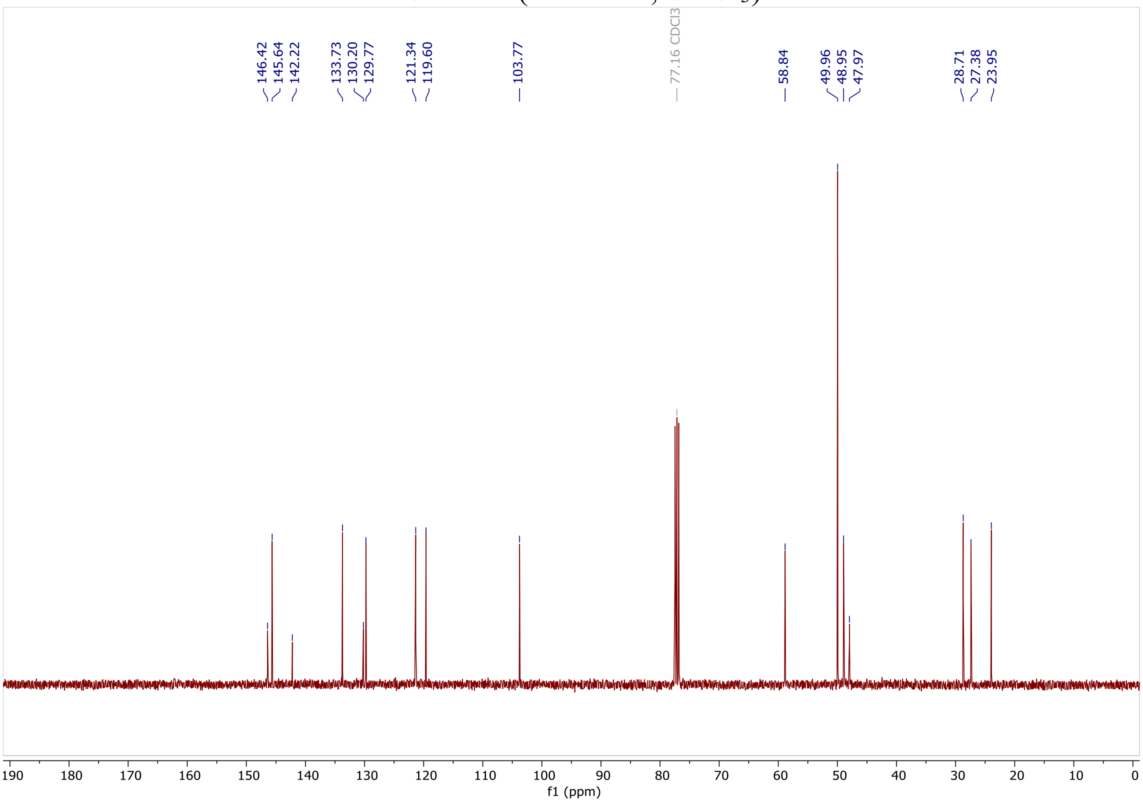

2-(Bicyclo[1.1.1]pentan-1-yl)-1-phenylpiperidine, 3n and 4n

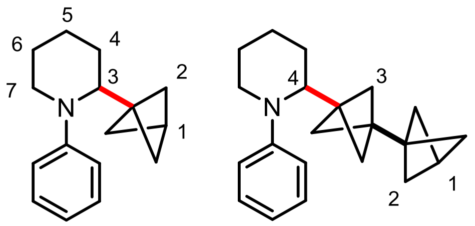

^{1}H NMR (400 MHz, $CDCl_3$)

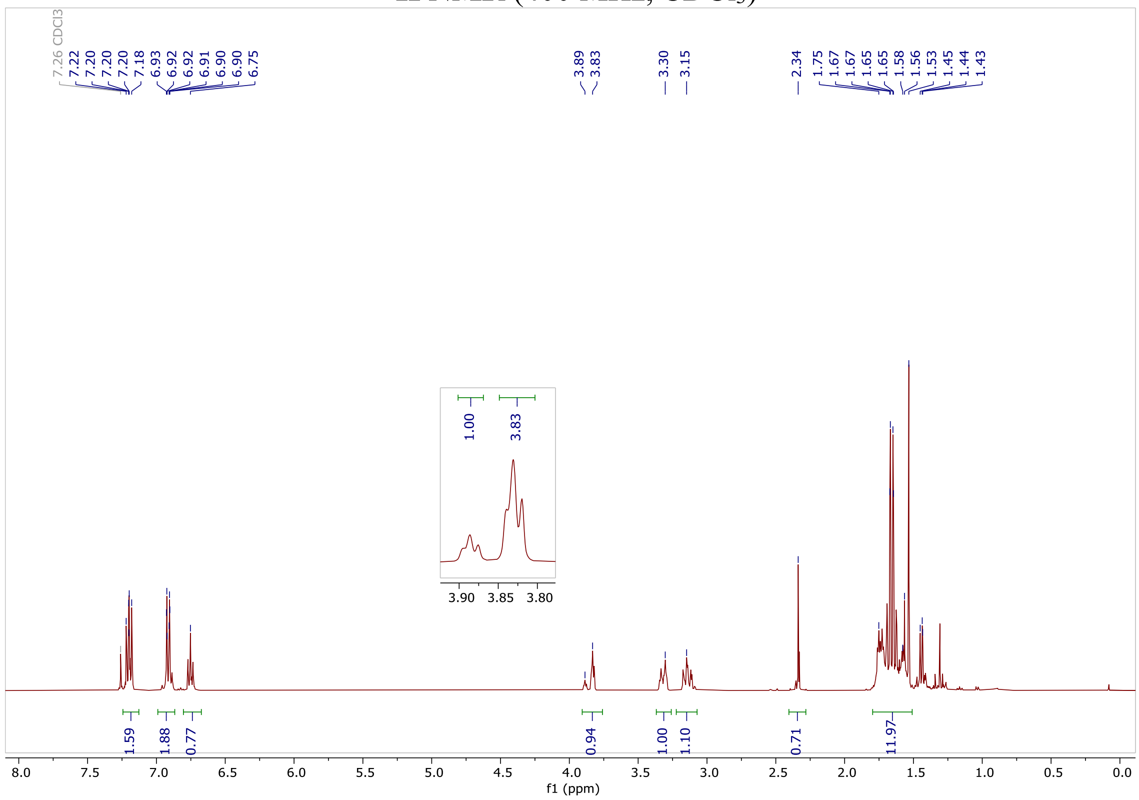

^{13}C NMR (101 MHz, $CDCl_3$)

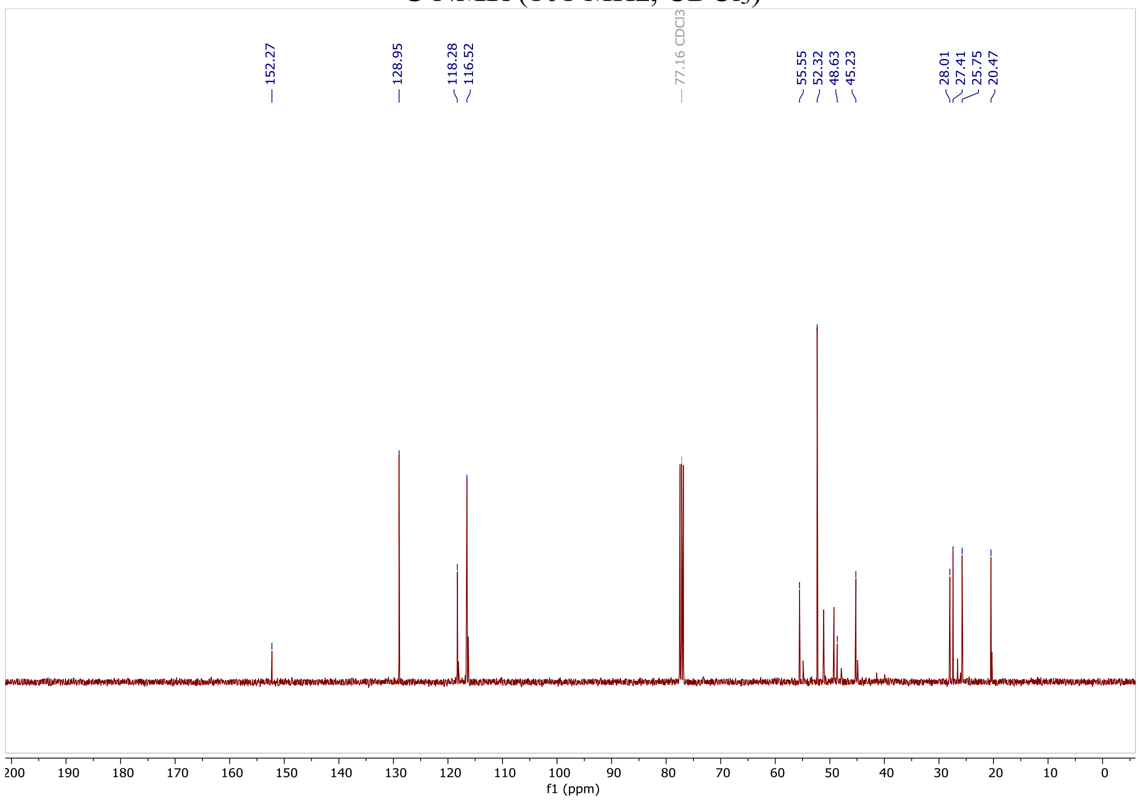

2-(Bicyclo[1.1.1]pentan-1-yl)-1-phenylazepane, 3o and 4o

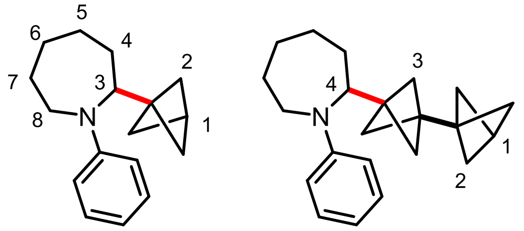

^{1}H NMR (400 MHz, $CDCl_3$)

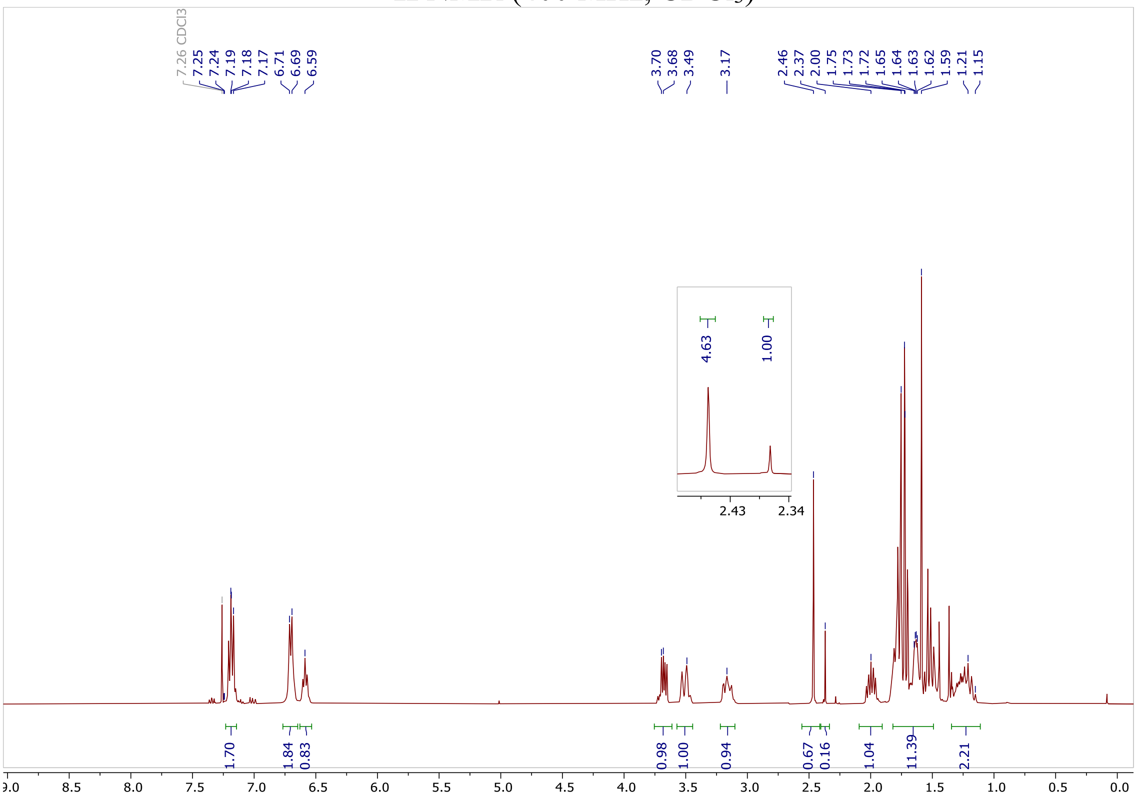

^{13}C NMR (101 MHz, $CDCl_3$)

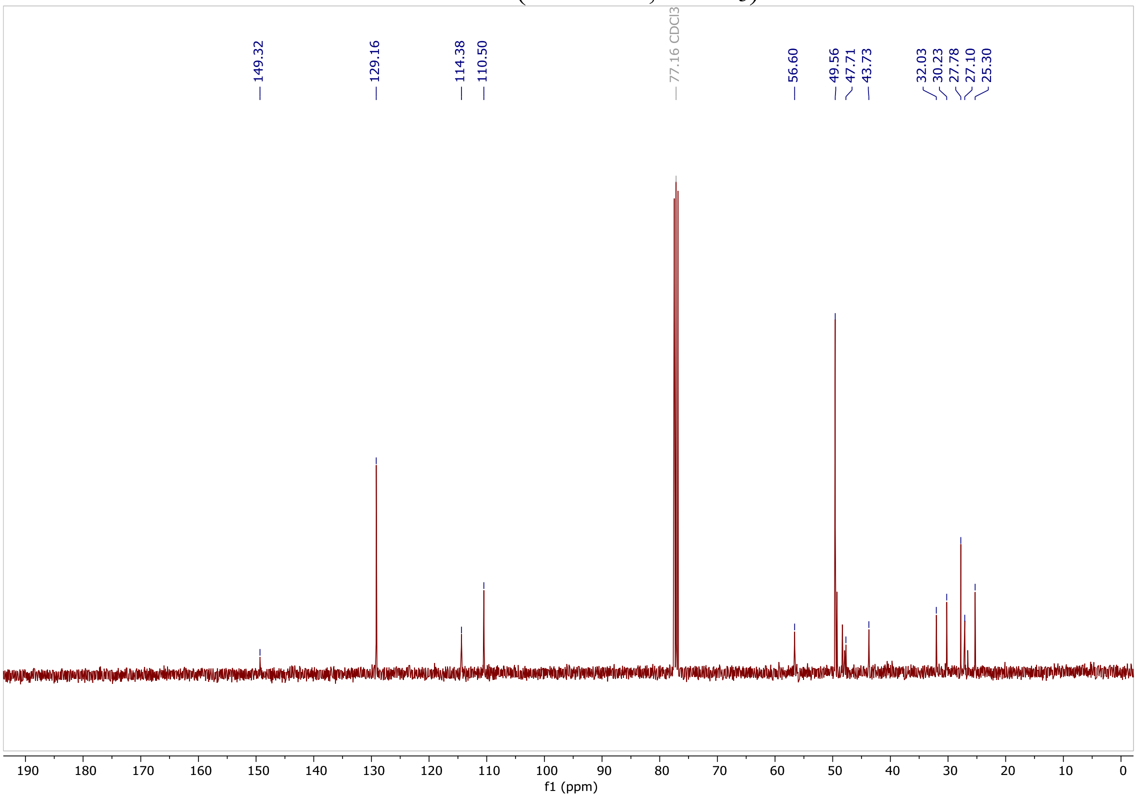

2-(Bicyclo[1.1.1]pentan-1-yl)-1-(pyridin-3-yl)azepane, 3p

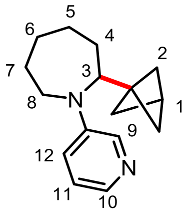

^{1}H NMR (400 MHz, $CDCl_3$)

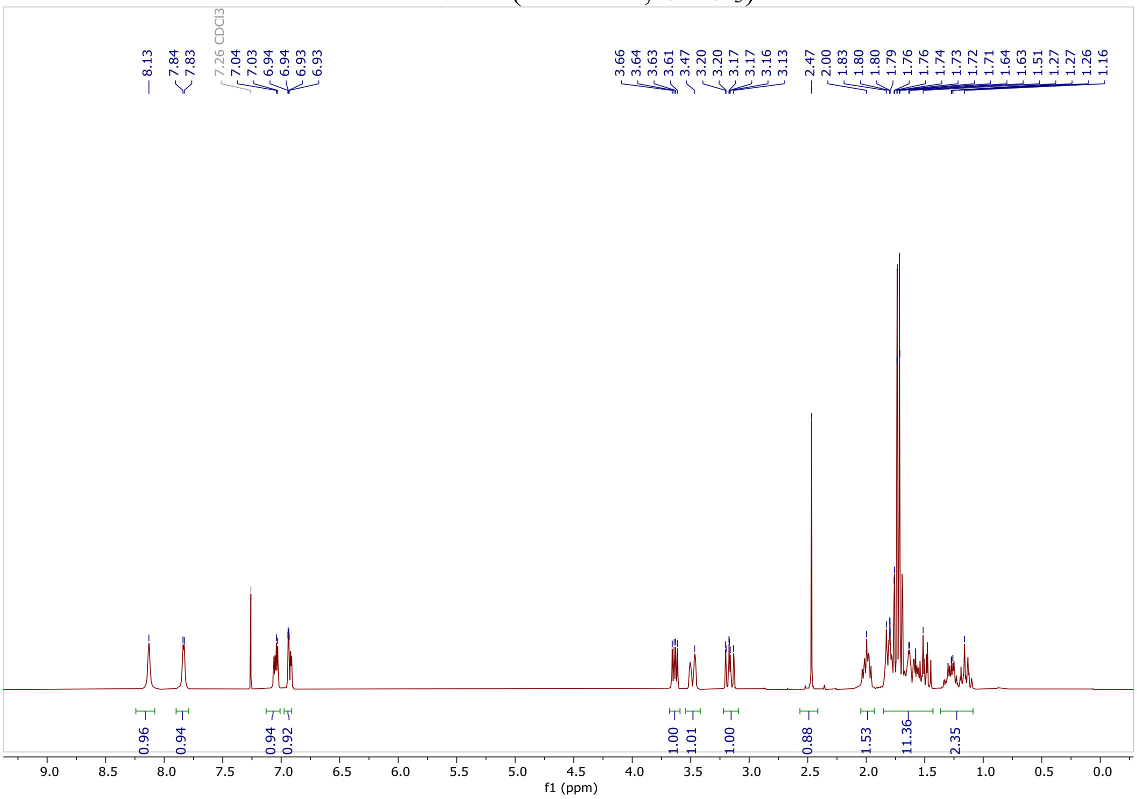

^{13}C NMR (101 MHz, $CDCl_3$)

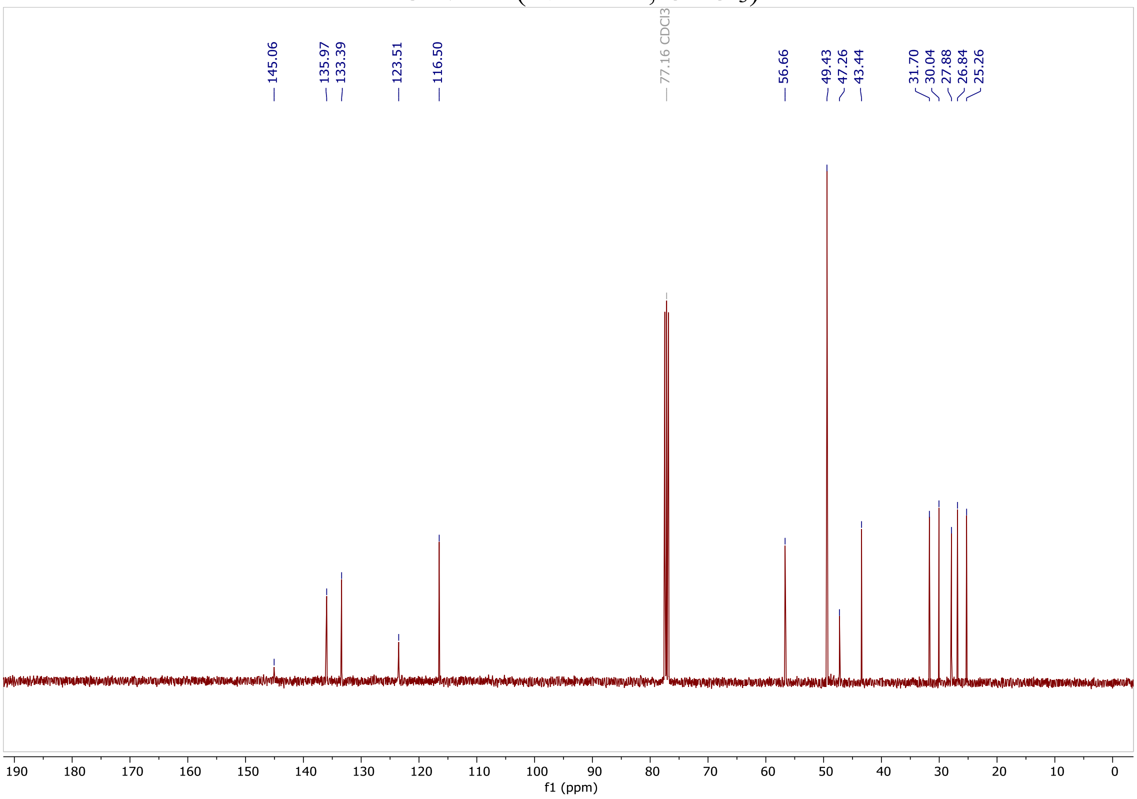

3-(Bicyclo[1.1.1]pentan-1-yl)-4-phenylmorpholine, 3q and 4q

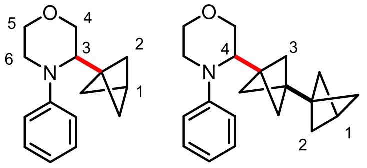

^{1}H NMR (400 MHz, $CDCl_3$)

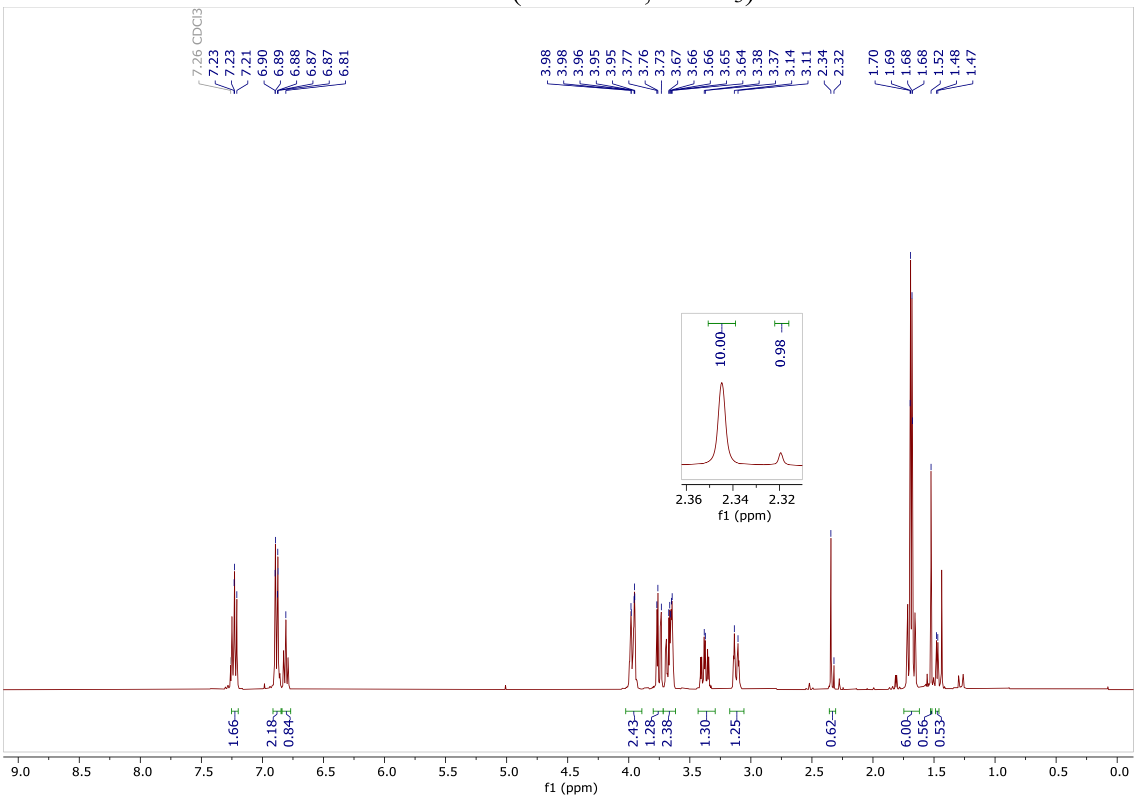

^{13}C NMR (101 MHz, $CDCl_3$)

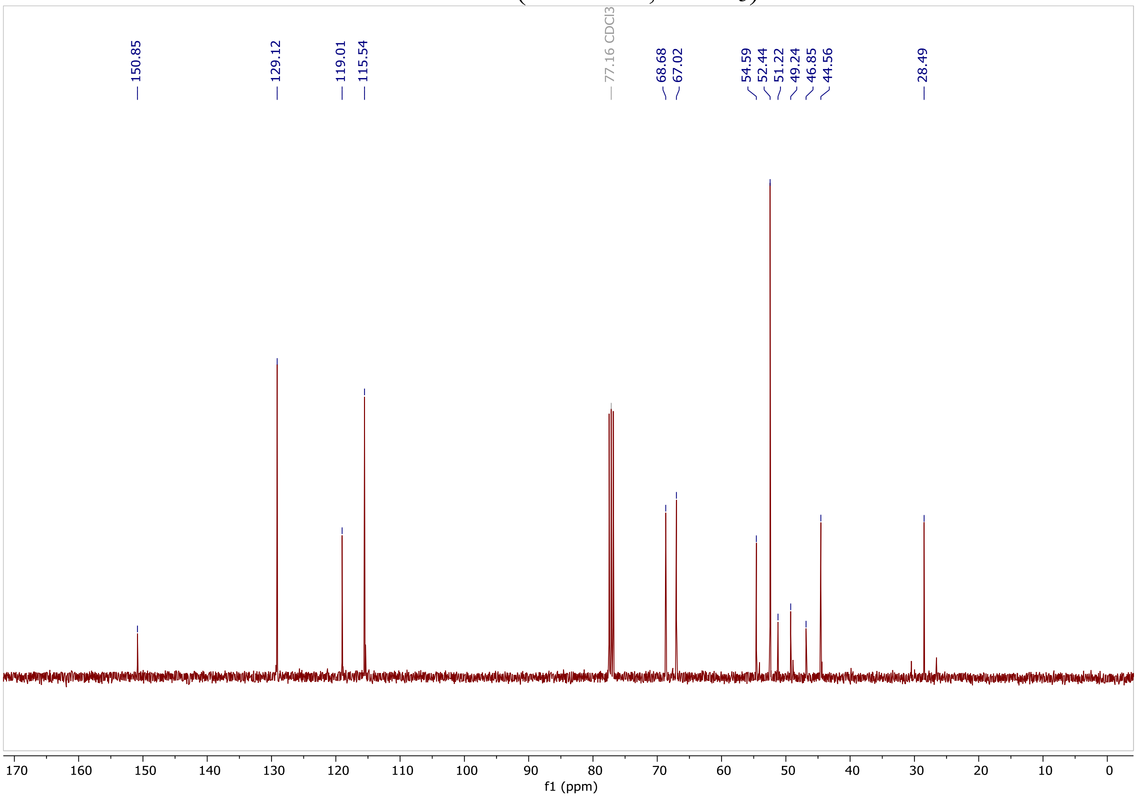

1-(Bicyclo[1.1.1]pentan-1-yl)-2-(pyridin-3-yl)octahydrocyclopenta[c]pyrrole, 3r

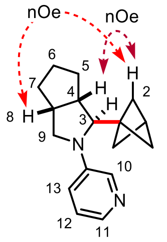

^{1}H NMR (400 MHz, CDCl$_3$)

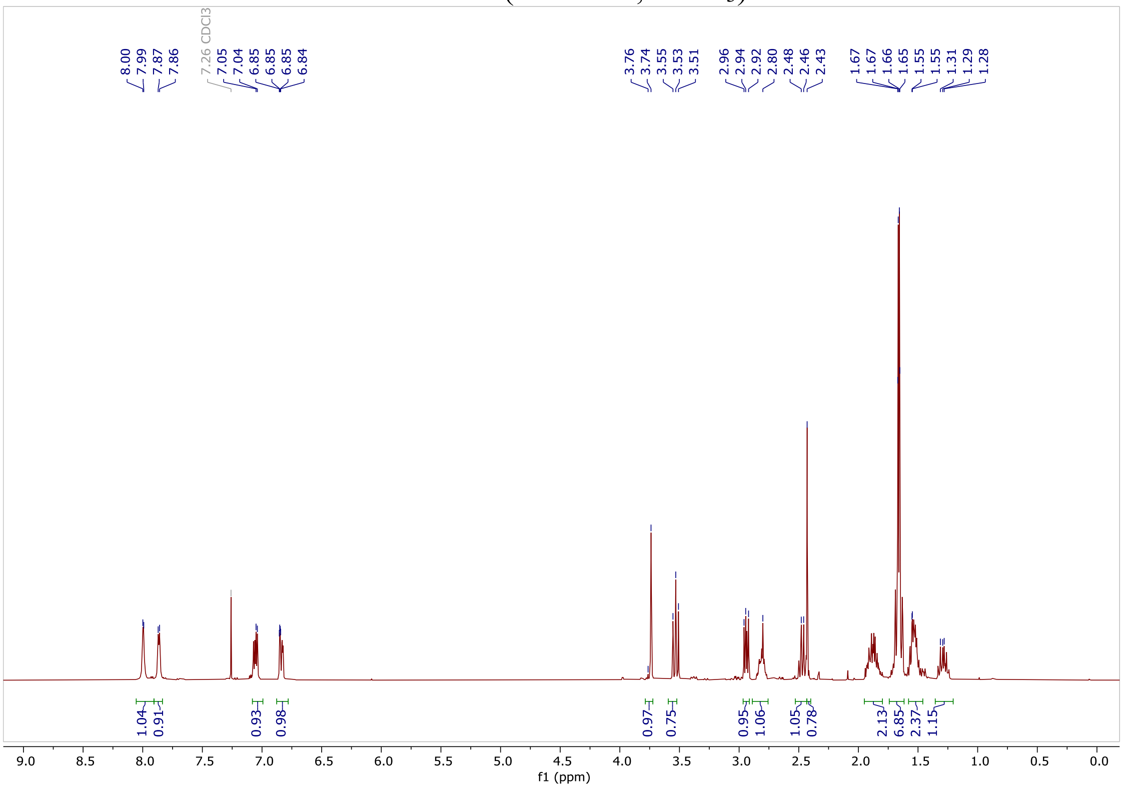

^{13}C NMR (101 MHz, CDCl$_3$)

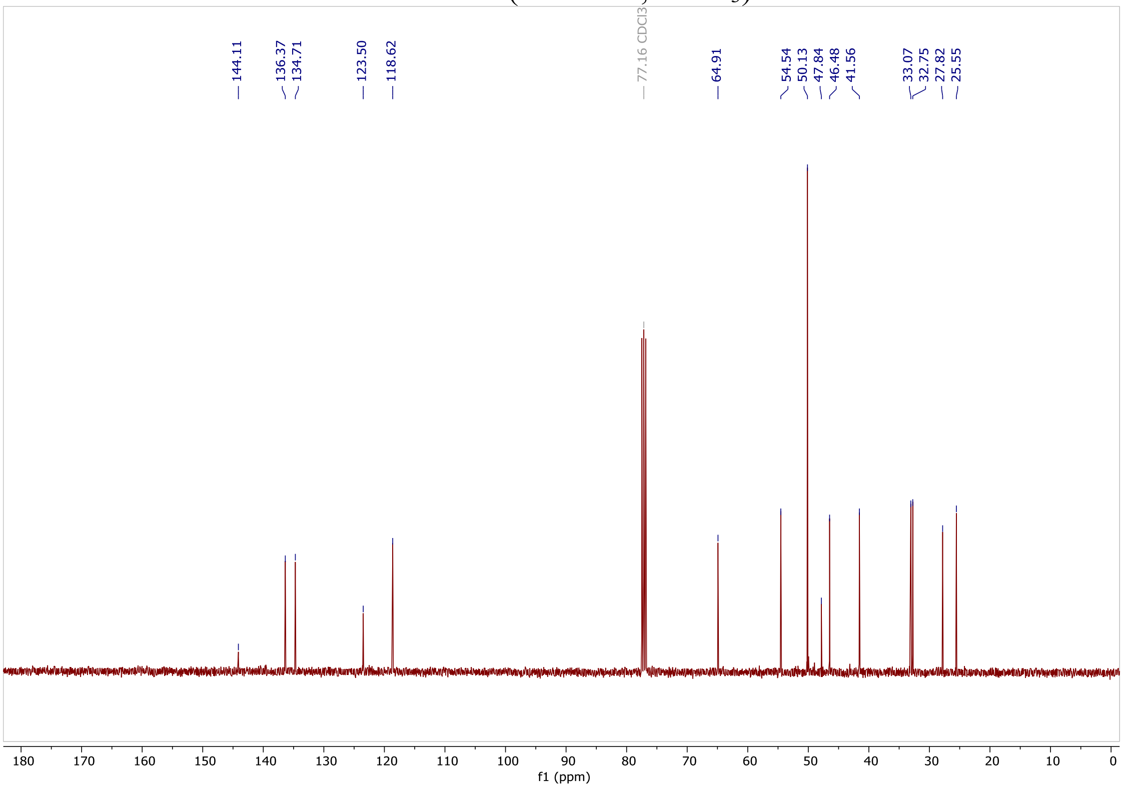

NOESY (400 MHz, $CDCl_3$)

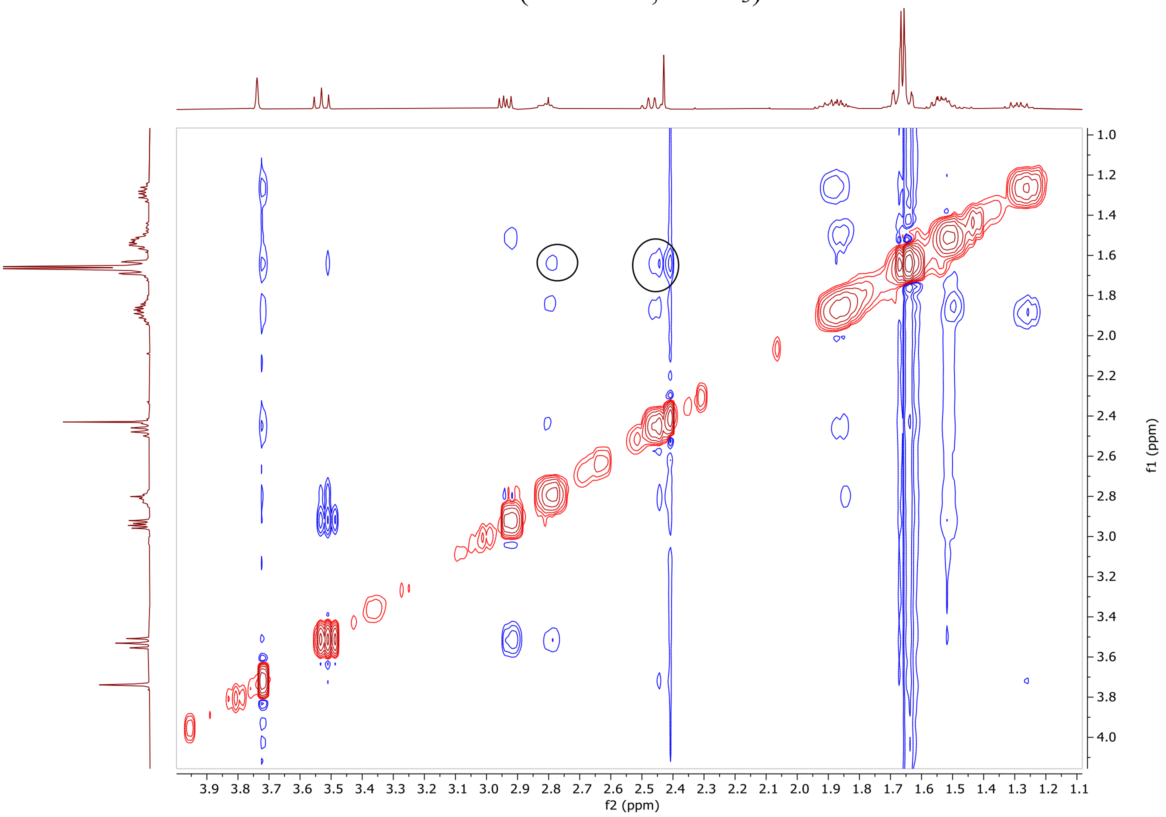

tert-Butyl 2-(bicyclo[1.1.1]pentan-1-yl)-1-(pyridin-3-yl)pyrrolidin-3-yl)carbamate, 3s

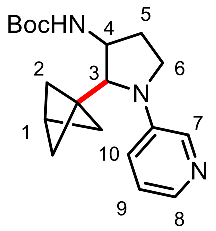

^{1}H NMR (400 MHz, $CDCl_3$)

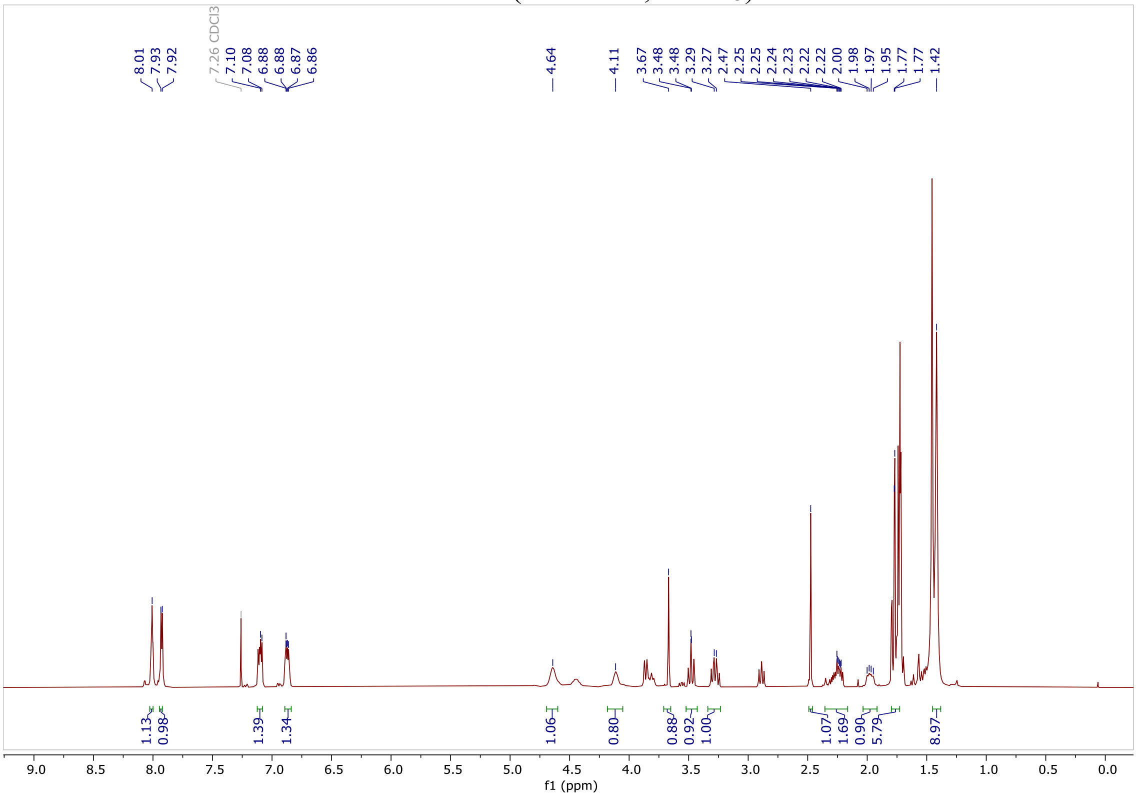

^{13}C NMR (101 MHz, $CDCl_3$)

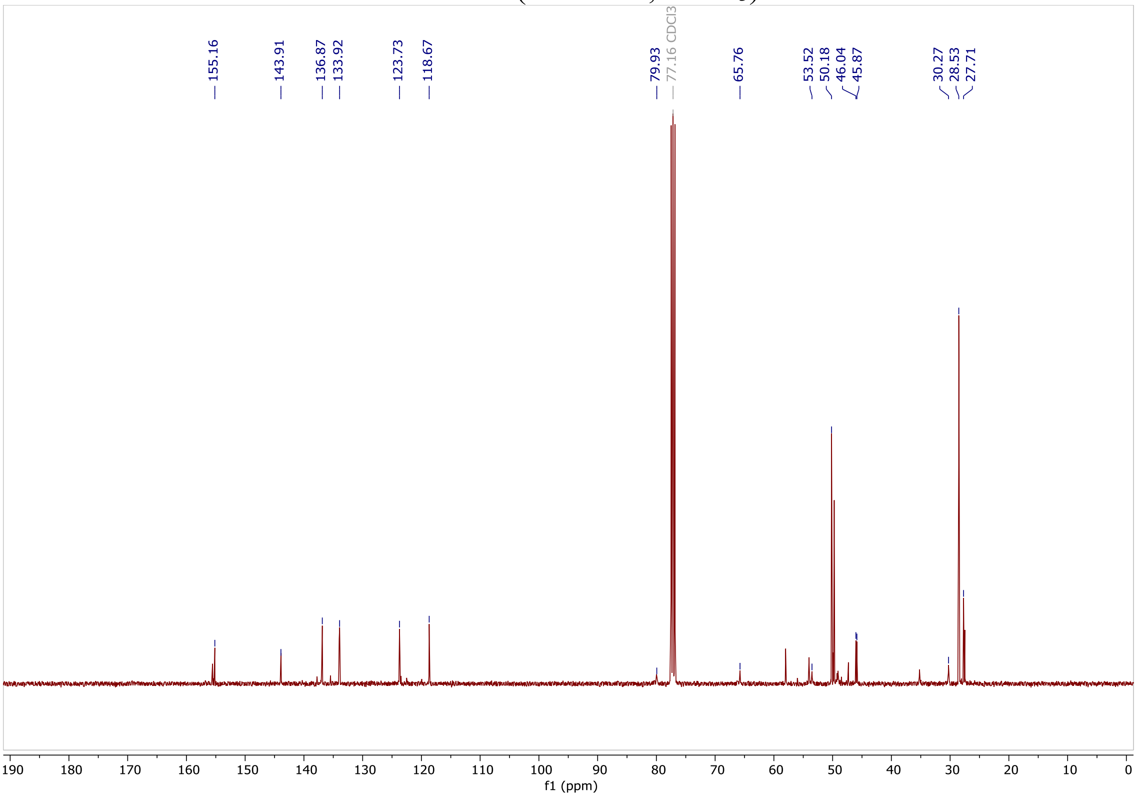

tert-butyl 5-(bicyclo[1.1.1]pentan-1-yl)-1-(pyridin-3-yl)pyrrolidin-3-yl)carbamate, 3t

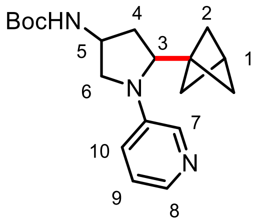

^{1}H NMR (400 MHz, CDCl$_3$)

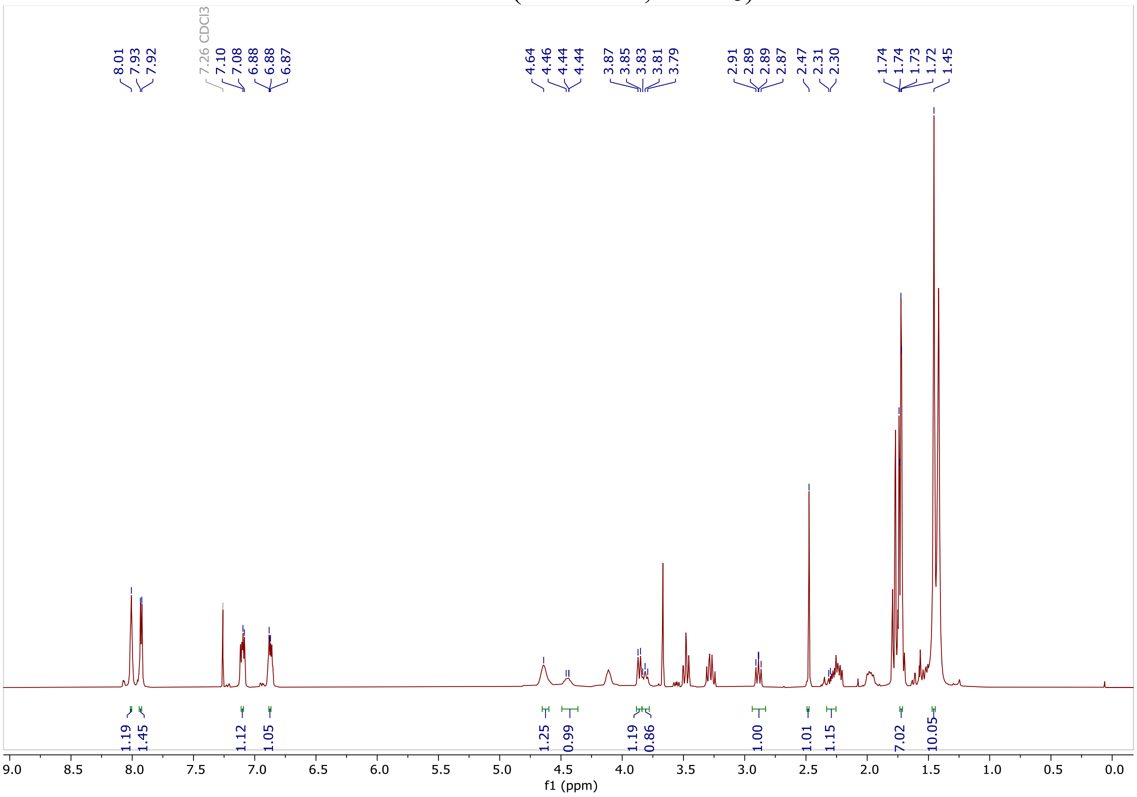

^{13}C NMR (101 MHz, CDCl$_3$)

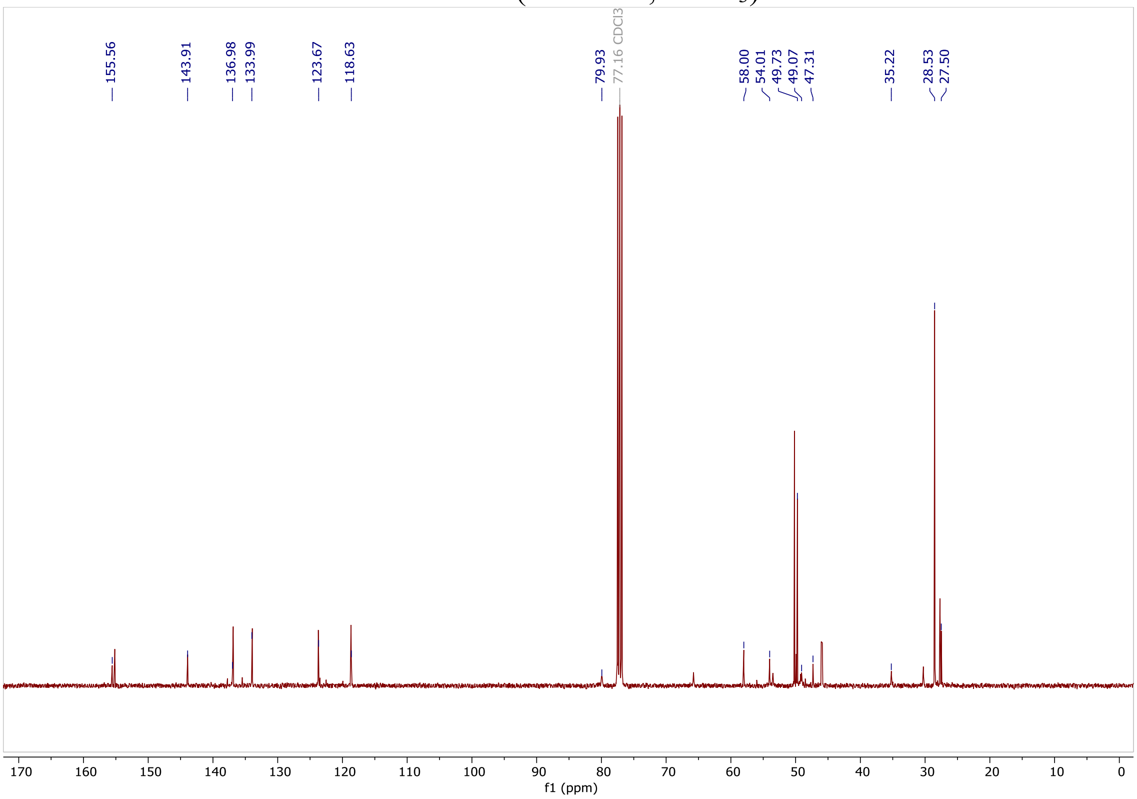

2-(Bicyclo[3.1.1]heptan-1-yl)-1-phenylpyrrolidine, 8a and 9a

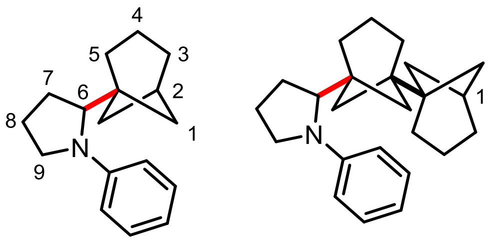

^{1}H NMR (400 MHz, $CDCl_3$)

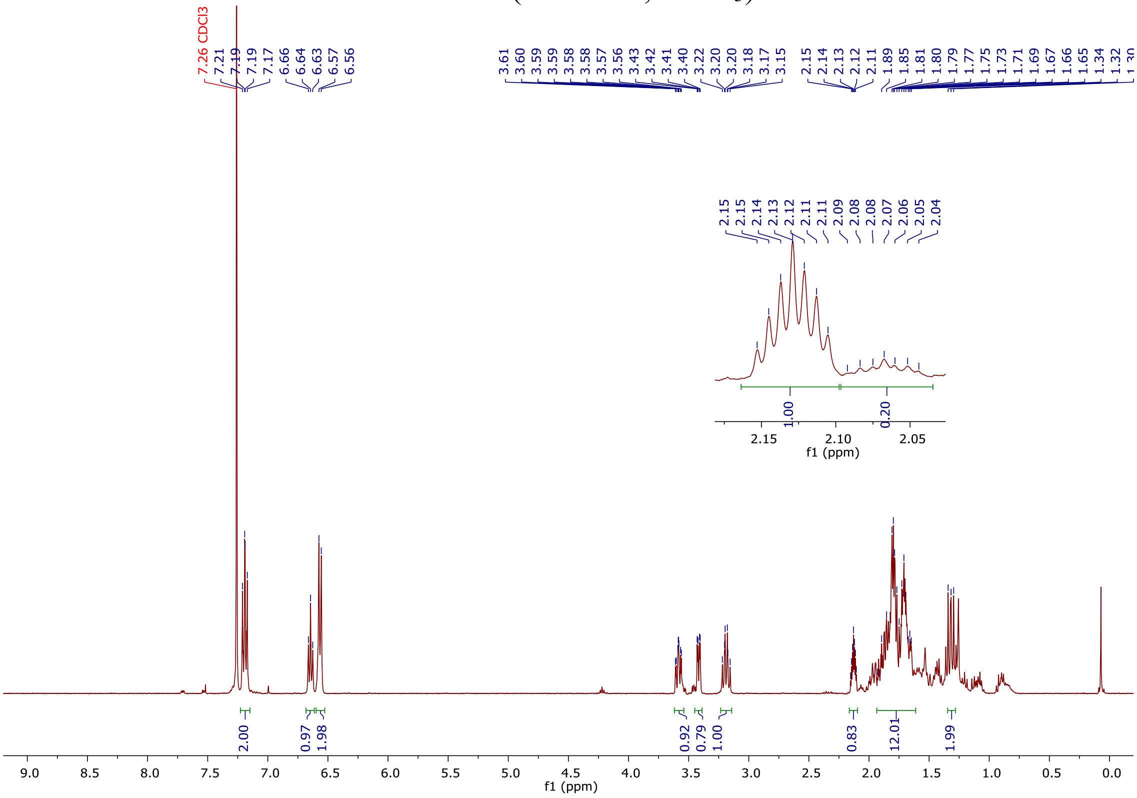

^{13}C NMR (126 MHz, $CDCl_3$)

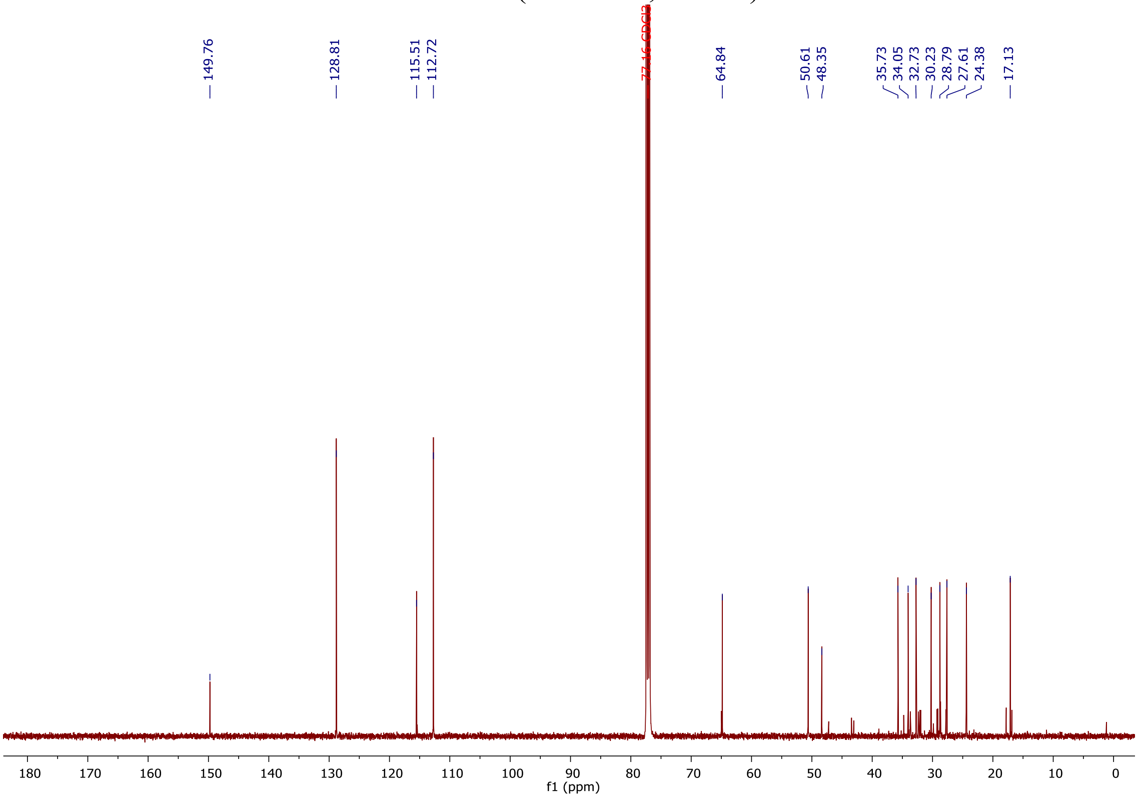

2-(Bicyclo[3.1.1]heptan-1-yl)-1-(4-(methylsulfonyl)phenyl)pyrrolidine, 8b

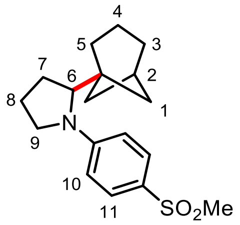

^{1}H NMR (400 MHz, $CDCl_3$)

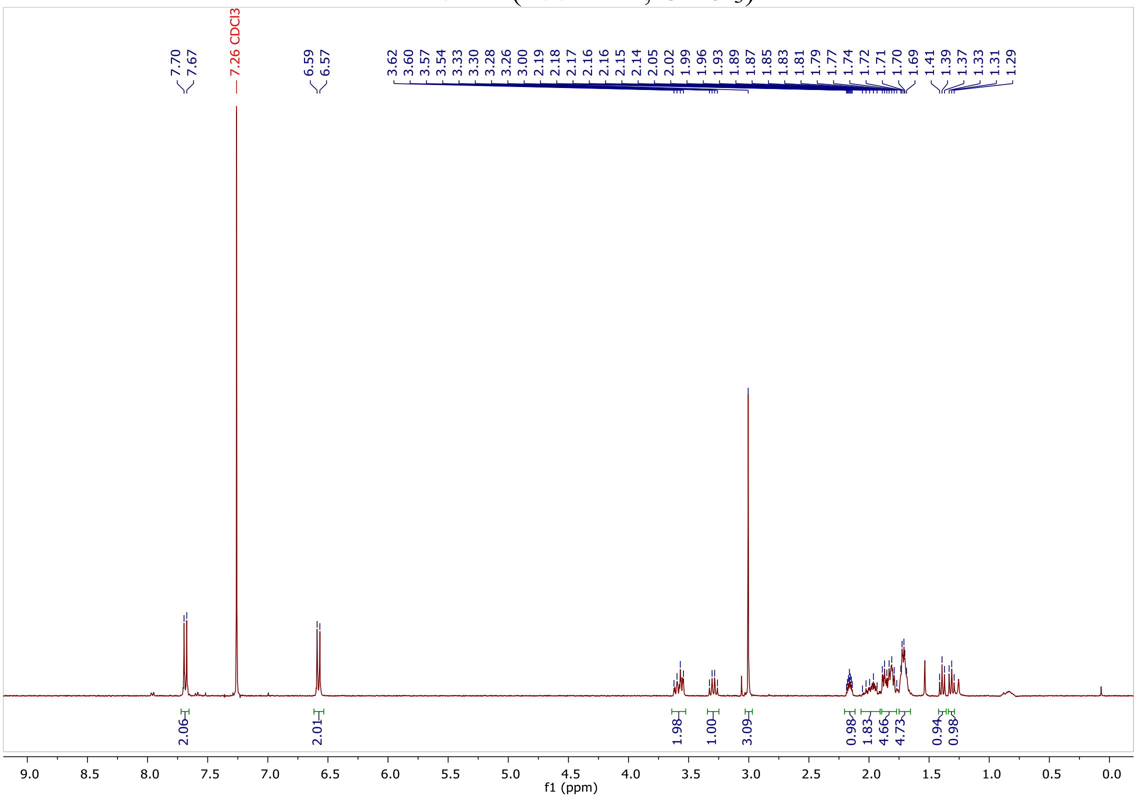

^{13}C NMR (101 MHz, $CDCl_3$)

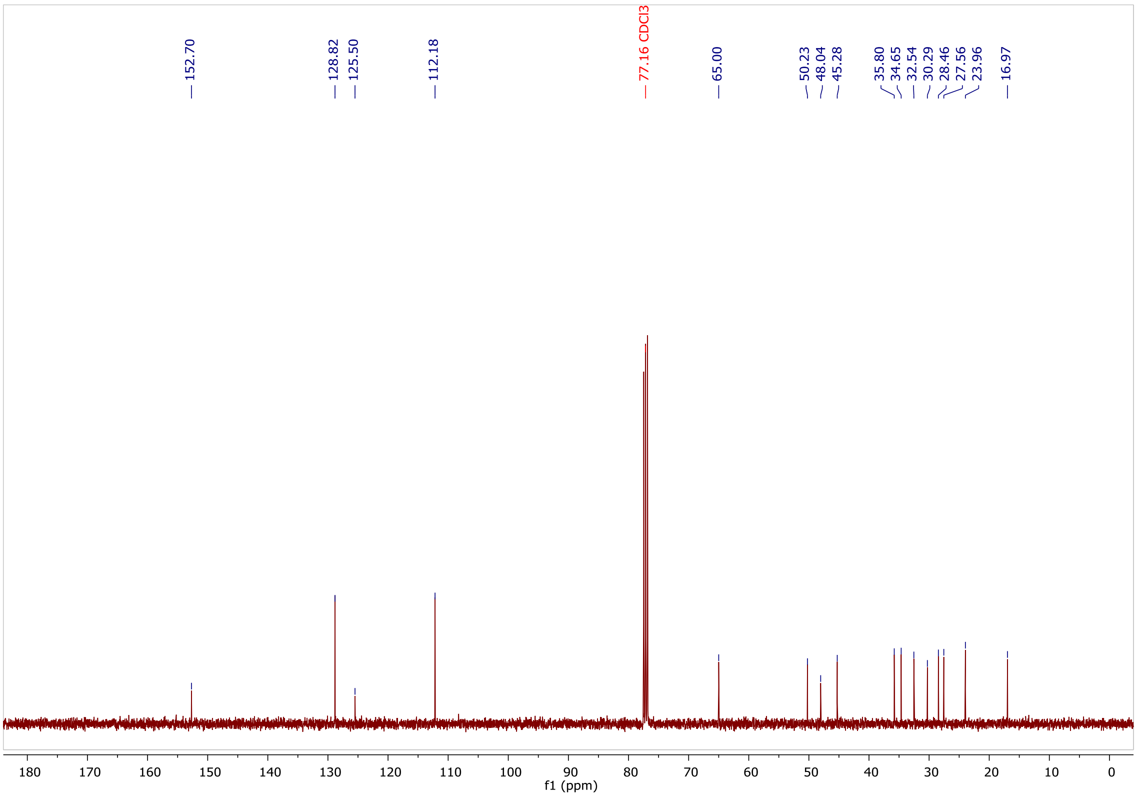

Methyl 4-(2-(bicyclo[3.1.1]heptan-1-yl)pyrrolidin-1-yl)benzoate, 8c

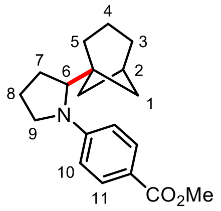

^{1}H NMR (600 MHz, $CDCl_3$)

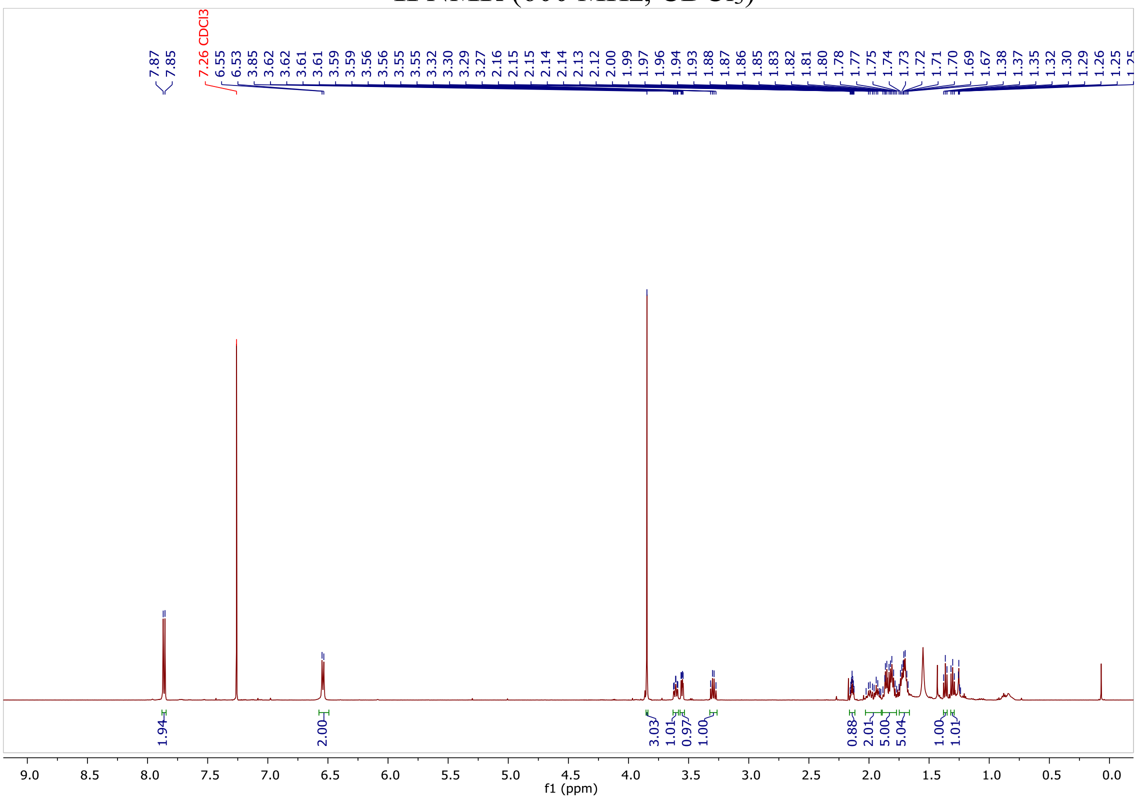

^{13}C NMR (151 MHz, $CDCl_3$)

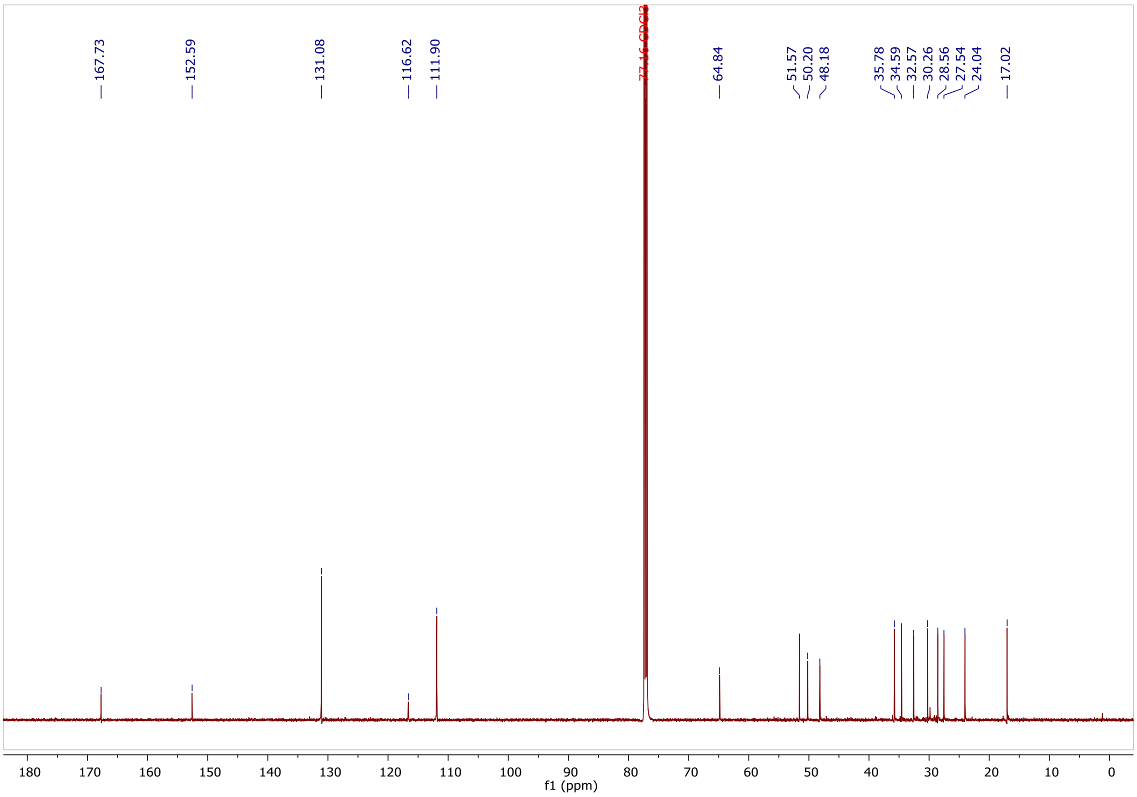

2-(Bicyclo[3.1.1]heptan-1-yl)-1-(4-fluorophenyl)pyrrolidine, 8d and 9d

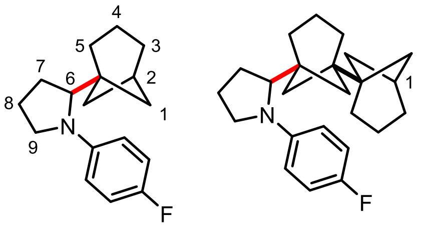

^{1}H NMR (400 MHz, $CDCl_3$)

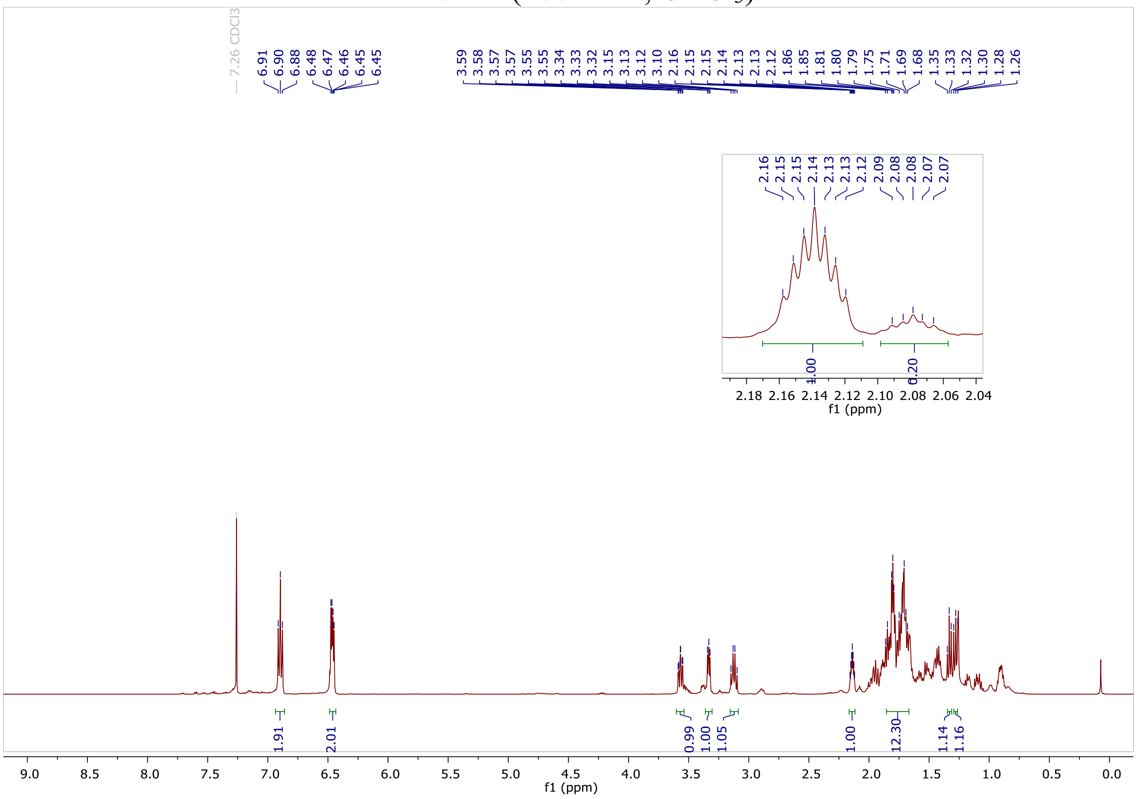

^{13}C NMR (126 MHz, $CDCl_3$)

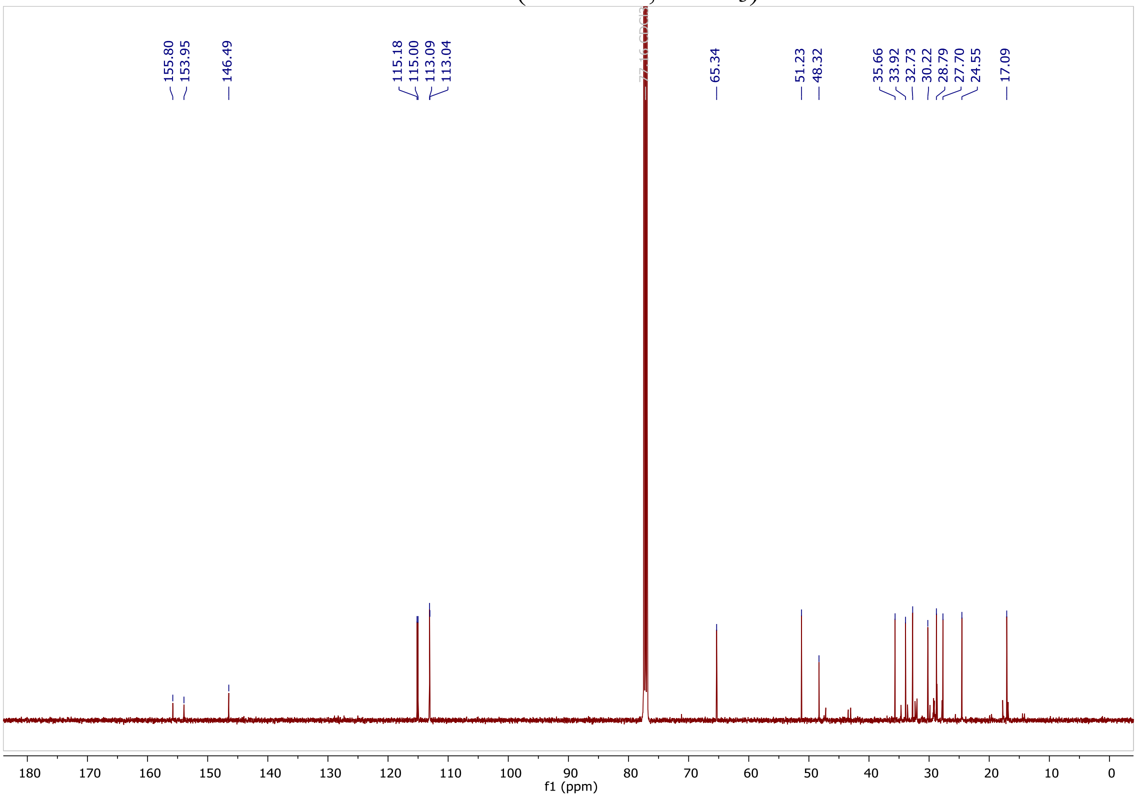

^{19}F NMR (377 MHz, $CDCl_3$)

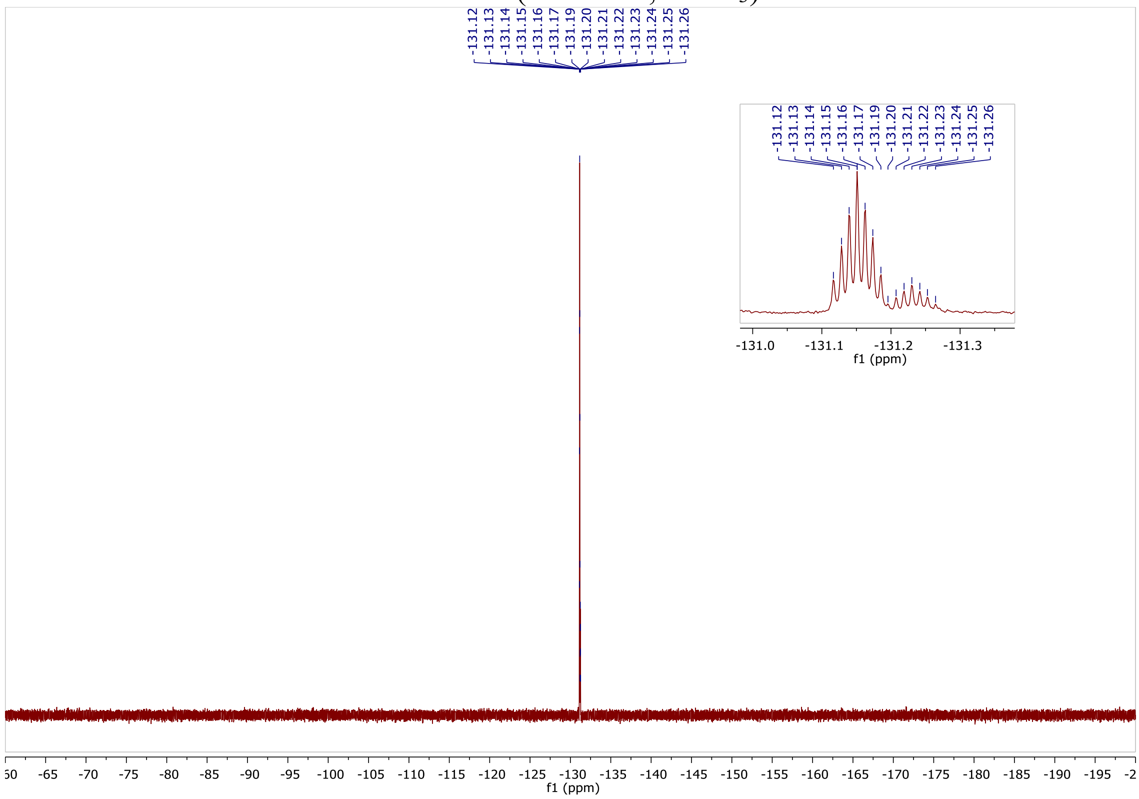

3-(2-(Bicyclo[3.1.1]heptan-1-yl)pyrrolidin-1-yl)benzonitrile, 8e

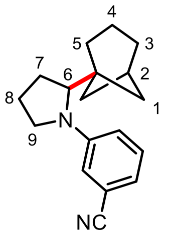

^{1}H NMR (600 MHz, $CDCl_3$)

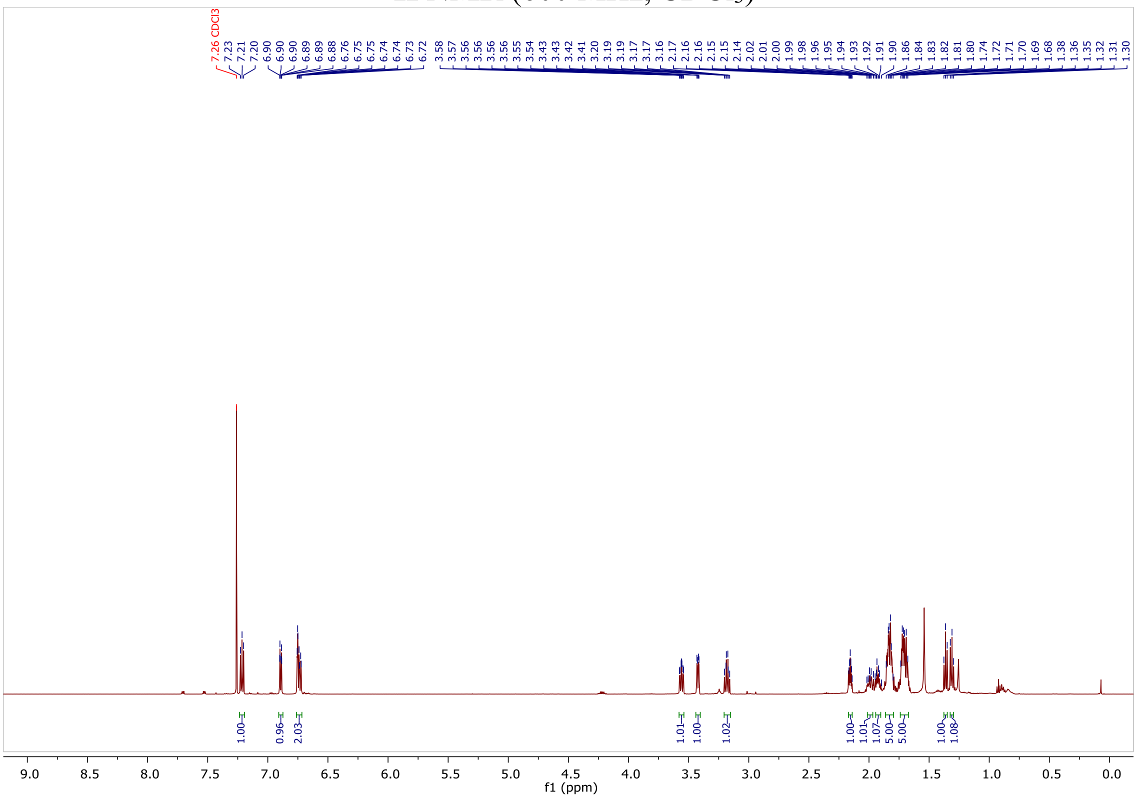

^{13}C NMR (126 MHz, $CDCl_3$)

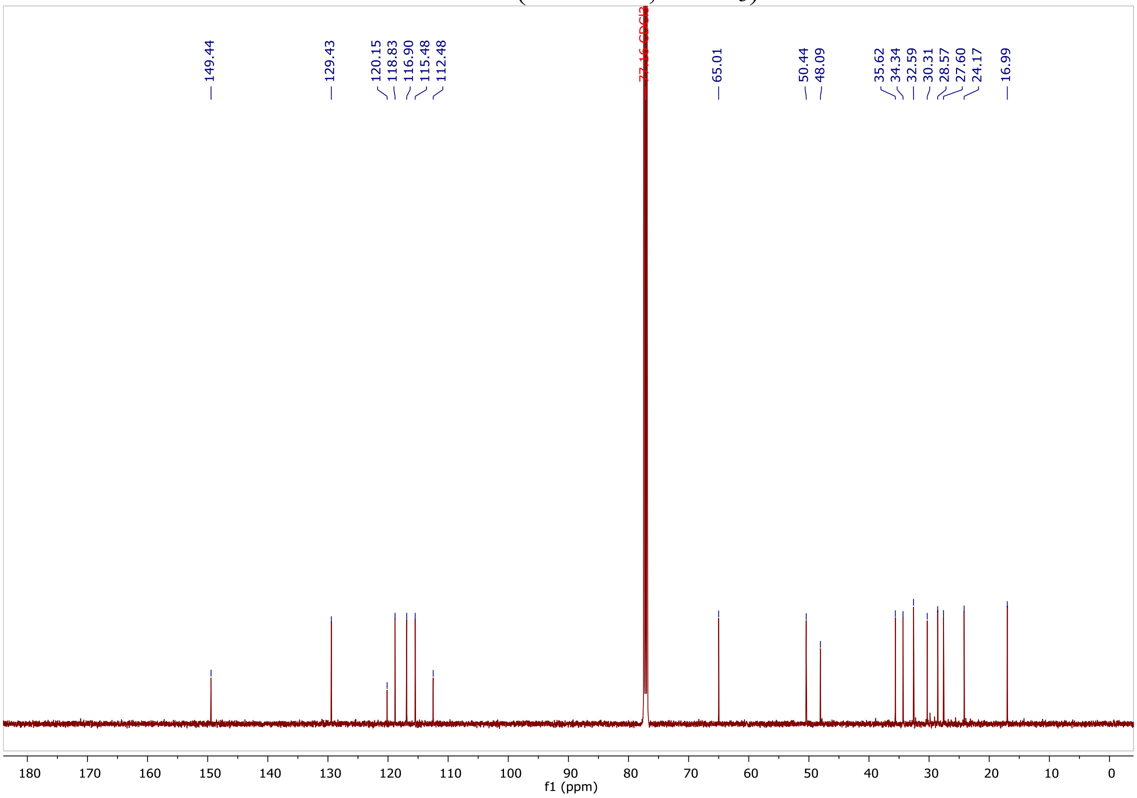

2-(2-(Bicyclo[3.1.1]heptan-1-yl)pyrrolidin-1-yl)pyridine, 8f and 9f

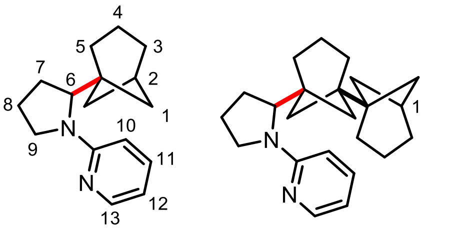

^{1}H NMR (600 MHz, CDCl$_3$)

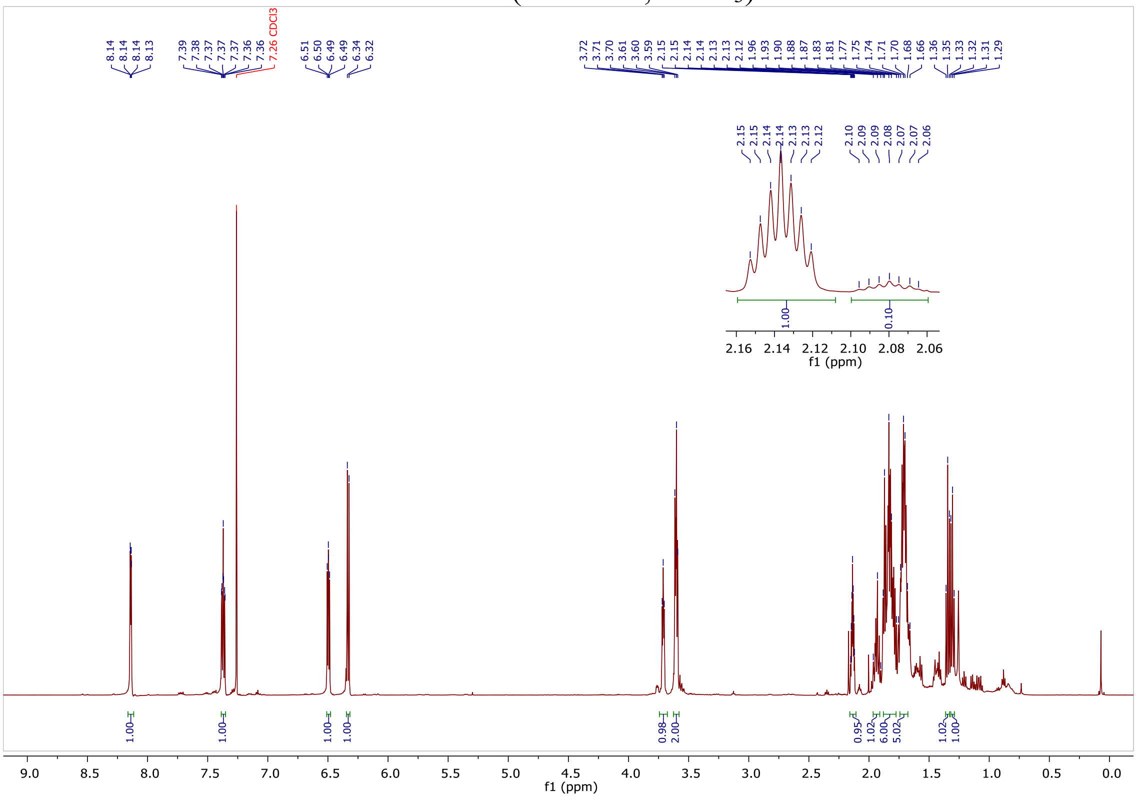

^{13}C NMR (151 MHz, CDCl$_3$)

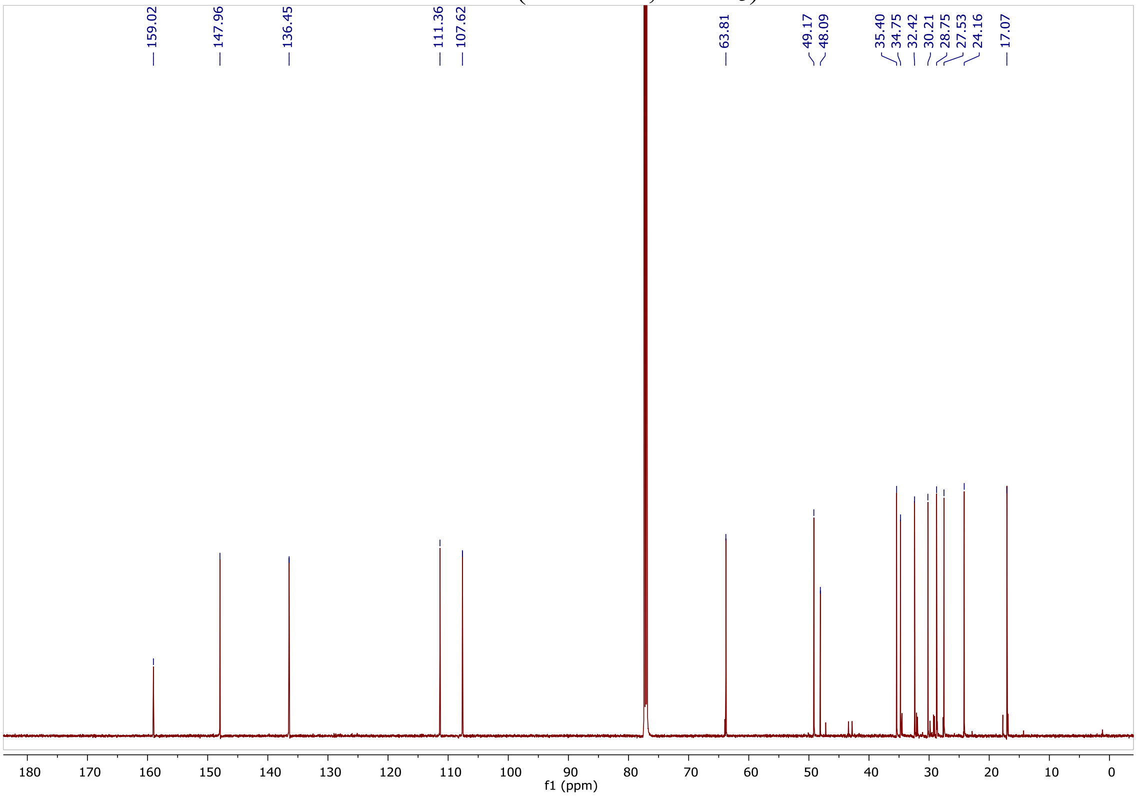

3-(Bicyclo[3.1.1]heptan-1-yl)-4-phenylmorpholine, 8g

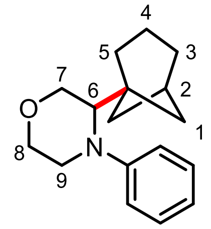

^{1}H NMR (400 MHz, $CDCl_3$)

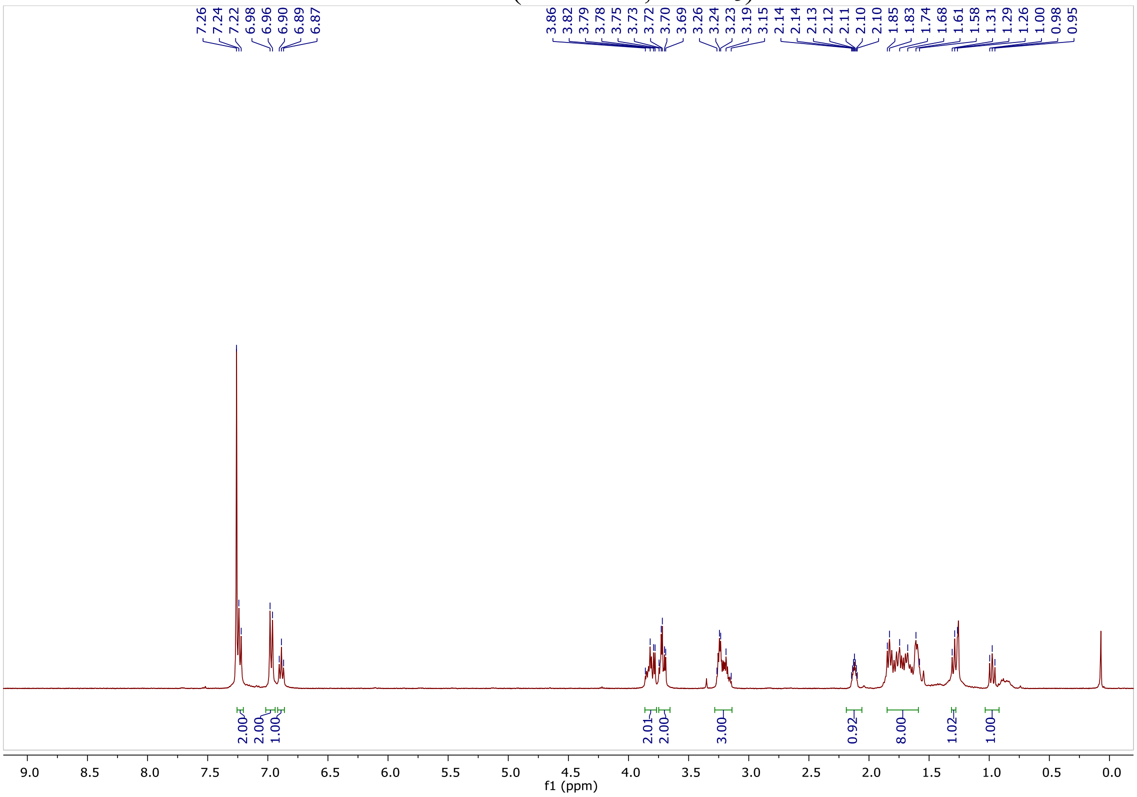

^{13}C NMR (101 MHz, $CDCl_3$)

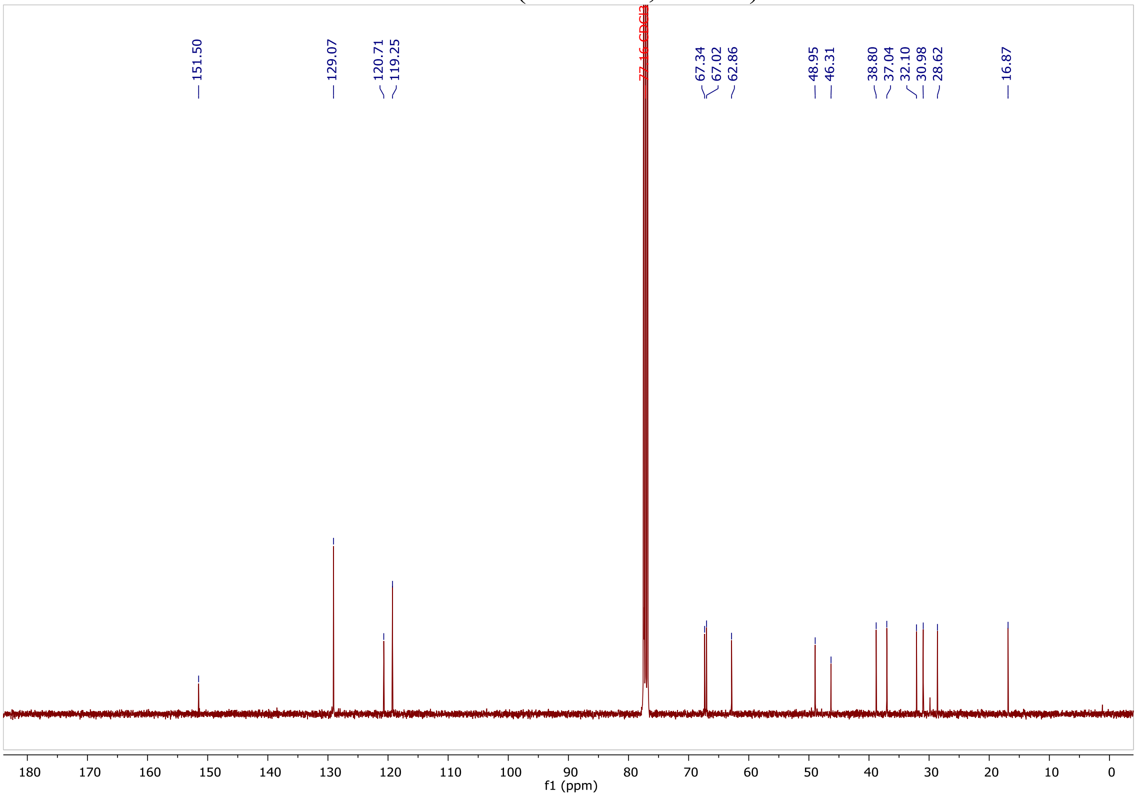

3-((2*S*,5*S*)-5-(Bicyclo[1.1.1]pentan-1-yl)-1-methylpyrrolidin-2-yl)pyridine, 7 and 10

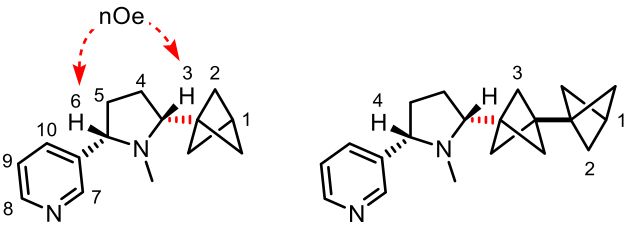

^{1}H NMR (400 MHz, $CDCl_3$)

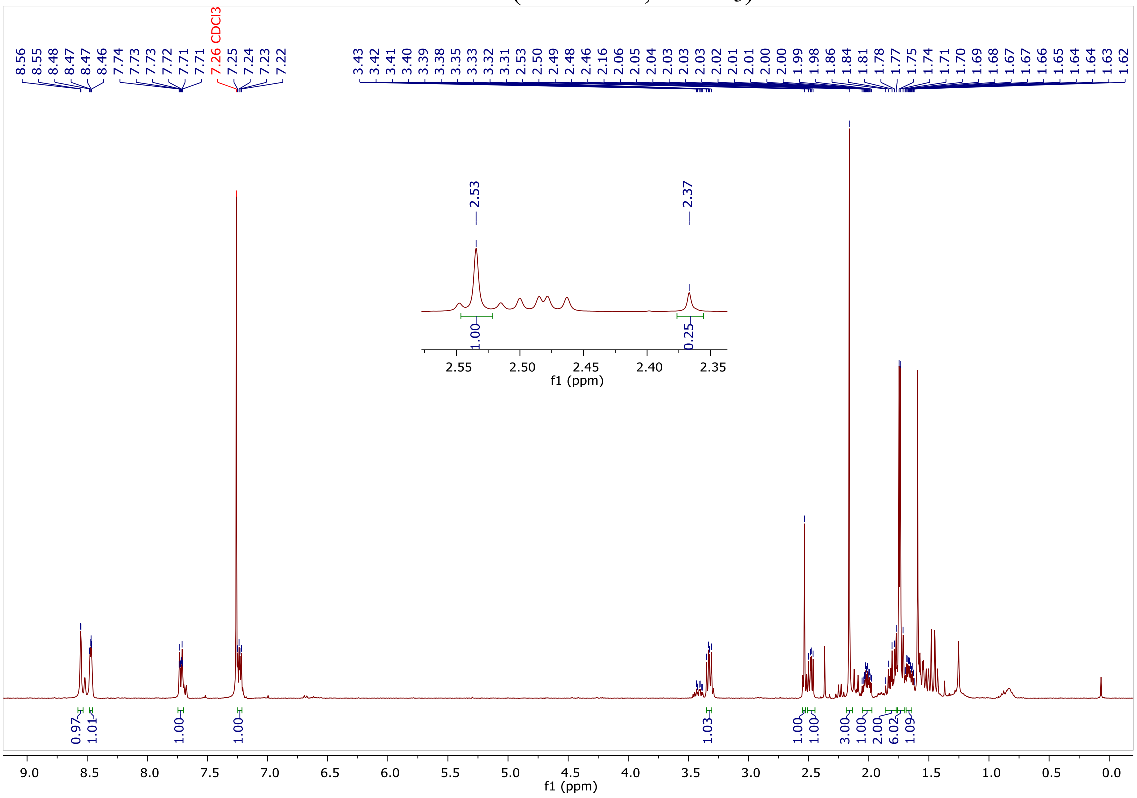

^{13}C NMR (151 MHz, $CDCl_3$)

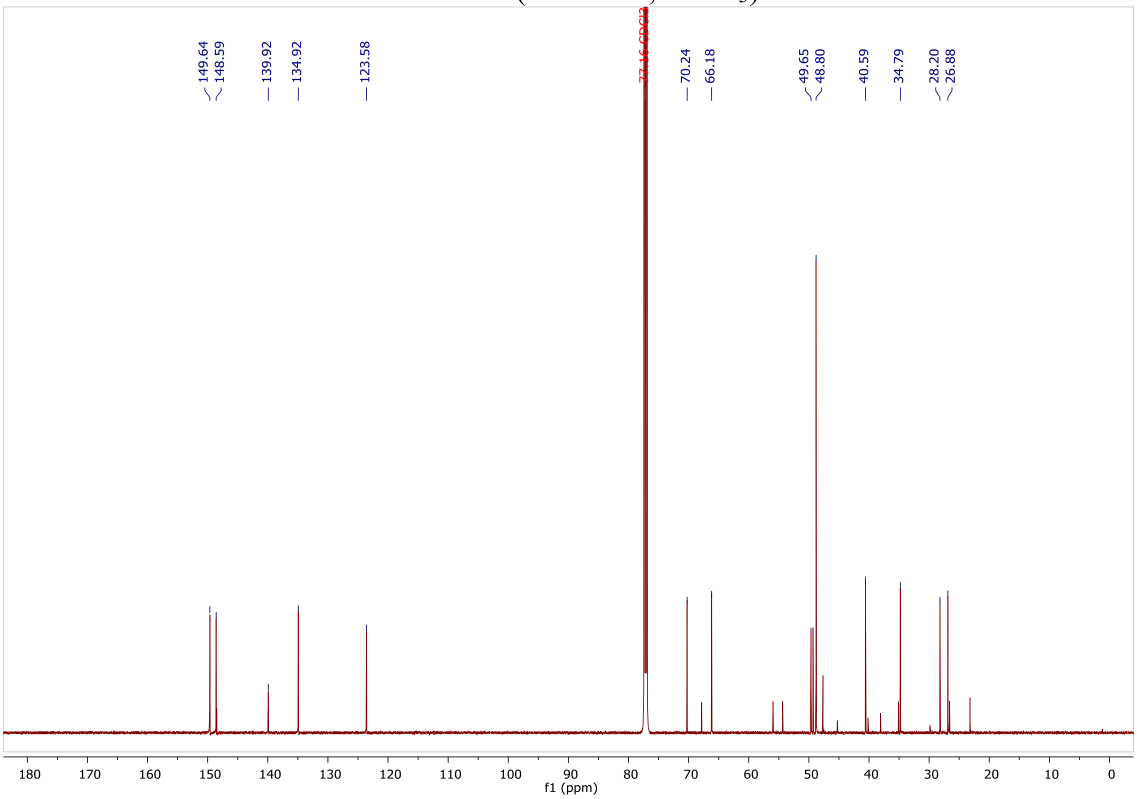

NOESY (600 MHz, $CDCl_3$)

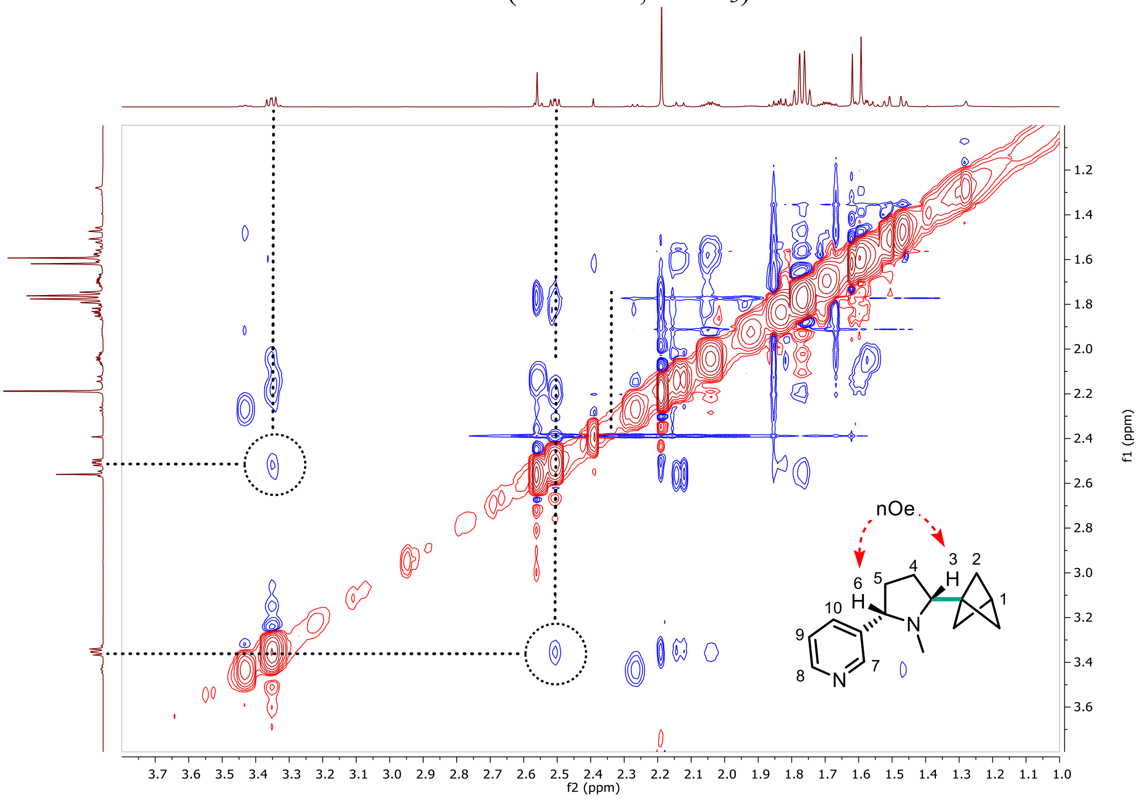

1-(4-(Methylsulfonyl)phenyl)pyrrolidine

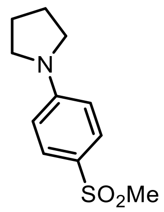

^{1}H NMR (400 MHz, CDCl$_3$)

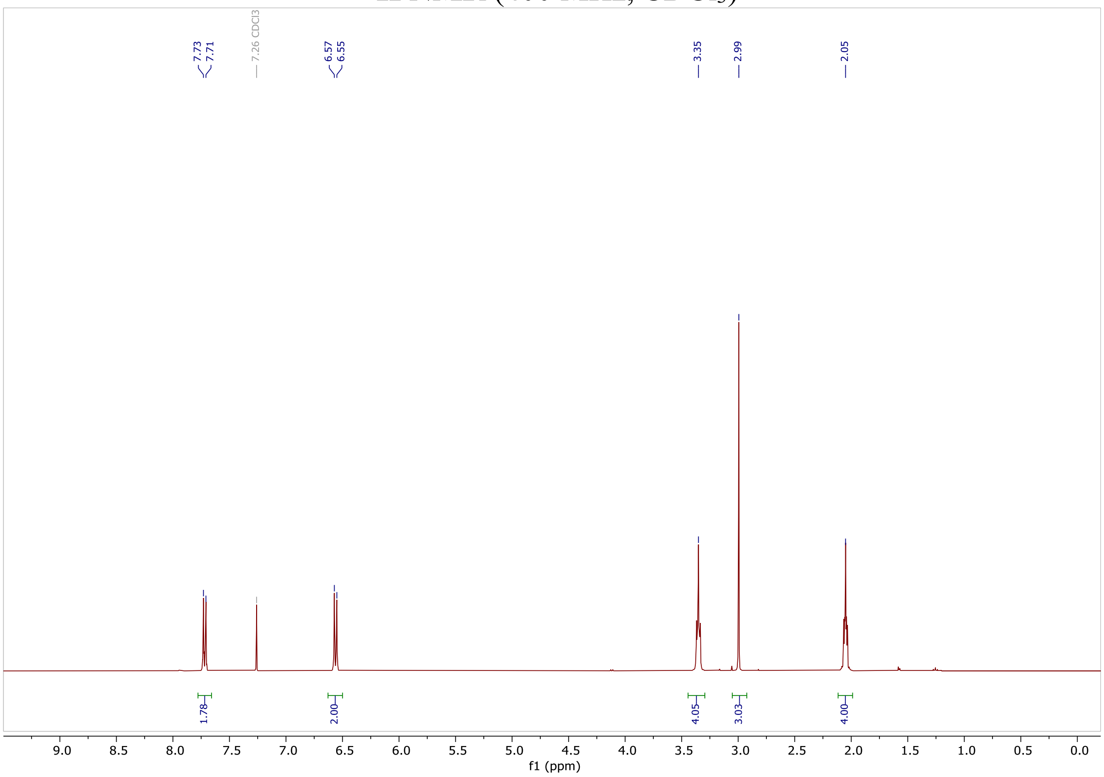

3-(Pyrrolidin-1-yl)benzonitrile

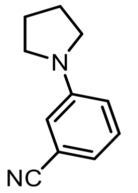

^{1}H NMR (400 MHz, $CDCl_3$)

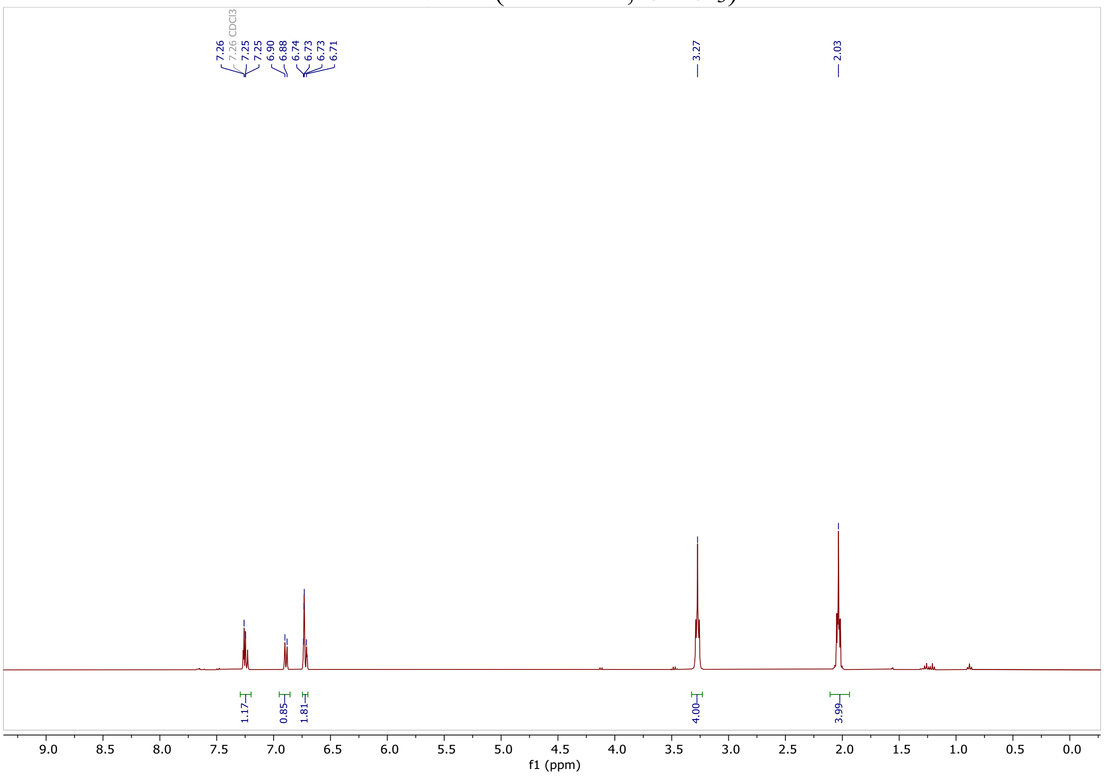

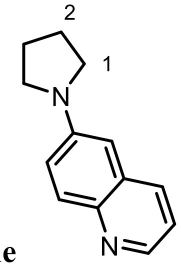

6-(pyrrolidin-1-yl)quinoline

^{1}H NMR (400 MHz, $CDCl_3$)

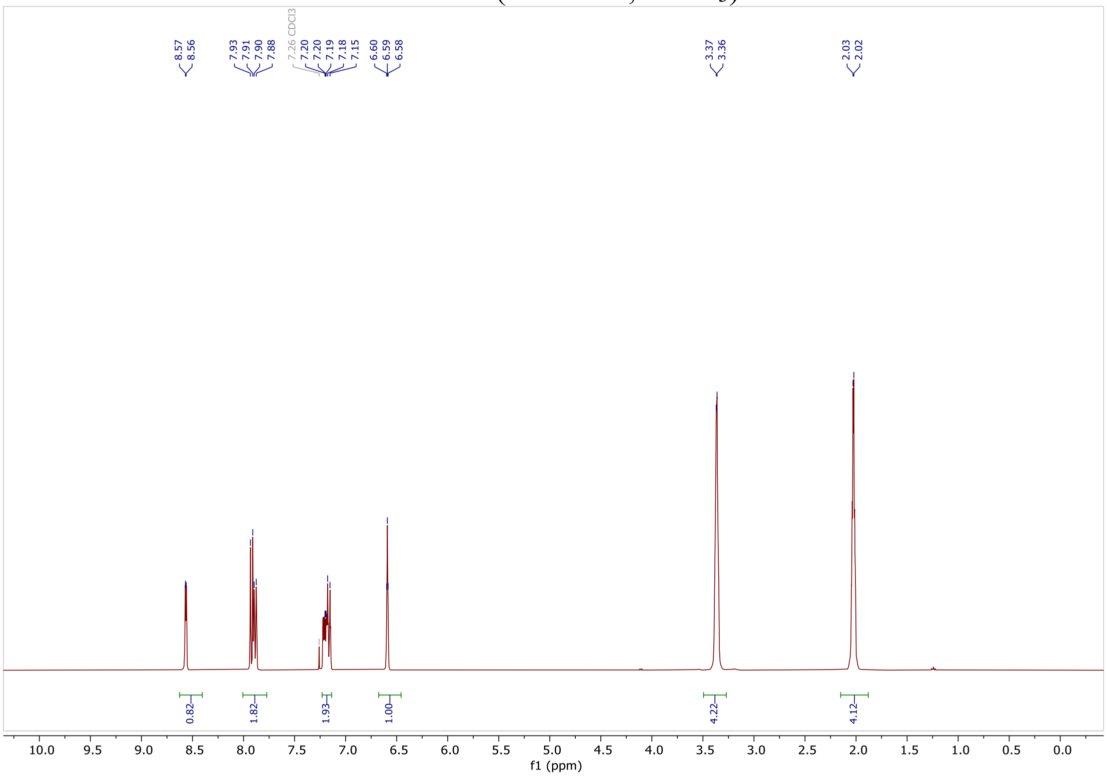

1-Phenylazepane

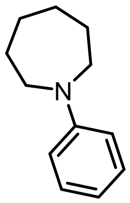

^{1}H NMR (400 MHz, $CDCl_3$)

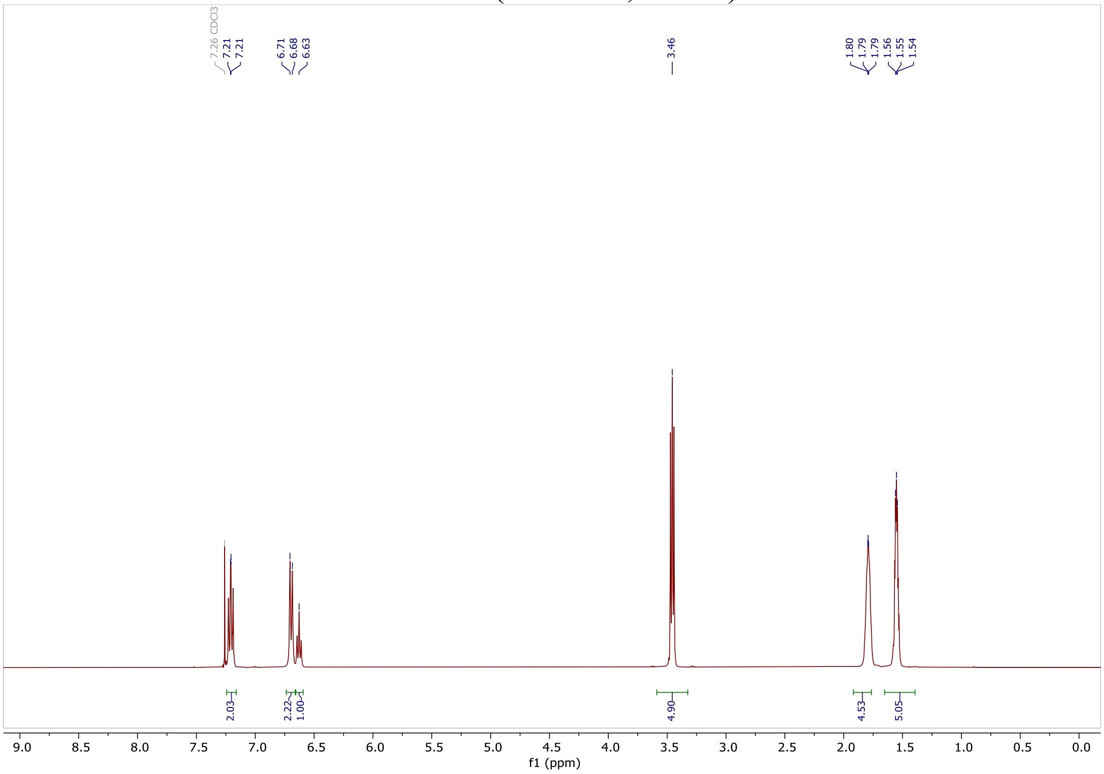

1-(Pyridin-3-yl)azepane

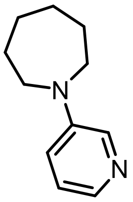

^{1}H NMR (400 MHz, $CDCl_3$)

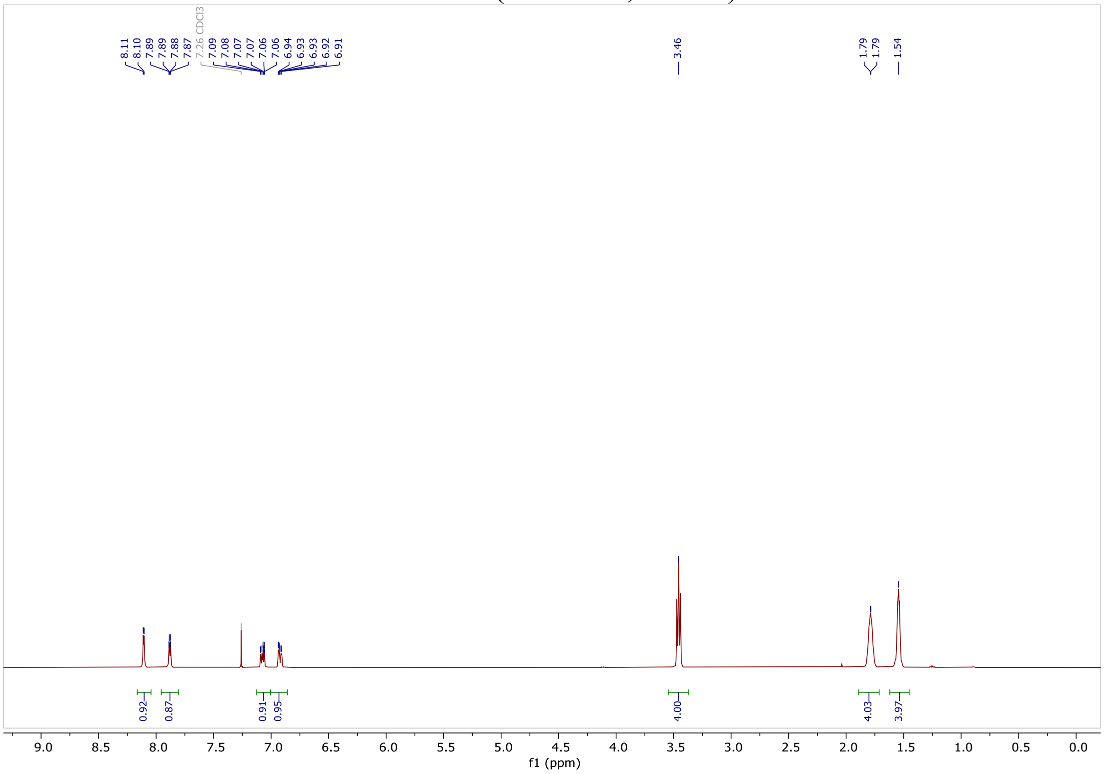

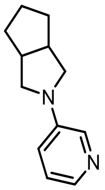

¹H NMR (400 MHz, $CDCl_3$)

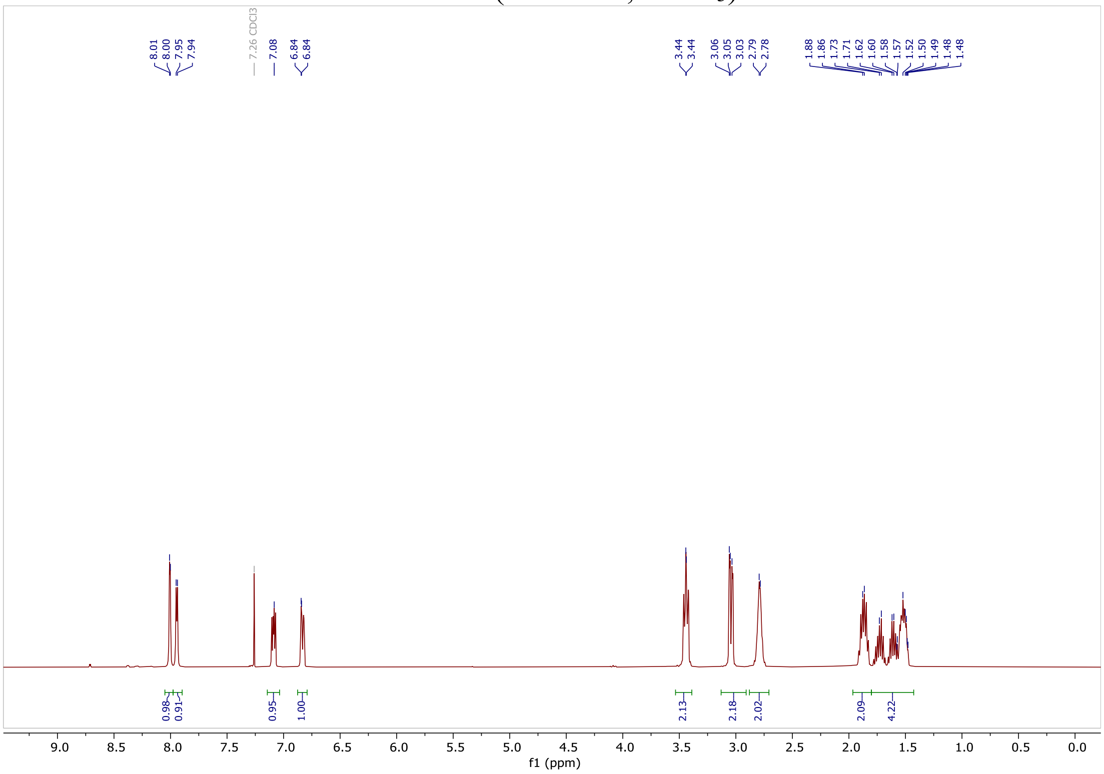

¹³C NMR (101 MHz, $CDCl_3$)

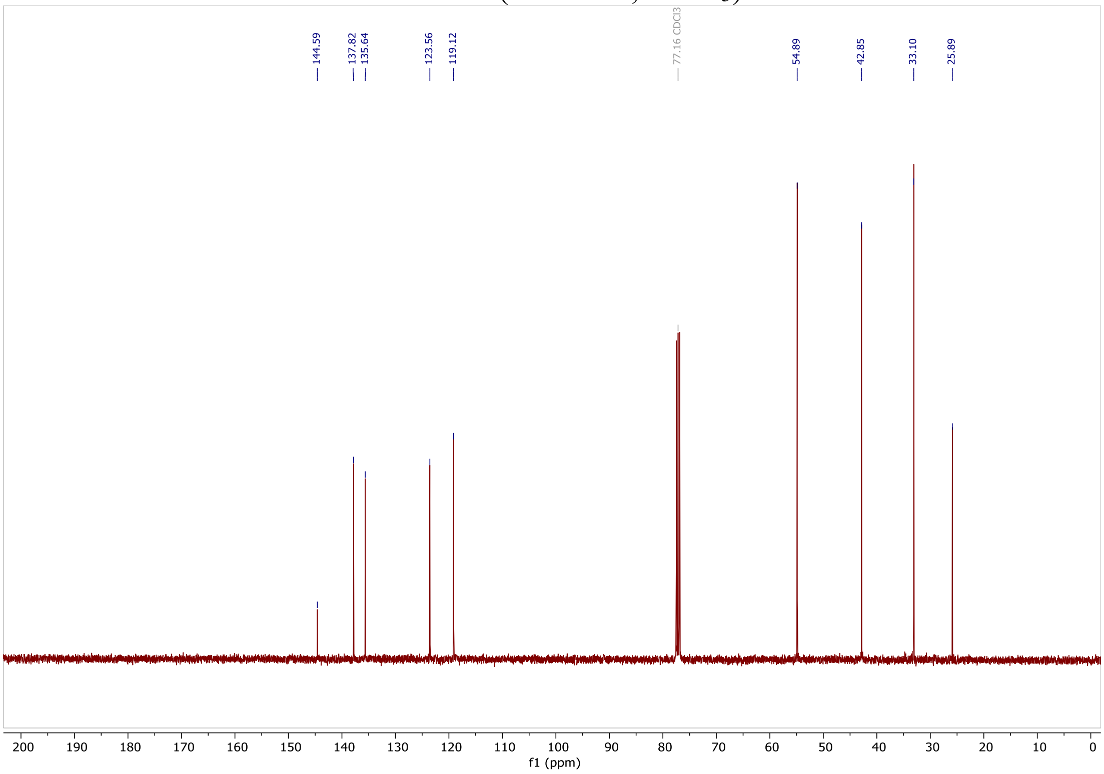

D4-*N*-phenylpyrollidine

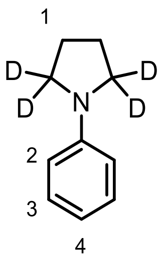

^{1}H NMR (400 MHz, $CDCl_3$)

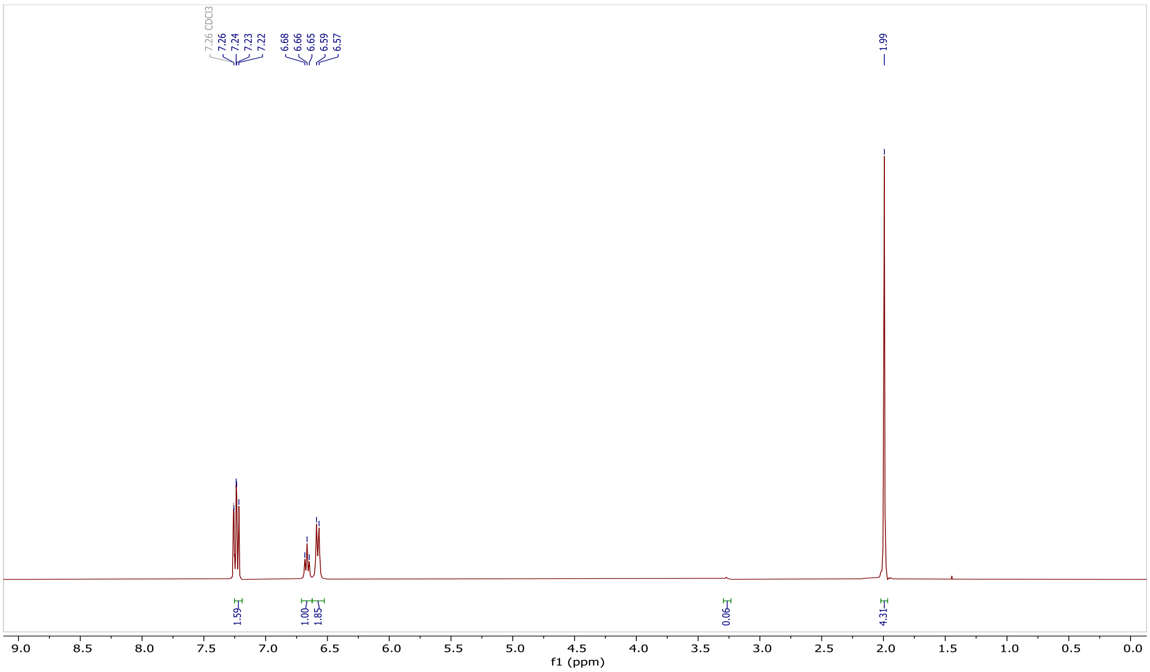

^{13}C NMR (101 MHz, $CDCl_3$)

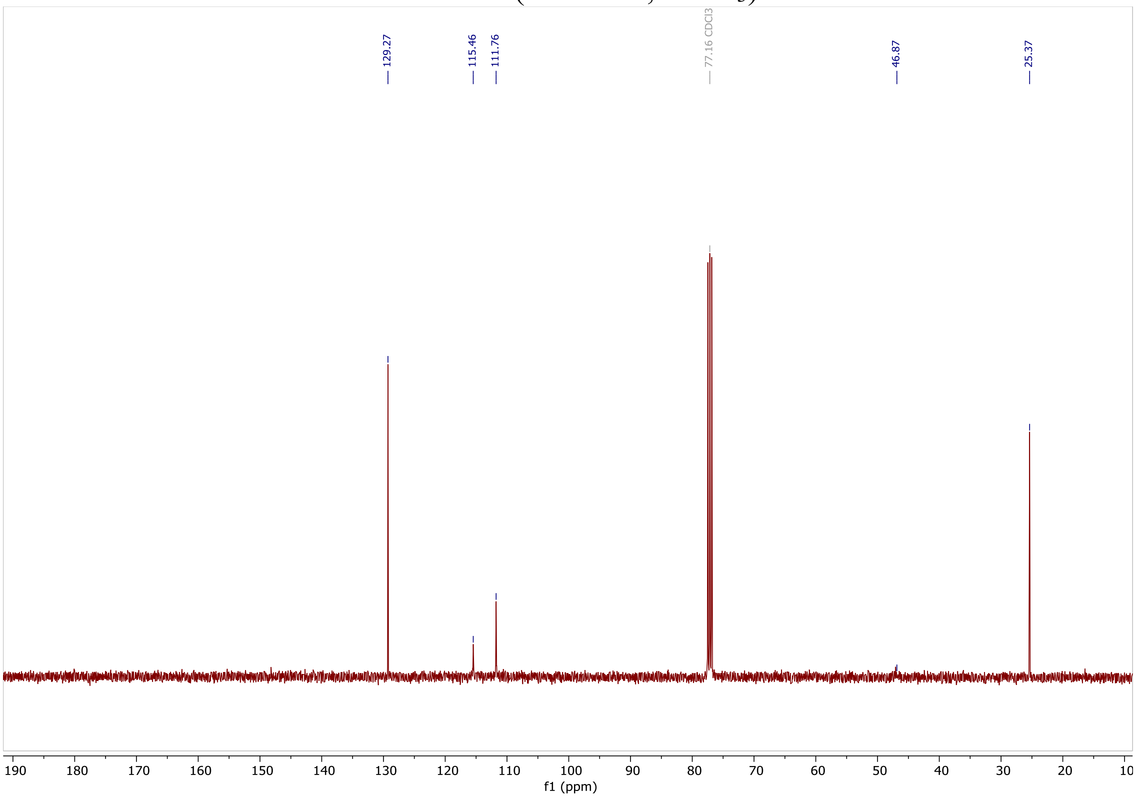

13. References

1. J. Nugent, C. Arroniz, B. R. Shire, A. J. Sterling, H. D. Pickford, M. L. J. Wong, S. J. Mansfield, D. F. J. Caputo, B. Owen, J. J. Mousseau, F. Duarte, E. A. Anderson, *ACS Catal.* **2019**, *9*, 9568-9574.
2. C. G. Hatchard, C. A. Parker, E. J. Bowen, *Proc. R. Soc. Lond. A* **1956**, *235*, 518-536.
3. Y.-Y. Liu, D. Liang, L.-Q. Lu, W.-J. Xiao, *Chem. Commun.* **2019**, *55*, 4853-4856.
4. J. P. Wolfe, H. Tomori, J. P. Sadighi, J. Yin, S. L. Buchwald, *J. Org. Chem.* **2000**, *65*, 1158-1174.
5. M. Tobisu, A. Yasutome, K. Yamakawa, T. Shimasaki, N. Chatani, *Tetrahedron* **2012**, *68*, 5157-5161.
6. Q. Cao, W. I. Nicholson, A. C. Jones, D. L. Browne, *Org. Biomol. Chem.* **2019**, *17*, 1722-1726.
7. J. H. Pang, A. Kaga, S. Chiba, *Chem. Commun.* **2018**, *54*, 10324-10327.
8. T. A. Young, J. J. Silcock, A. J. Sterling, F. Duarte, *Angew. Chem. Int. Ed.* **2020**, *60*, 4266-4274.
9. C. Bannwarth, E. Caldeweyher, S. Ehlert, A. Hansen, P. Pracht, J. Seibert, S. Spicher, S. Grimme, *Wiley Interdiscip. Rev. Comput. Mol. Sci.* **2020**, *11*, e1493.
10. M. Born, *Z. Phys.* **1920**, *1*, 45-48.
11. V. Barone, M. Cossi, *J. Phys. Chem. A* **1998**, *102*, 1995-2001.
12. S. Grimme, J. Antony, S. Ehrlich, H. Krieg, *J. Chem. Phys.* **2010**, *132*, 154104.
13. F. Weigenda, R. Ahlrichsb, *Phys. Chem. Chem. Phys.* **2005**, *7*, 3297-3305.
14. A. V. Marenich, C. J. Cramer, D. G. Truhlar, *J. Phys. Chem. B* **2009**, *113*, 6378-6396.
15. A. Karton, A. Tarnopolsky, J.-F. Lamère, G. C. Schatz, J. M. L. Martin, *J. Phys. Chem. A* **2008**, *112*, 12868-12886.
16. A. J. Sterling, A. B. Dürr, R. C. Smith, E. A. Anderson, F. Duarte, *Chem. Sci.* **2020**, *11*, 4895-4903.
17. Y. Zhao, D.G. Truhlar, *Theor. Chem. Acc.* **2008**, *120*, 215-241.
18. M. Isegawa, F. Neese, D. A. Pantazis, *J. Chem. Theory Comput.* **2016**, *12*, 2272-2284.
19. T. Young. (2020). duartegroup/otherm: Major symmetry improvements (1.0.0-beta). Zenodo.
20. Gaussian16, Revision C.01, M. J. Frisch, G. W. Trucks, H. B. Schlegel, G. E. Scuseria, M. A. Robb, J. R. Cheeseman, G. Scalmani, V. Barone, G. A. Petersson, H. Nakatsuji, X. Li, M.

Caricato, A. V. Marenich, J. Bloino, B. G. Janesko, R. Gomperts, B. Mennucci, H. P. Hratchian, J. V. Ortiz, A. F. Izmaylov, J. L. Sonnenberg, D. Williams-Young, F. Ding, F. Lipparini, F. Egidi, J. Goings, B. Peng, A. Petrone, T. Henderson, D. Ranasinghe, V. G. Zakrzewski, J. Gao, N. Rega, G. Zheng, W. Liang, M. Hada, M. Ehara, K. Toyota, R. Fukuda, J. Hasegawa, M. Ishida, T. Nakajima, Y. Honda, O. Kitao, H. Nakai, T. Vreven, K. Throssell, J. A. Montgomery Jr., J. E. Peralta, F. Ogliaro, M. J. Bearpark, J. J. Heyd, E. N. Brothers, K. N. Kudin, V. N. Staroverov, T. A. Keith, R. Kobayashi, J. Normand, K. Raghavachari, A. P. Rendell, J. C. Burant, S. S. Iyengar, J. Tomasi, M. Cossi, J. M. Millam, M. Klene, C. Adamo, R. Cammi, J. W. Ochterski, R. L. Martin, K. Morokuma, O. Farkas, J. B. Foresman, D. J. Fox. Gaussian Inc., Wallingford CT, 2016.

21. R. Cammi, B. Mennucci, J. Tomasi, *J. Phys. Chem. A*, **2000**, *104*, 5631-5637.

22. J. Tomasi, B. Mennucci, R. Cammi, *Chem. Rev.* **2005**, *105*, 2999-3093.

23. H. G. Roth, N. A. Romero, D. A. Nicewicz, *Synlett* **2016**, 27, 714-723.

24. N. Bortolamei, A. A. Isse, A. Gennaro, *Electrochim. Acta* **2010**, *55*, 8312-8318.

25. J. R. Ames, S. Brandänge, B. Rodriguez, N. Castagnoli, M. D. Ryan, P. Kovacic, *Bioorg. Chem.* **1986**, *14*, 228-241.
